# Lobular architecture of human adipose tissue defines the niche and fate of progenitor cells

**DOI:** 10.1038/s41467-019-09992-3

**Published:** 2019-06-11

**Authors:** D. Estève, N. Boulet, C. Belles, A. Zakaroff-Girard, P. Decaunes, A. Briot, Y. Veeranagouda, M. Didier, A. Remaury, J. C. Guillemot, S. Ledoux, C. Dani, A. Bouloumié, J. Galitzky

**Affiliations:** 1 Inserm, UMR 1048, Team 1, I2MC, Institute of Metabolic and Cardiovascular Diseases, Université de Toulouse, BP84225, F-31432 Toulouse Cedex 4, France; 2 Inserm, UMR 1048, Cytometry Platform, I2MC, Institute of Metabolic and Cardiovascular Diseases, BP84225, F-31432 Toulouse Cedex 4, France; 3 Sanofi Aventis Research & Development, Translational Sciences, 91385 Chilly-Mazarin Cedex, France; 4 Center Support of Obesity, Louis Mourier Hospital, Université Paris Diderot, F-92700 Colombes, France; 5 Université Côte-d’Azur, Inserm, CNRS iBV, Nice, France

**Keywords:** Fat metabolism, Mesenchymal stem cells

## Abstract

Human adipose tissue (hAT) is constituted of structural units termed lobules, the organization of which remains to be defined. Here we report that lobules are composed of two extracellular matrix compartments, i.e., septa and stroma, delineating niches of CD45−/CD34+/CD31− progenitor subsets characterized by MSCA1 (ALPL) and CD271 (NGFR) expression. MSCA1+ adipogenic subset is enriched in stroma while septa contains mainly MSCA1−/CD271− and MSCA1−/CD271^high^ progenitors. CD271 marks myofibroblast precursors and NGF ligand activation is a molecular relay of TGFβ-induced myofibroblast conversion. In human subcutaneous (SC) and visceral (VS) AT, the progenitor subset repartition is different, modulated by obesity and in favor of adipocyte and myofibroblast fate, respectively. Lobules exhibit depot-specific architecture with marked fibrous septa containing mesothelial-like progenitor cells in VSAT. Thus, the human AT lobule organization in specific progenitor subset domains defines the fat depot intrinsic capacity to remodel and may contribute to obesity-associated cardiometabolic risks.

## Abstract (web summary)

In human adipose tissue (AT), adipocytes are organized into units of lobules. Here the authors identify distinct fibrous septal and stromal compartments within AT lobules that differ in composition and potential between subcutaneous and visceral regions and are disturbed in obesity and metabolic syndrome.

## Introduction

Fat mass repartition is a major determinant for health^1^. Central obesity, characterized by fat mass repartition in favor of visceral depots, is a risk factor for the development of cardiometabolic disorders^2^. The portal hypothesis explains the deleterious impact of visceral (VS) fat by its direct anatomical connection with the liver through the portal vein, promoting liver steatosis and insulin resistance^3^. In addition, the concept of adipose tissue (AT) expandability considers subcutaneous (SC) fat as protective against lipotoxicity, thanks to the storage of fatty acids into neutral triglycerides in mature adipocytes^4^. The intrinsic property of a fat depot to handle lipids in excess (or its expandability) is not only determined by the metabolic fitness of mature adipocytes but also its capacity to produce new adipocytes by adipogenesis^5^. The ability of a mature adipocyte to increase lipid storage by hypertrophy is limited by a maximal size, according to pioneer studies of P. Bjorntorp^6^. This maximal size may be controlled by local physical constraints, including the ones generated by the excessive extracellular matrix deposition surrounding adipocytes^7^. Therefore, fibrosis may be viewed as deleterious restricting adipocyte hypertrophy^8^. In contrast, adipogenesis is protective, improving AT function via the renewal of the adipocyte pool and AT expandability by hyperplasia. Adipogenesis is a sequential cellular event starting from immature mesenchymal progenitor cells that once committed into preadipocytes differentiate into adipocytes. In adult human AT, we and others identified by flow cytometry and cell sorting the local pool of mesenchymal progenitor cells as CD45−/CD34+/CD31− cells^9,10^. In particular, we found that the expression of MSCA1^11^ and CD36^12^ further delineates the preadipocyte subset. In addition to commitment into an adipogenic lineage, human AT progenitor cells exhibit a myofibroblastic potential in response to TGFβ1 stimulation and are therefore suspected to be involved in fibrosis^13,14^.

It is now well established that the niche of progenitor cells is a key component for fate determination^15^. During development, subsets of mesenchymal progenitors are organized into specific domains defining lineages^16^. In murine AT, cell-tracing approaches have shown that adipocytes may not only arise from progenitor cells present at the perivascular position^17^ but also from the mesothelium^18^. The intrinsic adipogenic capacity is different according to fat depot location^19^. Such differences have been speculated to be due to cells arising from distinct developmental origin^20^ and/or from the local microenvironment, including the extracellular matrix^21^. When the differentiation potential of human AT progenitor cells is taken into account, i.e., adipogenic and myofibroblastic, as well as the fat depot differences according to development and function, we hypothesized that progenitor cells in human SC and VSAT are organized in distinct niches containing cell subsets defined by different commitment and differentiation states. We provide evidence that the architecture of the human fat depots into lobule units delineates two structural extracellular matrix compartments and niches of progenitor cell subsets. We highlight differences in the macro-architecture associated with distinct surface markers and fate of progenitor cell subsets in SC and VSAT. We suggest that such differences contribute to the intrinsic capacity of healthy/unhealthy expansion according to fat depot.

## Results

### Septa and stroma of a lobule delineate progenitor niches

**Fig. 1:**
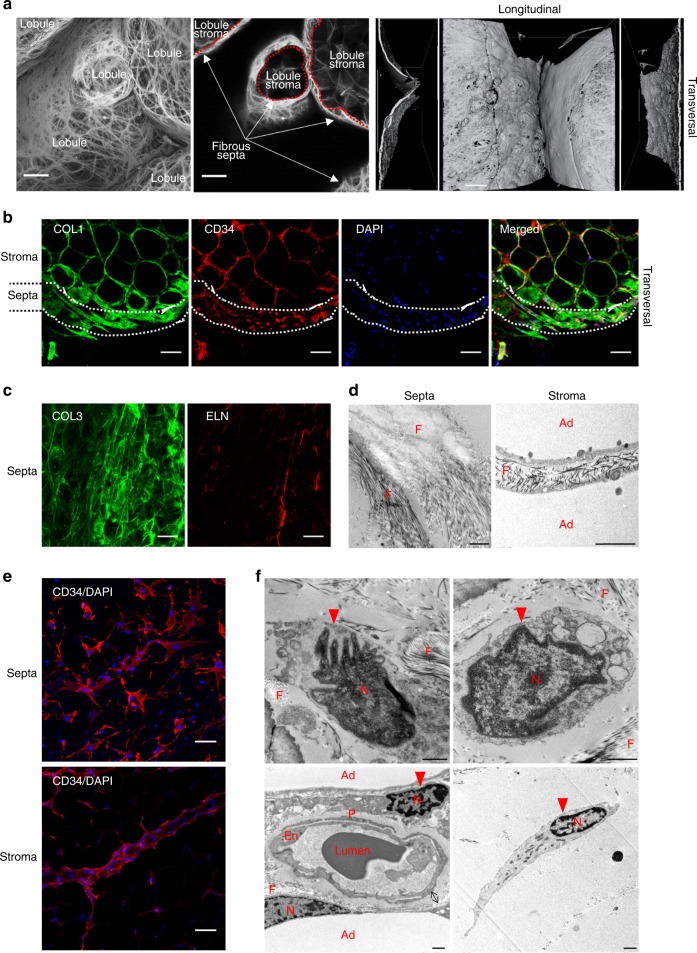
Macro- and micro-architecture of human adipose tissue lobule. **a** Representative three-dimensional image of the collagen network (picrosirius red) of human adipose tissue (AT) lobules with surface reconstruction of the longitudinal and transversal views, fibrous septa, and stroma are underlined, scale bars: 100 µm. **b** Representative immunostaining of the human AT lobule with COLLAGEN 1 (COL1), CD34, and DAPI. The position of the septa is underlined, scale bar: 100 µm. **c** Representative immunostaining of the human AT lobule septa with collagen 3 (COL3) and ELASTIN (ELN), white scale bars: 50 µm. **d** Electronic microcopy analyses performed on human subcutaneous adipose lobule septa and stroma (F: extracellular matrix fibers, Ad: mature adipocytes), black scale bars: 1 µm. **e** Representative immunostaining with CD34 and DAPI of the subcutaneous lobule septa and stroma, white scale bars: 100 µm. **f** Electronic microcopy analyses performed on the human subcutaneous adipose lobule septa (upper panel) and stroma (lower panel) (Ad: mature adipocytes, F: extracellular matrix fibers, En: endothelial cells, P: pericyte N: nucleus, red arrow: progenitor cell, black arrow: basement membrane), black scale bars: 1 µm

**Fig. 2:**
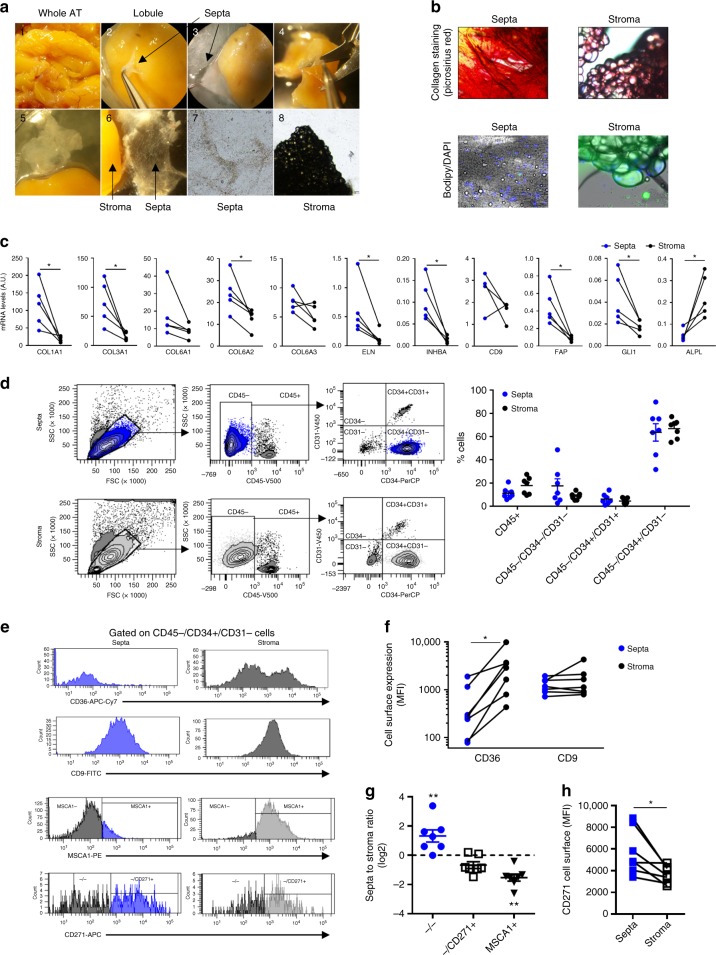
CD34+ cells characterization in the fibrous septa and stroma. **a** Microdissection of AT septa and stroma. A piece of the whole AT was rinsed with PBS (image 1), and lobules were isolated one by one. Isolated lobules were precisely dissected using Dumont forceps and Vannas spring scissors under a Zeiss StemiV6 stereomicroscope at ×8 magnification. The septa surrounding the lobule were progressively lifted off (images 2–5) until its complete separation from the stroma (image 6). The aspect of dissected septa (fibrous membrane without mature adipocytes) and stroma (mature adipocytes without fibrous membrane) are shown in images 7 and 8, respectively, under a bright-field microscope with a ×40 magnification. **b** Representative microphotographs of dissected septa and stroma stained with picrosirius red (upper panel) and Bodipy/DAPI (lower panel). **c** Gene expression in non-adipose cells isolated from matched fibrous septa (blue) and stroma (black) from subcutaneous AT lobules determined by RT-qPCR analyses. The results are means ± s.e.m. of experiments performed on *n* = 5 independent donors, paired *t* test, *P < 0.05. **d** Representative dot plots obtained by flow cytometry analyses with SVF cells isolated from septa and stroma using anti-CD45, -CD31, and -CD34 antibodies and quantification of the CD45+, CD45−/CD34−/CD31−, CD45−/CD34+/CD31+,  and CD45−/CD34+/CD31− cell populations in the septa (blue) and stroma (black). The results are means ± s.e.m. of experiments performed on *n* = 7 independent donors, two-way ANOVA. **e** Representative histograms of fluorescence intensity of CD36, CD9, MSCA1, and CD271 on CD45−/CD34+/CD31− cells and **f** CD36 and CD9 mean fluorescence intensity (MFI) determined by flow cytometry analyses performed on matched fibrous septa (blue) and stroma (black) cells (*n* = 7, paired *t* test, *P < 0.05). **g** Repartition of CD45−/CD34+/CD31− progenitor cell subsets (i.e., −/−, −/CD271+, and MSCA1+) in matched septa and stroma determined by flow cytometry analyses. The results are expressed as log2 ratio of septa to stroma percentages and are means ± s.e.m. of experiments performed on *n* = 7 independent donors. One-way ANOVA followed by Dunnett’s multiple comparison test, **P < 0.01. **h** CD271 MFI determined by flow cytometry analyses on the −/CD271+ cells from septa (blue) and stroma (black) from *n* = 7 independent donors, paired *t* test, *P < 0.05

To analyze the macrostructure of the human subcutaneous AT lobule, we performed picrosirius red staining and immunostaining on whole AT biopsies using confocal and transmission electron microscopy imaging. Picrosirius red staining delineated two main fibrillar collagen matrix compartments in lobules: the stroma as well as the fibrous septa that was well individualized as shown by transversal view (Fig. 1a). Immunostaining of COLLAGEN 1 (COL1) underlined both compartments with a marked accumulation of COLLAGEN 1 in the septa compared with the stroma, while CD34 immunostaining revealed the presence of CD34+ cells in both compartments (Fig. 1b). In addition to COLLAGEN 1, COLLAGEN 3 (COL3) and ELASTIN (ELN) fibers were positively stained in lobule septa (Fig. 1c). Electron microscopy approaches clearly showed dense networks of the extracellular matrix fibers in the septa, while the extracellular fibers in the stroma between adipocytes were sparse (Fig. 1d). The CD34+ cells exhibited compartment-dependent morphology: flattened in the septa while spindle shaped in the stroma (Fig. 1e). Electron microscopy approaches highlighted cells with a large cytoplasm in the septa (Fig. 1f). In the stroma, elongated cells were positioned either between adipocytes or at the perivascular location, although they were not embedded in the capillary wall, as it is the case for pericytes (Fig. 1f). Subcutaneous AT lobules were carefully dissected (Fig. 2a). Septa surrounding the lobule were progressively lifted off until complete separation from the stroma (Fig. 2a). Dissected septa were stained positive with picrosirius red as a fibrous structure and negative with Bodipy (without mature adipocytes). The staining pattern was the reverse for the lobule stroma containing mature adipocytes (Fig. 2b). Transcriptional analyses were performed by quantitative real-time PCR (RT-qPCR) on cells after collagenase digestion of both septa and stroma and separation of the non-adipose cells from stroma mature adipocytes (Fig. 2c). A marked enrichment in transcript levels encoding fibrillar extracellular matrix-related genes, including *COL1A1* (collagen type I α-1 chain), *COL3A1* (collagen type III α-1 chain), and *ELN* (ELASTIN) was found in septa compared with stroma cells (Fig. 2c). The mRNA expression levels for non-fibrillar collagens, including *COL6A1* and *COL6A3* (collagen type VI α-1, -2, and -3 chains), exhibited no compartment-dependent profile, while the COL6A2 was enriched in the septa (Fig. 2c). In addition, septa cells expressed high levels of myofibroblast precursor’s markers such as *GLI1* (GLI Family Zinc Finger 1) and *FAP* (fibroblast activation protein) as well as *INHBA* (inhibin subunit β A), while the expression of human preadipocyte marker *ALPL* (encoding for MSCA1) was enriched in the stroma (Fig. 2c). To note, the expression of CD9 recently described to be involved in fibrosis^14^ was not different between the two compartments. To further characterize septa and stroma cells, flow cytometry analysis using a multicolor panel of cell-surface markers (CD45, CD31, CD34, CD36, CD9, MSCA1, and CD271) was performed. The gating strategy, including fluorescence-minus-one approaches, is shown in Supplementary Fig. 1. The repartition of the main cell subtypes, including CD45+ immune cells, CD45−/CD34+/CD31+ endothelial cells, CD45−/CD34−/CD31− mural vascular cells, and CD45–/CD34+/CD31− progenitor cells, was not different between septa and stroma. The main cell population was progenitor cells in both compartments (Fig. 2d). While CD9 expression did not exhibit differences, the one of CD36 was higher in stroma compared with septa progenitor cells (Fig. 2e, f). In agreement with a specific stromal niche of the progenitor cells with high adipogenic potential, MSCA1+ cells were clearly enriched in the stroma (Fig. 2e–g). Conversely, the lobule septa were markedly enriched in the CD34+ subset negative for both MSCA1 and CD271 (−/− cells) (Fig. 2g). The MSCA1−/CD271+ (−/CD271+) cells were equally distributed between the two lobule compartments, but the expression level of CD271 itself was higher in the septa than stroma cells (Fig. 2e–h). Therefore, the progenitor cells (CD45−/CD34+/CD31−) in human AT are localized in two niches, the stroma with the high adipogenic CD36+/MSCA1+/CD34+ cells and the fibrous septa containing the −/− and −/CD271^high^ progenitor cells.

### SC progenitor subsets display distinct potentials

**Fig. 3:**
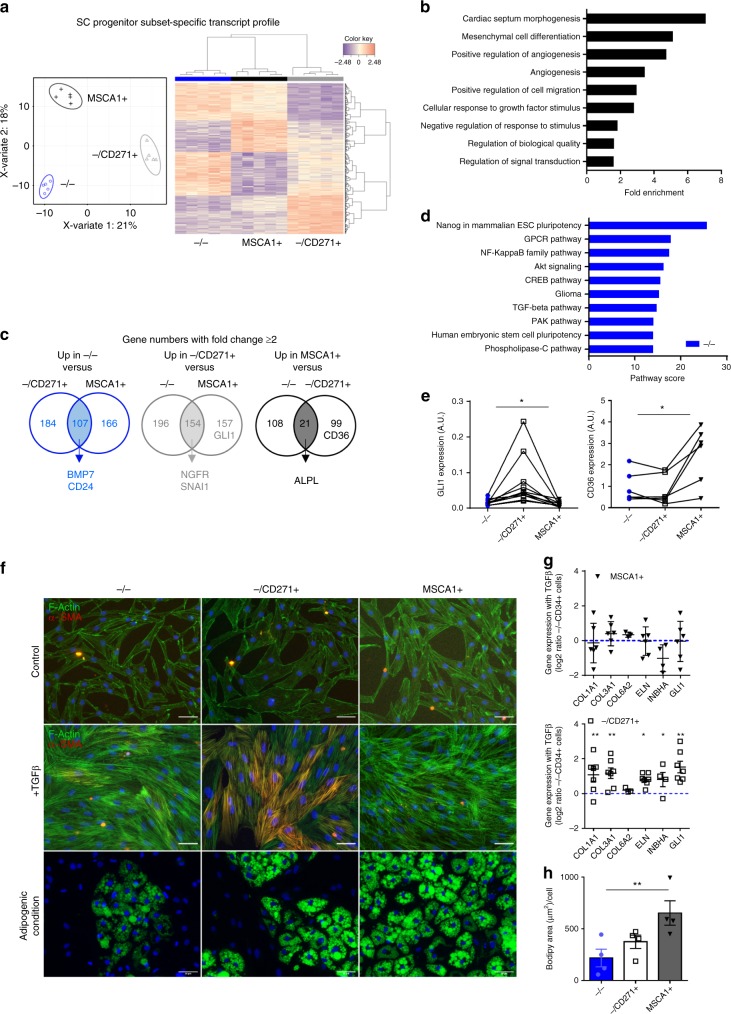
Myofibroblast precursors are identified in the CD271+ subset. RNAseq analysis was performed on paired progenitor subsets isolated from SCAT of five independent obese donors. **a** Sparse partial least-square discrimant analysis plot of the two principal components. The 95% confidence ellipses are shown to strengthen the cell population clustering (left) and heatmap (right) of the top 400 most differentially expressed genes defining a progenitor subset-specific profile. Color code is set up according to gene expression (high expression in red, low in blue). **b** Gene ontology pathways determined on the 400 progenitor subset-specific genes. **c** Venn diagrams of differentially expressed genes (fold change ≥ 2 from a raw *P*-value) in −/− cells vs. −/CD271+ and MSCA1+ (left), in −/CD271+ cells vs. −/− and MSCA1+ (middle) and in MSCA1+ cells vs. −/− and −/CD271+ (right), highlighting some genes of interest. **d** Top ten superpathways determined from SC −/− subset-specific transcripts. **e** *GLI1* and *CD36* gene expression in native progenitor subsets determined by RT-qPCR analyses. The results are means ± s.e.m. of experiments performed on *n* = 6 (*CD36*) and *n* = 11 (*GLI1*) independent donors, one-way ANOVA followed by Tukey’s multiple comparison test, *P < 0.05. **f** Sorted SC progenitor subsets were cultured in the presence or absence of 5 ng/mL of TGFβ1 for 4 days, and immunostaining approaches were performed using phalloidin (F-ACTIN, green), αSMA antibody (red), and DAPI (blue), or cultured under adipogenic condition and stained with Bodipy (green) and DAPI (blue). Representative fluorescence microscopy images are shown, scale bar: 50 µm. **g** Gene expression levels in SC immunoselected progenitor subsets (−/−, −/CD271+, and MSCA1+ cells) treated with TGFβ1 were determined by RT-qPCR analyses. The results are expressed as log2 ratio of paired MSCA1+ or −/CD271+ cells to −/− cells mRNA levels and are means ± s.e.m. of experiments performed on *n* = 3 (COL6A2) to *n* = 7 independent donors, one-way ANOVA followed by Tukey’s multiple comparison test, *P < 0.05, **P < 0.01. **h** Quantification of lipid accumulation in SC progenitor subsets at day 10, the results are means ± s.e.m. of experiments performed on *n* = 4 independent donors. One-way ANOVA followed by Dunnett’s multiple comparison test, **P < 0.01

RNA sequencing (RNAseq) was performed on matched native −/−, −/CD271+, and MSCA1+ subsets isolated from subcutaneous (SC) AT of five distinct obese donors. Paired sparse partial least-squares discriminant (sPLS-D) analyses discriminated transcripts exhibiting progenitor subset-specific profiles clustering −/− subset with MSCA1+ subset followed by −/CD271+ subset (Fig. 3a). The progenitor subset-specific transcripts were enriched in pathways related to cellular and developmental processes, including mesenchymal cell differentiation (Fig. 3b). Gene expression comparisons between subsets (two or more fold changes) highlighted 107 transcripts specifically upregulated in −/− subset, 154 in −/CD271+ subset, and 21 in MSCA1+ subset (Fig. 3c; Supplementary Table 2). As expected, *NGFR* (encoding for CD271) but also *SNAI1*, as well as *ALPL* (encoding for MSCA1) were identified in, respectively, −/CD271+ and MSCA1+ specific genes. *BMP7*, that we already described as expressed and produced by −/− subset^11^ and *CD24*, a murine marker for non-committed adipose precursor^22^, were highly expressed in −/− specific transcripts together with enrichment in pathways related to embryonic stem cell pluripotency (Fig. 3c, d). The myofibroblast precursor marker *GLI1* allowed to discriminate among 157 other genes −/CD271+ subset from MSCA1+ subset, while *CD36* expression was among 99 other transcripts specifically upregulated in MSCA1+ compared with −/CD271+ cells (Fig. 3c). The enrichment of *GLI1* in −/CD271+ cells and *CD36* in MSCA1+ was confirmed by RT-qPCR performed on paired progenitor subsets from other donors (Fig. 3e). Sorted subsets were cultured in serum-deprived condition and treated once with 5 ng/mL TGFβ1 for 4 days or maintained in adipogenic culture condition for 10 days. As shown in Fig. 3f, the three cell subsets responded to TGFβ1 treatment with a marked reorganization of the actin cytoskeleton (F-ACTIN). However, the −/CD271+ progenitor subset was the only subset that differentiated into αSMA-positive cells in response to TGFβ1 treatment. The detection of αSMA-positive cells in the −/CD271+ population treated with TGFβ1 was accompanied by changes in the expression of fibrillar matrix-related transcripts (*COL1A1*, *COL3A1*, and *ELN*) together with *INHBA* and *GLI1* expression that contrasted with −/− and MSCA1+ progenitor cells (Fig. 3g). Conversely and as expected, the MSCA1+ cells exhibited the highest capacity to differentiate into lipid-laden cells shown by Bodipy staining and lipid accumulation quantification (Fig. 3f–h).

### TGFβ1 induces myofibroblastic conversion via the CD271/NGF axis

**Fig. 4:**
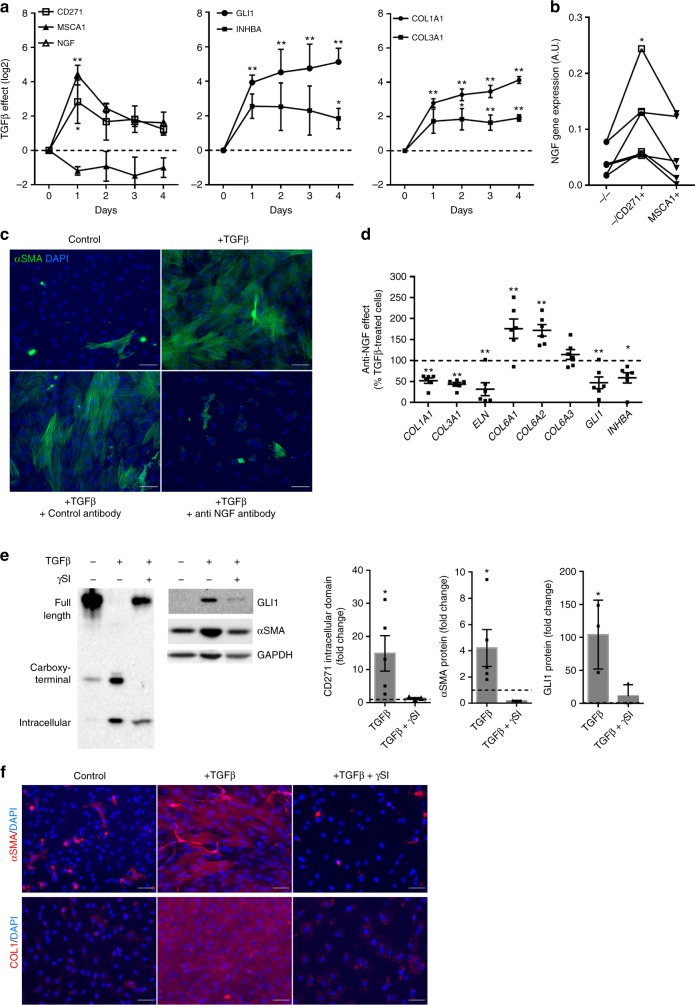
Myofibroblast differentiation requires CD271/NGF activation. **a** Gene expression in whole SCAT progenitor cells treated for the indicated times with 5 ng/mL of TGFβ1 determined by RT-qPCR. The results are expressed as log2 gene expression ratio of treated to non-treated cells and are means ± s.e.m. of experiments performed on *n* = 4 (day 0 to day 3) to *n* = 12 (day 4) independent donors. Two-way ANOVA followed by Tukey’s multiple comparison test, *P < 0.05, **P < 0.01. **b** *NGF* mRNA levels in matched native progenitor cell subsets from *n* = 6 independent donors. One-way ANOVA, *P < 0.05. **c** Representative αSMA immunostaining (green) with DAPI (blue) on AT progenitor cells non-treated (control), treated with TGFβ1 in the presence or absence of either control antibody or anti-NGF antibody for 4 days. Scale bars: 50 µm. **d** Transcript levels of genes of interest were measured by RT-qPCR. The results are expressed as percentages of TGFβ-treated cells and are means ± s.e.m. of experiments performed on *n* = 6 independent donors. Two-way ANOVA followed by Bonferroni’s multiple comparison test, *P < 0.05, **P < 0.01. **e** Representative western blot analysis performed on progenitor cells treated with 5 ng/mL of TGFβ1 for 4 days in the presence or absence of gamma-secretase inhibitor (γSI, 25 µmol/L). Left panel: CD271 isoform (full length, the carboxy-terminal domain and intracellular domain) detection, right panel: GLI1, αSMA, and GAPDH detection. The CD271 intracellular domain protein, αSMA, and GLI1 expressions were quantified and normalized to GAPDH. The results are expressed as fold changes compared with control cells and are means ± s.e.m. of *n* = 3 (TGFβ1 + γSI) and *n* = 5 (control and TGFβ1) independent experiments. One-way ANOVA followed by Dunnett’s multiple comparison test, *P < 0.05. **f** Representative αSMA (red) and COLLAGEN 1 (red) immunostaining with DAPI (blue) in non-treated (control), TGFβ-treated cells with or without gamma-secretase inhibitor. Scale bars: 50 µm

To further delineate the involvement of CD271 activation in the myofibroblast differentiation, subcutaneous CD34+ progenitor cells were treated once with TGFβ1 for 1–4 days. Whatever the time point, the expression of MSCA1 (*ALPL*) was not modified in the presence of TGFβ1 (Fig. 4a). The transcript levels of *CD271* and its main ligand *NGF* were markedly induced as early as 1 day of treatment. Both *CD271* and *NGF* decreased the following day, while the induction of fibrillar collagen transcripts (*COL1A1* and *COL3A1*) as well as myofibroblast markers *GLI1* and *INHBA* was maintained elevated. Therefore, TGFβ1, without affecting MSCA1 expression, promotes an increase in *CD271* and its ligand *NGF* as well as *GLI1* expression. This is followed by the acquisition of mature myofibroblast phenotype characterized by an enhanced fibrillar collagen and *INHBA* expression. To note, *NGF* expression was higher in the −/CD271+ isolated progenitors compared with MSCA1+ and −/− subsets in their native state (Fig. 4b). To determine whether the CD271/NGF axis upregulation is involved in myofibroblast differentiation, cells were stimulated with TGFβ1 in the presence or absence of a neutralizing antibody directed against NGF. As shown in Fig. 4c, neutralization of NGF completely inhibited the TGFβ1-induced promotion of αSMA-positive cells as well as the expression of fibrillar matrix-related transcripts (*COL1A1*, *COL3A1*, and *ELN*) and myofibroblast markers *GLI1* and *INHBA* (Fig. 4d). The expression of non-fibrillar collagens (*COL6A1*, *A2*, and *A3*) was not inhibited in the presence of an anti-NGF antibody. The activation of CD271 by its ligand NGF requires proteolytic cleavage of the receptor by the gamma-secretase complex releasing its intracellular domain^23^. Western blot analyses revealed that TGFβ1 led to a 15-fold increase in the CD271 intracellular domain that was inhibited in the presence of the gamma-secretase inhibitor (γSI) (Fig. 4e). In parallel, γSI treatment markedly decreased TGFβ1-induced GLI1 and αSMA protein (Fig. 4e). Finally, the addition of γSI to TGFβ1 treatment completely abrogated the appearance of αSMA and type I collagen-positive myofibroblasts (Fig. 4f). Therefore, the activation of CD271-dependent pathway is a required step in the TGFβ1-mediated myofibroblast differentiation.

### SC and VS lobules have different macro-architectures

**Fig. 5:**
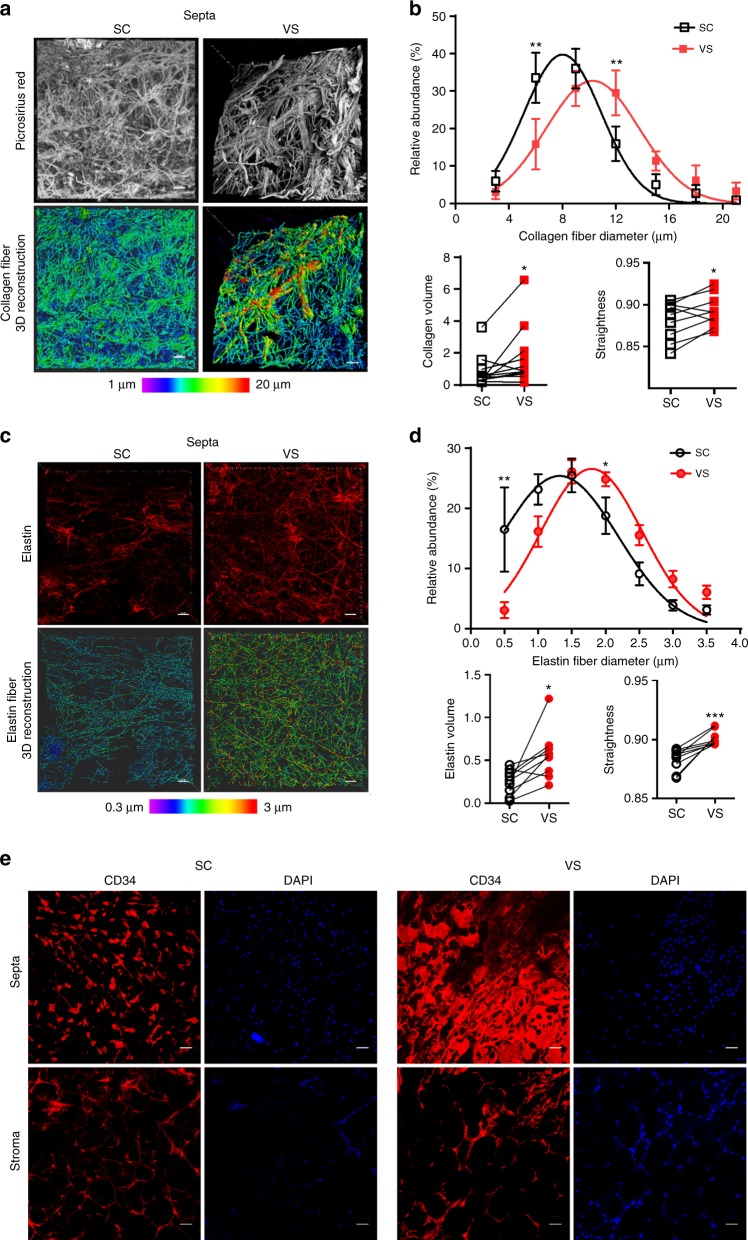
The extracellular matrix and CD34+ cells of SC and VSAT lobules. **a** Collagen fibers in paired subcutaneous (SC) and visceral (VS) lobule septa by picrosirius red labeling followed by three-dimensional reconstruction; the color code is set up according to the diameter of the fibers. Scale bars: 40 µm. **b** Distribution of the collagen fibers (percent) according to their diameter in SC and VS lobule septa (two-way ANOVA followed by Sidak’s multiple comparison test), the total volume of the collagen network (µm^3^ per image) and straightness of collagen fibers (paired *t* test). The results are means ± s.e.m. of independent experiments performed on *n* = 9 donors, *P < 0.05, **P < 0.01. **c** Elastin fibers in paired SC and VS lobule septa stained by an anti-elastin antibody followed by three-dimensional reconstruction; the color code is set up according to elastin fiber diameter. Scale bars: 40 µm. **d** Distribution (percent) of elastin fibers according to their diameter (two-way ANOVA followed by Sidak’s multiple comparison test), the total volume of elastin (µm^3^ per 100 µm^3^) and straightness of elastin fibers (paired *t* test) in SC and VS lobule septa. The results are means ± s.e.m. of independent experiments performed on *n* = 9 donors, *P < 0.05, **P < 0.01, and ***P < 0.001. **e** CD34+ cells in the septa and stroma of SC (left) and VS (right) lobules. Nuclei are stained with DAPI. Scale bars: 50 µm

We then analyzed the macro-architecture in paired subcutaneous (SC) and visceral (VS) AT in a cohort of obese individuals, candidate to bariatric surgery. Collagen staining with picrosirius red followed by three-dimensional reconstruction of lobule fibrous septa revealed clear fat depot-dependent fibrillar collagen content and organization (Fig. 5a). The VSAT lobule septa were characterized by a shift in the distribution in collagen fiber diameters toward largest and straighter fibers, as well as an enhanced total collagen volume (Fig. 5b). Elastin fiber content, assessed by immunostaining followed by three-dimensional reconstructions (Fig. 5c), showed a similar pattern, i.e., a higher proportion of larger elastin fibers, an increased total elastin volume and straightness in VSAT compared with SCAT (Fig. 5d). In addition, noticeable differences in CD34+ cell morphology and density were observed in the septa with an epithelial-like organization of the CD34+ cells in VSAT that was not observed in SCAT (Fig. 5e), while the CD34+ cell organization in the stroma did not display marked differences between AT location.

### VS progenitors exhibit mesothelial/epithelial-like phenotype

**Fig. 6:**
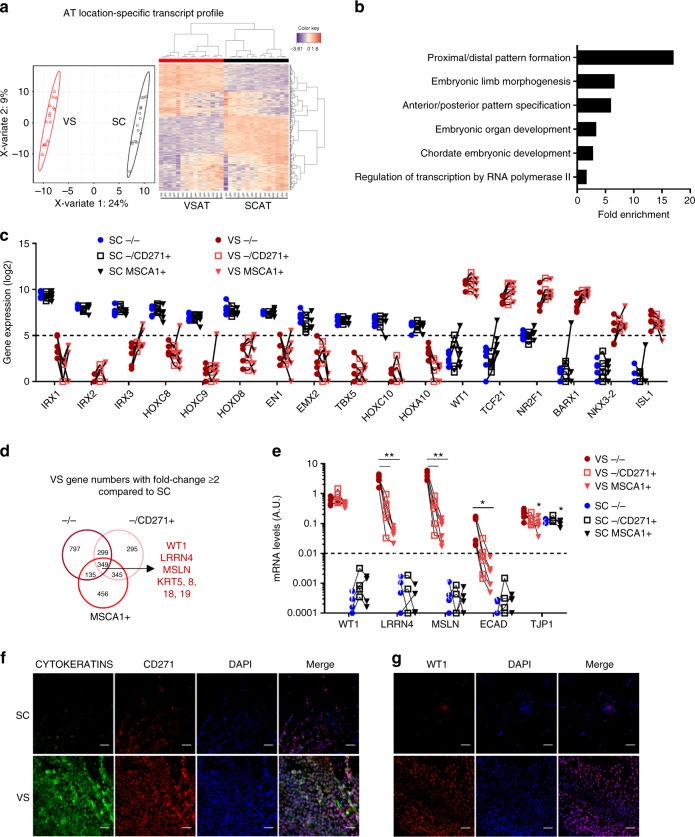
Mesothelial-like nature of VSAT progenitor subsets. RNAseq analysis was performed on paired progenitor subsets isolated from VS and SCAT of five obese donors. **a** Sparse partial least-square discrimant analysis plot of the two principal components. The 95% confidence ellipses are shown to strengthen the cell population clustering (left) and heatmap (right) of the top 300 most differentially expressed genes defining AT location-specific profile (visceral VS red, subcutaneous SC black). Color code is set up according to gene expression (high expression in red, low in blue). **b** Gene Ontology pathways obtained from 300 most differentially expressed genes defining AT location-specific profile independently of the progenitor subset. **c** Gene expression levels of SC and VSAT location-specific transcripts in progenitor subsets isolated from SC and VSAT determined from RNAseq analyses. The results are expressed as log2 gene expression, *n* = 5 independent donors. **d** Venn diagrams of differentially expressed genes (fold change ≥ 2 from a raw *P*-value) in each VS progenitor subset compared with SC subsets, highlighting some genes of interest. **e** mRNA levels of mesothelial markers (*WT1*, *LRRN4*, and *MSLN*) and epithelial markers (*ECAD*, *TJP1*) in native cell subsets sorted from VS (*n* = 6) and SC (*n* = 4) AT measured by RT-qPCR. Two-way ANOVA followed by Tukey’s multiple comparison test, *P < 0.05, **P < 0.01. **f** Representative images of CYTOKERATINS (green) and CD271 (red) immunostainings on the SC and VS lobule septa (*n* = 3). Nuclei are stained with DAPI (blue). Scale bars: 100 µm. **g** Representative images of WT1 (red) immunostainings on SC and VS lobule septa (*n* = 3). Nuclei are stained with DAPI (blue). Scale bars: 100 µm

We then further characterized the progenitor subsets according to their adipose depot location using RNAseq performed on matched native −/−, −/CD271+ , and MSCA1+ cells isolated from paired obese VSAT and SCAT. sPLS-D analyses discriminated AT location-specific transcript profiles independently of subsets (Fig. 6a), enriched in pathways related to development (Fig. 6b), highlighting transcription factors with marked fat depot-specific expression (Fig. 6c). Gene expression comparisons between VS and SC progenitor subsets (two or more fold changes) highlighted 349 common transcripts specifically upregulated in VS progenitor subsets, including mesothelial (*WT1*, *LRRN4*, and *MSLN*) and epithelial (*KRT5*, *8*, *18*, and *19*) -related transcripts (Fig. 6d; Supplementary Table 3) and pathways enriched in cell adhesion, epithelial cell differentiation, and cell development (Supplementary Fig. 3a). Among the 277 transcripts identified as upregulated in SC progenitor subsets, adipogenic-related genes *CD36*, *LPL*, and *PLIN1* (Supplementary Fig. 3b and Supplementary Table 3) were found, as well as pathways related to development and cell differentiation (Supplementary Fig. 3c). RT-qPCR performed on distinct donors confirmed a marked higher expression of mesothelial-related markers in VSAT compared with SCAT independently of the cell subsets (Fig. 6e). Indeed, levels of *WT1*, *LRRN4*, and *MSLN* were very low to undetectable in SCAT CD34+ cell subsets. Expression analysis of two classical epithelial markers showed that epithelial cadherin (*ECAD*) was very low to undetectable in SCAT CD34+ cells, while the zona occludens (*TJP1*) was similarly expressed in both tissue locations. To note, within VSAT, differences in mesothelial/epithelial transcripts were observed between progenitor subsets with −/− progenitors exhibiting the highest expression of *LRRN4*, *MSLN,* and *ECAD*, while intermediate levels were found in the −/CD271+ subset and the lowest in MSCA1+ cells (Fig. 6e). *WT1* was similarly expressed whatever the cell subset, whereas *TJP1* was expressed at lower levels in MSCA1+ cells (as observed in SCAT). To confirm the epithelial-like CD34+ organization in the septa, epithelial (cytokeratin), mesothelial (WT1), and CD271 immunolabeling were performed. The VSAT fibrous septa were characterized by higher cell density of cytokeratin as well as CD271-positive cells (double and single positive) compared with SCAT (Fig. 6f). WT1-positive staining was restricted to the nuclei in VSAT (Fig. 6f). These data suggest that the progenitor cells from VSAT exhibit a location-specific molecular signature hallmark of a mesothelial/epithelial origin.

### VS progenitor subsets share common potentials with SCAT

**Fig. 7:**
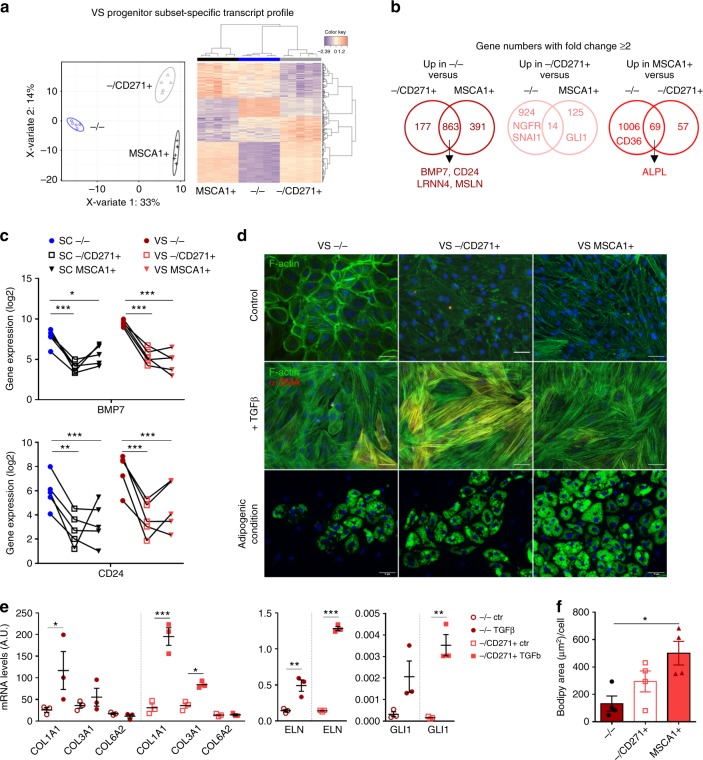
VS progenitors exhibit distinct differentiation potentials. RNAseq analysis was performed on paired progenitor subsets isolated from VS and SCAT of five donors. **a** Sparse partial least-square discrimant analysis plot of the two principal components. The 95% confidence ellipses are shown to strengthen the cell population clustering (left) and heatmap (right) of the top 400 most differentially expressed genes defining a progenitor subset-specific profile. Color code is set up according to gene expression (high expression in red, low in blue). **b** Venn diagrams of differentially expressed genes (fold change ≥ 2 from a raw *P*-value) between VS progenitor subsets: −/− cells vs. −/CD271+, and MSCA1+ (left), −/CD271+ cells vs. −/−, and MSCA1+ (middle) and MSCA1+ cells vs. −/− and −/CD271+ (right), highlighting some genes of interest. **c** Gene expression levels of *BMP7* and *CD24* in progenitor subsets isolated from SC and VSAT determined from RNAseq analyses. The results are expressed as log2 gene expression, *n* = 5 independent donors. Two-way ANOVA followed by Sidak’s multiple comparison test, *P < 0.05, **P < 0.01, ***P < 0.001. **d** Sorted VS progenitor subsets were cultured in the presence or absence of 5 ng/mL of TGFβ1 for 4 days and immunostaining approaches were performed using phalloidin (F-ACTIN, green), αSMA antibody (red), and DAPI (blue) or cultured under adipogenic condition and stained with Bodipy (green) and DAPI (blue). Representative fluorescence microscopy images are shown. Scale bars: 50 µm. **e** Gene expression levels of *COL1A1*, *COL3A1*, *COL6A2*, *ELN*, and *GLI1* in VSAT −/− and –/CD271+ progenitor subsets treated or not with TGFβ1 were determined by RT-qPCR analyses. The results are expressed as means ± s.e.m. of experiments performed on *n* = 3 independent donors. One-way ANOVA followed by Sidak’s multiple comparison test, *P < 0.05, **P < 0.01, ***P < 0.001. **f** Quantification of lipid accumulation in VS progenitor subsets at day 10, results are means ± s.e.m. of experiments performed on *n* = 4 independent donors. One-way ANOVA followed by Tukey’s multiple comparison test, *P < 0.05

sPLS-D analyses discriminated VSAT progenitor subset-specific transcript profiles with clustering of −/− cells with −/CD271+ cells followed by MSCA1+ cell subsets (Fig. 7a). Gene expression comparisons between VSAT progenitor subsets (two or more fold changes) highlighted 863 transcripts specifically upregulated in −/− subset, 14 in −/CD271+ subset, and 69 in MSCA1+ subset (Fig. 7b; Supplementary Table 4). *BMP7* and *CD24*, already identified as SCAT −/− specific transcripts, were also upregulated in VSAT −/− subset (Fig. 7c), together with the mesothelial markers *LRRN4* and *MSLN*. As expected, *NGFR* (encoding for CD271) as well as *GLI1* were upregulated in −/CD271+ subset, while *ALPL* (encoding for MSCA1) and *CD36* were upregulated in MSCA1+ subset (Fig. 7b). sPLS-D analyses discriminated progenitor subset-specific transcript profiles independently of AT location (Supplementary Fig. 3d). SC and VSAT −/− progenitor subset exhibited higher gene expression of transcripts enriched in cell migration, adhesion, and differentiation, −/CD271+ subset in cell-surface receptor signaling and development, and MSCA1+ subset in metabolic processes (Supplementary Fig. 3e). Therefore, VSAT progenitor subsets, despite a distinct origin, share common transcripts with SCAT subsets. To determine whether such signatures are associated with distinct differentiation potentials, immunoselected VSAT progenitors were studied in vitro. VSAT −/− progenitor subset exhibited an epithelial-like organization characterized by a marked flattened cell morphology and F-ACTIN bound to the plasma membrane. VSAT −/CD271+ cells exhibited an intermediate phenotype, while VSAT MSCA1+ cells were all spindle-shaped cells (Fig. 7d). In the presence of TGFβ1 for 4 days, all VSAT subsets exhibited changes in F-ACTIN fiber organization, but the emergence of αSMA-positive cells was mainly observed in the −/CD271+ population (Fig. 7d). To note, some αSMA-positive cells were also found in VSAT −/− population and to a lower extent in MSCA1+ population. The detection of αSMA-positive cells in the −/CD271+ population treated with TGFβ1 was accompanied by a higher induction of *COL1A1*, *COL3A1*, *ELN*, and *GLI1* compared with −/− progenitor cells (Fig. 7e). Conversely, adipogenic potential assessed by culturing the cell subsets under adipogenic condition for 10 days was markedly higher for VSAT MSCA1+ cells compared with other CD34+ cell subsets, as shown by Bodipy staining and lipid accumulation quantification (Fig. 7d–f).

### SCAT has higher remodeling capacities than VSAT

**Fig. 8:**
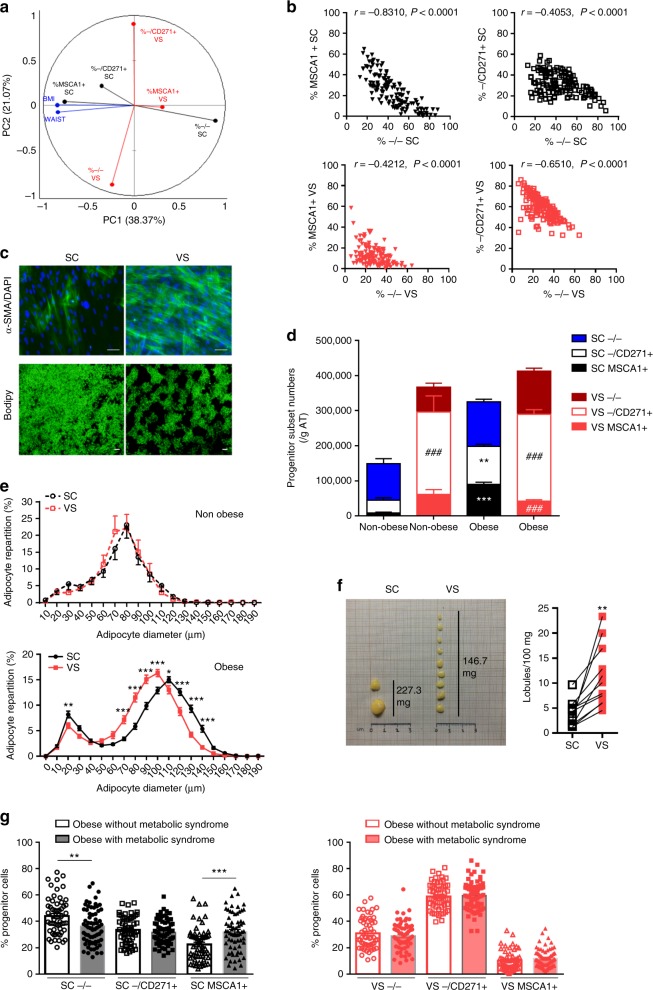
Intrinsic differences in progenitor cell fate in SC and VSAT. **a** Principal component analysis performed on the percentage of progenitor cell subsets (determined by flow cytometry) from paired biopsies of subcutaneous (SC, black) and visceral (VS, red) AT according to BMI and waist circumference of *n* = 23 non-obese and *n* = 123 obese women. **b** Spearman correlation between the percentage of −/− cells and MSCA1+ cells or −/CD271+ cells in SCAT (black) and VSAT (red) (*n* = 23 non-obese and *n* = 123 obese women). **c** Myofibroblastic and adipogenic potential of obese SC and VSAT progenitor cells assessed by αSMA/DAPI staining and Bodipy staining, respectively. Representative immunofluorescence analyses performed on three independent donors. Scale bar: 100 µm. **d** Progenitor cell number per gram of AT in non-obese (*n* = 23) and obese (*n* = 123) SC and VSAT, the results are means ± s.e.m. Two-way ANOVA followed by Tukey’s multiple comparison test, **P < 0.01 and ***P < 0.001 comparing non-obese and obese subsets in the same AT location, ^###^*P* < 0.001 comparing SC and VS subsets of non-obese or obese individuals. **e** Distribution of mature adipocytes according to diameter in SC and VS lobules in *n* = 11 non-obese (upper panel) and *n* = 71 obese (lower panel) individuals, the results are means ± s.e.m. Two-way ANOVA followed by Sidak’s multiple comparison test, **P < 0.01. **f** Representative image of dissected lobules of matched SC and VSAT from obese donors and number of lobules per 100 mg of paired SC and VSAT, paired *t* test, **P < 0.01 (*n* = 11 independent donors). **g** Progenitor cell subset repartition in SC (left, black) and VS (right, red) AT from obese patients with (white bars, *n* = 57) or without (filled bars, *n* = 66) a metabolic syndrome, the results are means ± s.e.m. One-way ANOVA followed by Sidak’s multiple comparison test, **P < 0.01, ***P < 0.001

Flow cytometry analyses were performed on SC and VSAT from non-obese and obese women (Supplementary Fig. 4). Principal component analyses highlighted as main contributors of component 1 BMI and waist circumference associated with SCAT MSCA1+ subset that was anti-correlated with SCAT −/− subset and as a main contributor of component 2, the VSAT −/CD271+ subset that was anti-correlated with the VSAT −/− subset (Fig. 8a). Indeed, the proportion of −/− progenitor cells was inversely correlated with the proportion of MSCA1+ subset in SCAT, while it was inversely correlated with −/CD271+ myofibroblast precursors in VSAT (Fig. 8b), suggesting that with obesity, the progenitor cells were preferentially oriented toward adipogenesis in SCAT and myofibroblast in VSAT. In agreement, the whole CD34+ progenitors exhibited an enhanced adipogenic potential (Bodipy) in SCAT, while a myofibroblastic one (αSMA) in VSAT in obese patients (Fig. 8c). To further analyze AT remodeling with obesity, numbers of progenitor subsets and changes in adipocyte diameters were analyzed. Non-obese women exhibited a marked AT location-specific subset signature with −/− cells as the main progenitor subset in SCAT and −/CD271+ subset in VSAT. In obese women, the numbers of MSCA1+ and −/CD271+ subsets increased in SCAT, while no modification was observed in VSAT, leading to marked differences between AT location with less MSCA1+ and more −/CD271+ cell numbers in VSAT than SCAT (Fig. 8d). The repartition of adipocyte diameter in both tissues was similar in non-obese women, while in obese women, SCAT showed more hypertrophied adipocytes, together with a higher proportion of smaller adipocytes than VSAT (Fig. 8e). The highest SCAT remodeling capacity in terms of progenitor and adipocyte populations was associated with bigger lobules compared with VSAT in obese women (Fig. 8f). Finally, progenitor subsets were analyzed according to the presence or absence of a metabolic syndrome with obesity. As shown in Fig. 8g, obese women with a metabolic syndrome exhibited less −/− and more MSCA1+ cell subsets in SCAT compared with obese women without a metabolic syndrome, while no changes were observed in VSAT, further highlighting a limited expandability of VSAT according to physiopathological changes.

## Discussion

The interrelations between mature adipocytes and the microenvironment are multiple, but mainly explored at the level of the stroma. In this study, we highlight another level by characterizing the organization unit of human adipose tissue (hAT), i.e., the lobule composed of two compartments of extracellular matrix and two niches of progenitor cells delineating fibrous septa and stroma.

Since their first description in hAT, AT mesenchymal stem/progenitor cells gained great interest for regenerative medicine on one hand and in metabolism pathophysiology field on the other hand. Among the native pool of AT progenitor cells, distinct cell subsets have been identified based on cell-surface markers, including MSCA1 and CD36, which identify the committed adipogenic progenitor cells^11,12^. In this study, we provide evidence that the cell-surface expression of CD271 together with lack of MSCA1 expression identifies the progenitor cell subset with myofibroblastic potential. Indeed in basal condition, human native −/CD271+ cells express higher levels of the myofibroblast precursor marker GLI1^24^. Under TGFβ1 stimulation, the CD271+ cells differentiate into αSMA-positive cells expressing higher levels of the extracellular fibrillar matrix proteins. In the liver, the expression of both CD271 and its main ligand NGF, positively correlates with fibrosis^25^, and the expression of CD271 is required for myofibroblast differentiation of hepatic stellate cells^26^. In agreement, we demonstrate in hAT that the myofibroblast differentiation requires the activation of the NGF/CD271 axis. Therefore, the native pool of progenitor cells in human adult AT is composed of subsets with distinct fates. The niche of progenitor cells in situ is now well recognized as a major determinant of progenitor cell fate commitment. In this study, we show that the distribution of the distinct progenitor subsets is spatially organized. Adipogenic committed MSCA1+ cells are enriched in the lobule stroma. The MSCA1+ cell subset is markedly increased with obesity in SCAT, and the adipogenic potential of whole CD34+ progenitor cells is markedly higher in SCAT than VSAT from obese women. Therefore, the intrinsic adipogenic capacity of the SCAT is higher than the one of VSAT. Accordingly, we report a higher percentage of smaller adipocytes in SCAT than VSAT in obese women. The myofibroblastic precursor −/CD271+ cells are found in both septa and stroma, but exhibit a higher CD271 expression when localized in the septa. The septa is mainly composed of a loose fibrillar network consisting of COLLAGEN 1, 3, and ELASTIN. These components are expressed at higher mRNA levels in the activated committed myofibroblast, suggesting involvement of −/CD271+ cells in the production of fibrous septa itself. We reveal a marked denser and thicker septa associated with a higher number of −/CD271+ cells in VSAT compared with SCAT, whatever the obesity state. Taken together, the results strongly suggest that the myofibroblastic intrinsic capacity is higher in VSAT than SCAT. In agreement, whole VSAT progenitors exhibit a marked higher myofibroblastic potential than SCAT. Since the lobule size is smaller in VSAT as well as the proportion of hypertrophied adipocytes, it is tempting to speculate that the physical constraints associated with dense fibrous septa in VSAT restrain the expansion of lobules and adipocyte hypertrophy, and therefore the whole VSAT expandability. To note, CD9-positive progenitor cells have been described to play a role in VSAT fibrosis^14^. We observe that all CD34+ cell subsets, including −/CD271+ cells, do express CD9. Taken together, these results show that both SCAT and VSAT exhibit marked intrinsic differences in adipogenic and myofibroblastic capacities in relation with the stroma and septa niches. Moreover, SCAT exhibits a higher remodeling capacity in the pathophysiological context, including obesity and metabolic syndrome. The origin of such differences may rely on the nature of the progenitor cells. Various developmental origins of adipocytes have been suspected, based on the differential developmental gene signatures detected across AT locations^20^. We observed adipose depot-specific expression of transcription factors involved in body patterning for SCAT (*HOX*, *IRX*, *TBX5*, and *EN1*) and in mesenchymal and epithelial lineages for VSAT (*WT1*, *TCF21*, *BARX1*, and *NKX3-2*). Among the SCAT- and VSAT-specific transcription factors, *HOXC8*, *HOXC9*, *IRX3*, *TBX5*, and *ISL1* have been described as active players in modulating white/brite adipogenesis^27,8^, while others, including *WT1*, *NR2F1*, and *TCF21*, as molecular signatures of visceral adipocyte lineage^27^. In addition, we demonstrate that although the niche is common in both depots location, i.e., lobule septa, the nature of the −/− progenitor subset is different and in favor of a mesothelial-like origin based on the expression of lineage-specific markers and cell organization and morphology. Mesothelial cells lay on the collagen and elastin network that cover internal organs^28^, are positive for CD34, and exhibit a mixed epithelial and mesenchymal phenotype^18^. We demonstrate that the native VSAT −/− cells express mesothelial markers, including *WT1*, mesothelin, and *LRRN4*, as well as epithelial markers *TJP1*, E-Cadherin, and cytokeratins. Both VSAT −/CD271+ and MSCA1+ cell subsets still express mesothelial and epithelial markers, but at lower levels. The decreased expression of mesothelial and epithelial markers in −/CD271+ cells and MSCA1+ cells from VSAT may be related to a switch from mesothelial to mesenchymal phenotype^28^. *SNAI1*, involved in induction of the epithelial to mesenchymal transition, is a marker of −/CD271+ cell subset in agreement with our previous publication, showing increased *SNAI1* expression in human AT whole progenitor cells in response to TGFβ1^13^. Furthermore, the cell-tracking experiments performed in mice showed that visceral and epicardial adipocytes may arise from mesothelial cells that were positive for WT1^18,29^ and mammary adipocytes from secretory epithelial cells in the mammary gland^30^. Interestingly, Hepler et al. recently identified three PDGFRβ+ subpopulations in mouse visceral AT with reminiscent signatures, including mesothelial-like, committed preadipocyte and fibro-inflammatory progenitor subpopulations^31^. To note, the human VSAT −/−, −/CD271+, and MSCA1+ subsets expressed similar levels of PDGFRβ (RNAseq data, the average of log2 values are 11.48, 12.52, and 12.46 for −/−, −/CD271+, and MSCA1+ cells, respectively). Whether connections exist between the mesothelium surrounding the omentum and lobule fibrous septa inside the tissue will be worthwhile to determine. In human subcutaneous layers, fibrous septa around fat lobules connect the superficial fascia to the skin and to the deep fascia that covers skeletal muscle bundles. Cells residing in the superficial fascia from the rat hindlimb exhibit adipogenic capacity^32^. Further approaches will be required to clearly define the nature of the fascia cells and their potential link with the SCAT progenitors. In conclusion, our study proposes to unify both portal hypothesis and the expandability concept by taking into account the progenitor cell niche, nature, and fate in human fat depots. The expandability of the SCAT may be favored through the pro-adipogenic potential of its stroma, promoting adipocyte renewal and hyperplasia. In addition, the loose composition of its septa allows stromal adipocyte hypertrophy. On the contrary, the expandability of VSAT may be restricted through the myofibroblastic potential of its dense fibrous septa, leading to physical constraints limiting stromal adipocyte hypertrophy. Also, the poor adipogenic potential of VSAT may restrict adipocyte hyperplasia and renewal of mature adipocytes, leading to the accumulation of dysfunctional mature adipocytes. It would be interesting to determine whether such differences may be related to the mesothelial/epithelial-like phenotype of VSAT progenitor cells. Our approaches do not permit to conclude whether the subsets that we identified according to their cell-surface markers, niche and fate, are hierarchically related. Moreover, we did not consider potential dynamics as well as interactions between the three subsets. Our study sheds light on the AT lobule organization and opens new avenues for further investigations and deeper characterization of human fat lobule heterogeneity and physiology.

## Methods

### Human cohorts and tissue collection

Subcutaneous human ATs were obtained from healthy adult women undergoing dermolipectomy. The protocol was approved by Ministère de la Recherche, direction générale de la recherche et de l’innovation, cellule bioéthique (DC2008-452). Subcutaneous and visceral AT-matched biopsies were obtained from obese patients undergoing gastric bypass surgery and from non-obese women undergoing gynecological surgery. The protocol is registered at ClinicalTrials.gov (SENADIP: NCT01525472), and was approved by Comité de Protection des Personnes I.D.F.VII. All donors gave their informed consent. Women with flow cytometry analyses performed on progenitor cells were taken into account in this study. Characteristics are indicated in Supplementary Table 1.

### Cell isolation and culture

Adipose tissue lobules (as whole or dissected under a binocular lens to obtain the lobule fibrous septa and the stroma, Fig. 2a) were either fixed with 4% paraformaldehyde (PFA) solution at room temperature to perform three-dimensional microscopy analyses or digested using type I collagenase (Sigma-Aldrich). Whole AT was sequentially digested with 1:1 dispase (2.4 U/mL in phosphate-buffered saline (PBS), Gibco, 30 min at 37 °C with shaking) and type I collagenase (250 U/mL in PBS 2% bovine serum albumin (BSA), Sigma-Aldrich, 30 min at 37 °C with shaking). After digestion, the cell suspension was filtered through a 250 -µm strainer and centrifuged. Erythrocyte lysis step was performed followed by successive filtrations through 100, 70, and 40 -µm strainers. The viable recovered cells were counted and further analyzed by flow cytometry and RT-qPCR, or used to isolate the cell subsets either by magnetic bead-based selection or cell sorter. Immune cells and endothelial cells were removed using CD45 depletion kit (Stem Cells Technologies) and CD31 microbeads kit (Dynal, Thermofisher), respectively, following each manufacturer’s protocol. The purity of CD45–/CD34+/CD31− cell fraction was assessed by flow cytometry. The progenitor cell subsets, i.e., MSCA1+, −/CD271+ (MSCA1−/CD271+), and −/− (MSCA1−/CD271−) were isolated using a cell-sorting approach. In total, 15.10^6^ SVF cells were incubated with fluorescent-labeled antibodies anti-human: V450-CD31 clone WM59 1:20 (BD Biosciences), FITC-CD34 clone AC136 1:20, PE-MSCA1 clone W8B2 1:20, APC-CD271 clone ME20.4-1.H4 1:20, and PE-Vio770-CD14 clone TÜK4 1:100 (Miltenyi Biotec) for 30 min at 4 °C in PBS 0.5% BSA 2 mmol/L EDTA. Cells were washed with PBS and sorted with BD Influx^TM^ cell sorter (BD Biosciences, Sortware software) (pressure 20 psi, nozzle 100 µm) according to the gating strategy, shown in Supplementary Fig. 5. The average purity of sorted −/− subset was 96%, of −/CD271+ subset was 93%, and of the MSCA1+ subset was 97%. Cells were seeded at 120,000 cells/cm² for a 2 day-recovery period in ECGM-MV (Promocell), and treated once for 4 days in ECBM (Promocell) containing 0.1% BSA in the presence or absence of human recombinant TGFβ1 5 ng/mL (Peprotech), 25 µmol/L γ-secretase inhibitor (CAS 200810-93-1, Sigma-Aldrich, diluted in 0.25% dimethylsulfoxyde), or 5 ng/mL neutralizing antibody against human nerve growth factor (NGF, Abcam) or control antibody. For adipogenesis, isolated progenitor cell subsets were seeded at 120,000 cells/cm² in ECGM-MV 100 units/ml penicillin and 100 µg/ml streptomycin, for 2 days. Then, the medium was changed to ECBM containing 100 units/ml penicillin, 100 µg/ml streptomycin, 66 nmol/L insulin, 1 nmol/L triiodothyronine, 0.1 µg/ml transferrin, and 100 nmol/L cortisol (ITTC) supplemented with 3 µM rosiglitazone. After 3 days, the medium was replaced with ITTC (without rosiglitazone) and refreshed every 2–3 days for the following 7 days.

### Flow cytometry analyses

In total, 100,000 cells were incubated with fluorescent-labeled antibodies for cell-surface markers (anti-human V500-CD45 clone HI30 1:20, PerCP-CD34 clone 8G12 1:20, FITC-CD9 clone M-L13 1:5, and V450-CD31 clone WM59 1:20 (BD Biosciences), PE-MSCA1 clone W8B2 1:10 and APC-CD271 clone ME20.4-1.H4 1:10 (Miltenyi Biotec), APC-Cy7 CD36 clone 5-271 1:100 (BioLegend), or an appropriate isotype control) for 30 min at 4 °C in PBS supplemented with 0.5% BSA and 2 mmol/L EDTA. Cells were washed with PBS and analyzed using a FACS Canto^TM^ II flow cytometer and Diva Pro software (BD Biosciences). Fluorescence-minus-one (FMO) controls were performed and used to control the gating strategy (Supplementary Fig. 1).

### Transcriptional analysis

The total RNA was isolated from cells using RNeasy mini KIT (Qiagen). cDNA synthesis was performed on 250 ng (or 150 ng for cells harvested from the lobule septa and stroma) of the total RNAs with Superscript II (Thermofisher) and random hexamer primers. Relative gene expression levels were assessed using Taqman^®^ Probes and Applied mastermix (Thermofisher) on a ViiA7 system (Applied Biosystems): *ACTA2* (αSMA) Hs00909449-m1; *ALPL* (MSCA1) Hs01029144-m1; *CD9* Hs00233521-m1; *CD36* Hs0016927-m1; *CD271* (NGFR) Hs00182120-m1; *CDH1* (E-cadherin) Hs01023895-m1; *COL1A1* Hs00164004-m1; *COL3A1* Hs00943809-m1; *COL6A1* Hs01095585-m1; *COL6A2* Hs00242484-m1; *COL6A3* Hs00915120-m1; *ELN* (Elastin) Hs00355783-m1; FAP Hs00990806-m1; *GLI1* Hs01110766-m; *INHBA* Hs00170103-m1, *LRRN4* Hs00379905-m1; *MSLN* (Mesothelin) Hs00245879-m1; *NGF* Hs00171458-m1; *PPARG2* Hs01115510-m1; *TJP1* Hs01551861-m1; *WT1* Hs01103751-m1. Each of the samples were run in duplicate, and the relative amount was normalized to *18* *S* housekeeping gene. Data were analyzed using ViiA7 software.

### RNASeq analysis

RNA was isolated from sorted progenitor cells (suspended in RNAprotect cell reagent), obtained from paired SC and VSAT of five distinct donors, using RNeasy protect cell mini kit (Qiagen). RNA quality was measured using Agilent RNA 6000 Pico Kit (Agilent) and Bioanalyzer instrument with eukaryote total RNA Pico protocol (Agilent) and was RIN ≥ 7. RNA samples were quantified using Qubit™ RNA HS Assay Kit (Invitrogen) and Qubit 2 Fluorometer (Invitrogen). About 10 ng of RNA was used for AmpliSeq transcriptome library construction. For AmpliSeq transcriptome sequencing, library construction AmpliSeq™ Library PLUS, AmpliSeq Transcriptome Human Gene Expression Panel, and AmpliSeq CD indexes SetA kits were purchased from Illumina, and sequencing libraries were constructed as described in AmpliSeq for Illumina Transcriptome Human Gene Expression Panel reference guide (Illumina). Sequencing libraries were quantified using a Qubit dsDNA HS Assay Kit and Fluorometer (Invitrogen) and converted into molar concentration using a 285-bp peak. Equimolar concentrations of libraries were pooled at 4 nM and used for sequencing. A pooled library was denatured and diluted, as described in Denature and Dilute Libraries Guide (Illumina), and adjusted to a final concentration of 1.4 pM. The resulting library was sequenced on NextSeq 500 using NextSeq 500/550 High Output v2 kit with 2 × 151-bp cycle. Generated raw files were converted into FASTQ files and used for data analysis. AmpliSeq transcriptome FASTQ files were analyzed on Array studio V10.0 (Omicsoft, Qiagen). Following raw read QC, the first and last ten bases were trimmed and mapped to the reference genome Human.B38. The read count data were generated using GeneModel RefGene20170606. The resulting data were normalized by the DESeq package, transformed to a log2 value, and used for ANOVA analyses, or log2-normalized expression values were subjected to paired sparse partial least-squares discriminant analyses (sPLS-DA from the mixOmics R package). We selected 300 genes based on subcutaneous versus visceral location (2 components, 150 genes per component) using all cellular subsets and similarly selected 400 genes (2 × 200) using the cellular subset as a discrete outcome, using both locations combined as well as independently. Heatmaps were plotted with the cim function from mixOmics. Superpathways were analyzed using GeneAnalytics geneanalytics.genecards.org (Gene Cards Suite) and Gene Ontology pathways using Gene Ontology Resource (Panther analysis, GO Ontology database released 2019-01-01).

### Western blot analysis

Cells were lysed in RIPA buffer supplemented with anti-protease and anti-phosphatase. In total, 10–30 µg of protein lysates were resolved using the following primary antibodies: polyclonal rabbit anti-human CD271 (Santa Cruz Biotec NGFR p75 (H-92) sc-5634, 1:500), polyclonal rabbit anti-human GLI1 (ThermoFisher PA5-17303, 1:1000), and monoclonal mouse anti-human αSMA (DAKO clone1A4, 1:1000). Detection was performed with an appropriate HRP-coupled secondary antibody and West Dura reagent (ThermoFisher). Glyceraldehyde 3-phosphate deshydrogenase (GAPDH) (Cell Signaling monoclonal rabbit anti-human GAPDH, clone 14C10, 1:1000) was used as a loading control. Densitometry was quantified using Chemidoc detection system (Bio-Rad Laboratories). Full-length western blot images presented in Fig. 4 are provided in Supplementary Fig. 2.

### Immunofluorescence staining

Cells or AT were fixed with 4% PFA at room temperature for 10 min for cells and up to 1 h for AT. Samples were blocked using PBS, 3% BSA supplemented or not with 0.2% Triton X-100 for 30 min (cells) to 60 min (AT). Then, samples were incubated for 1 h at room temperature for cells and overnight at 4 °C for AT with primary antibodies (rabbit polyclonal anti-Type I Collagen (Novus NB600-408, 1:100); rabbit polyclonal anti-Type III Collagen (Santa Cruz Biotec (H-300) sc28888, 1:100); mouse monoclonal anti-Elastin (Millipore, clone 10B8, 1:200); rabbit polyclonal anti-CD271 (Cusa BIO CSB-PA003448, 1:100); rabbit monoclonal anti-CD34 (Epitomics clone EP373Y, 1:100); mouse polyclonal anti-pan Cytokeratin (Abcam, C-11 ab7753 from tissue culture supernatant, 1:25); rabbit monoclonal anti-WT1 (Abcam clone CAN-R9(IHC)-56-2, 1:50)). Then samples were incubated with appropriate Alexa Fluor-conjugated secondary antibodies (Invitrogen). F-ACTIN is stained with AF88-conjugated PHALLOIDIN (ThermoFisher, 1:100). Nuclei were stained with DAPI (0.5 µg/ml, Invitrogen) and fat accumulation was visualized with Bodipy 493/503 (10 µg/ml, Invitrogen). Images were taken with an inverted fluorescent microscope (Nikon Eclipse TE300, software NIS-Elements 2.5 BR software V4.10, Nikon^®^) or with a confocal microscope (ZEISS LSM780, ZEN software). Three-dimensional reconstructions and analyses were performed with ImageJ or Imaris (Bitplane) softwares. To quantify the Bodipy area per cell, images were taken with a ×10 objective. The Bodipy area (µm²) was determined using ImageJ software and normalized to the cell number counted from the DAPI staining. The results are the mean of 3–5 images for each sample.

### Transmission electron microscopy

Samples were fixed with 2% glutaraldehyde in Sorensen buffer (0.1 mol/L, pH = 7.4) for 1 h, washed with the Sorensen phosphate buffer (0.2 mol/L) for 12 h, and postfixed with 1% OsO_4_ in Sorensen buffer (Sorensen phosphate, 0.05 mol/L; glucose, 0.25 mol/L; OsO_4_, 1%) for 1 h. Samples were dehydrated in increasing ethanol series (up to 100% ethanol) and then with propylene oxide. Samples were embedded in epoxy resin (Epon 812). After 48 h of polymerization at 60 °C, ultrathin sections (70 nm) were mounted on 100-mesh collodion-coated copper grids and poststained with 3% uranyl acetate in 50% ethanol and with 8.5% lead citrate, before being examined on an HT7700 Hitachi electron microscope at an accelerating voltage 80 kV.

### Collagen staining

Fixed AT lobules were incubated for 3 h in PicroSirius Red solution (1 g/L Sirius red in 1.3% picric acid). After two washes in acidified water followed by fixation/dehydration steps in 100% ethanol, images were taken using a confocal microscope with 560 -nm excitation and above 600 -nm emission (ZEISS LSM780, ZEN software). Three-dimensional reconstructions and analyses were performed with IMARIS software.

### Statistical analyses

Statistical analyses were performed using Prism (GraphPad Software). Comparisons between two groups were analyzed either by two-tailed paired or unpaired Student’s *t* test with a 95% confidence interval. Comparisons between more than two groups were analyzed by one-way or two-way ANOVA, followed by appropriate post tests for (*n*) independent experiments. Correlations were obtained using Spearman test. Differences were considered statistically significant when *p* < 0.05.

### Reporting summary

Further information on research design is available in the Nature Research Reporting Summary linked to this article.

## Supplementary information

Supplementary Information

Reporting Summary

## Data Availability

All RNAseq datasets generated and used in this study are available from the NCBI Gene Expression Omnibus (GEO) portal, with the following GEO accession number GSE127222.
