# Supplementary material for: Lobular architecture of human adipose tissue defines the niche and fate of progenitor cells
Source: Nat Commun. 2019 Jun 11;10:2549. doi: 10.1038/s41467-019-09992-3 (PMC6560121; doi:10.1038/s41467-019-09992-3)
Supplement: Supplementary file 1 — Supplementary Information [file 41467_2019_9992_MOESM1_ESM.pdf]

Supplementary Information

Lobular architecture of human adipose tissue defines the niche and fate of progenitor cells

Estève D & Boulet N et al.

Correspondence: jean.galitzky@inserm.fr

Supplementary Information includes 5 supplementary figures and 4 supplementary tables.

Supplementary Figure 1

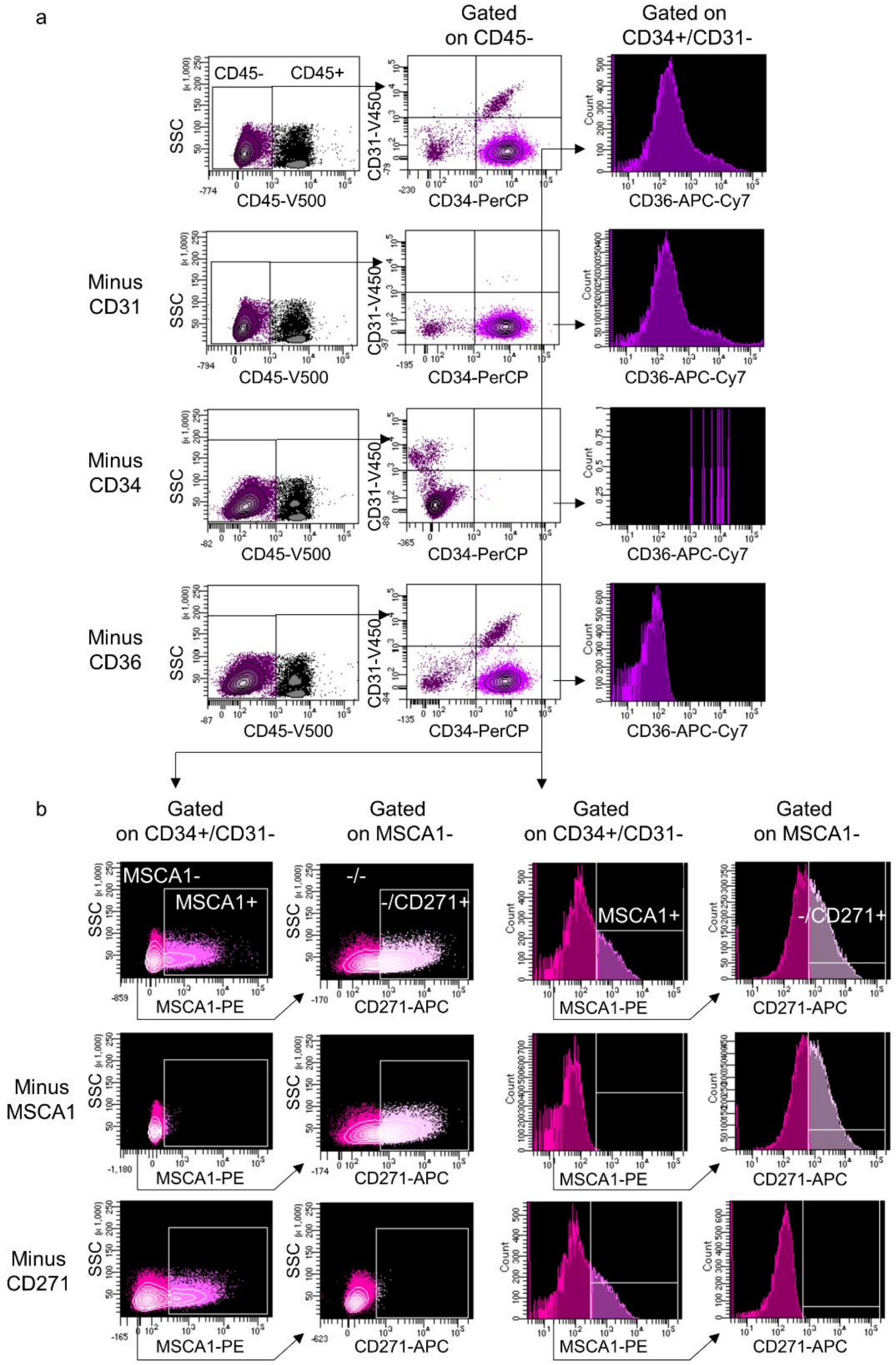

Supplementary Figure 1: Fluorescence minus one controls

Freshly isolated SVF cells were analyzed by flow cytometry using anti-CD45, -CD31, -CD34, -CD36, -CD271 and -MSCA1 antibodies. a) Representative dot-plots of CD45 / CD34 / CD31 stainings and histograms of CD36 in the CD45-/CD31-/CD34+ progenitor population are shown with and without one indicated antibody. b) Representative dot-plots and histograms of MSCA1 staining gated in the CD45-/CD31-/CD34+ progenitor population and of CD271 staining gated in the MSCA1- population are shown with and without one indicated antibody. The experiment was performed on 4 distinct donors.

Supplementary Figure 2

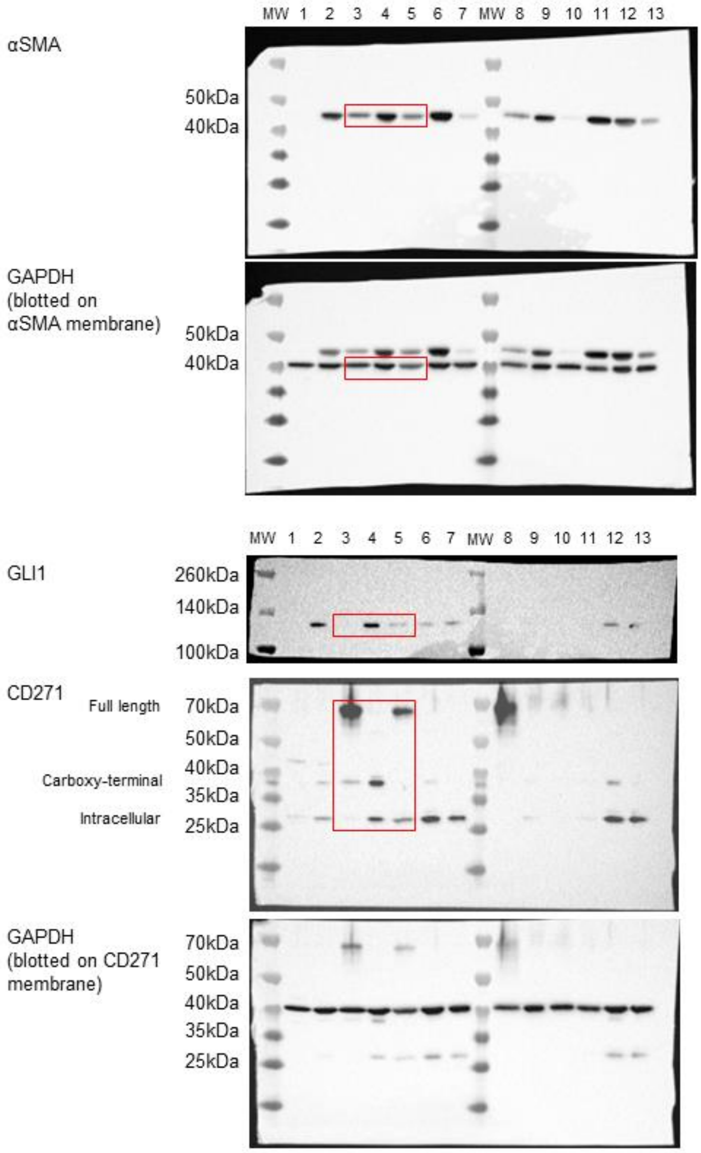

1: donor 1 Control; 2: donor 1 TGFβ; 3: donor 2 Control; 4: donor 2 TGFβ; 5: donor 2 TGFβ + γSI; 6: donor 3 TGFβ; 7: donor 3 TGFβ + γSI; 8: donor 4 Control; 9: donor 4 TGFβ; 10: donor 4 TGFβ + γSI; 11: donor 5 Control; 12: donor 5 TGFβ; 13: donor 5 TGFβ + γSI

Supplementary Figure 2: Full-length western blot images

Supplementary Figure 3

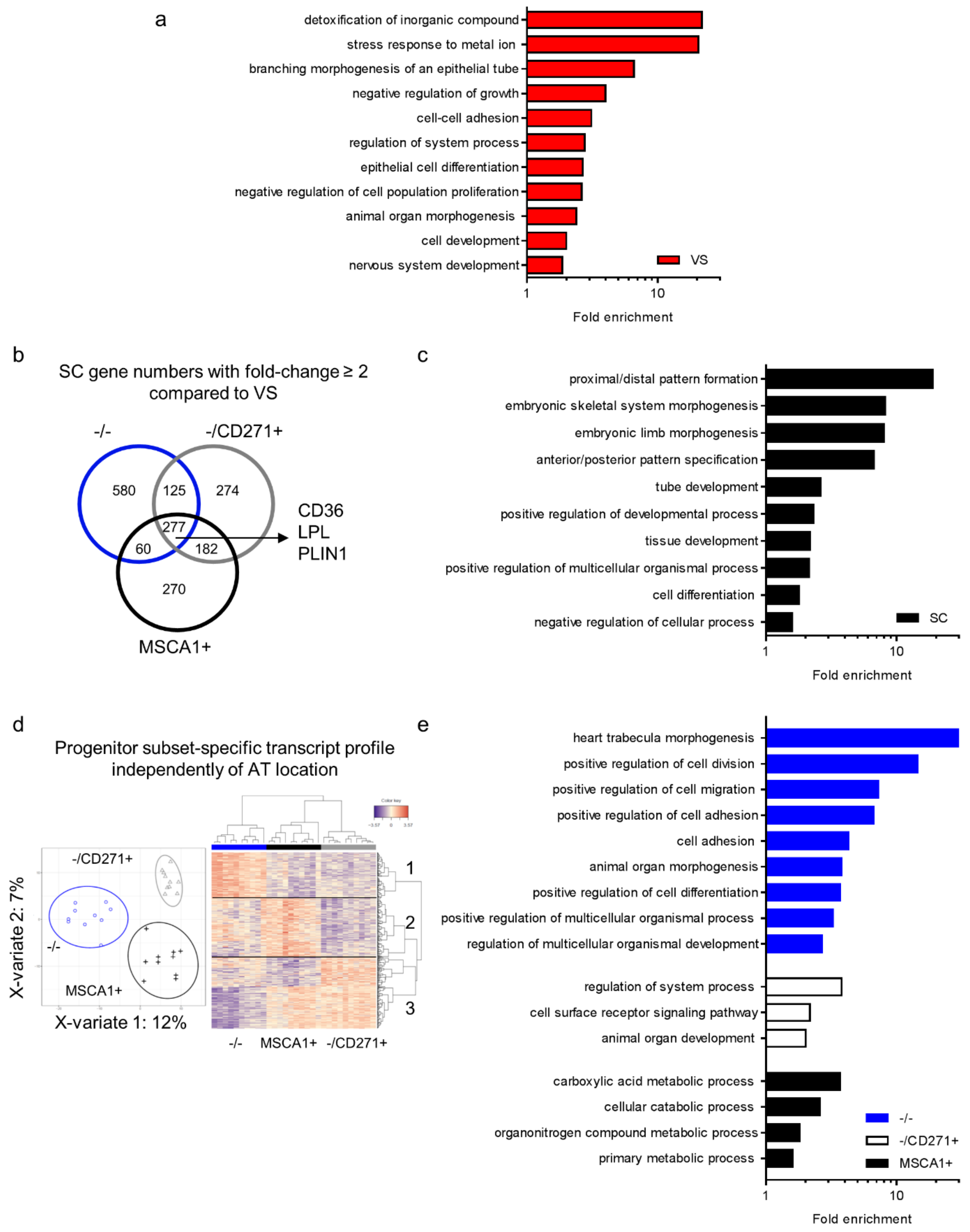

Supplementary Figure 3:

a) GO pathway analysis of VSAT location-specific genes commonly expressed by all the progenitor subsets (349 genes from Fig. 6). b) Venn diagrams of differentially expressed genes (2 and more fold-change) in each SC progenitor subset compared to VS subsets, highlighting some genes of interest. c)

GO pathway analysis of SCAT location-specific genes commonly expressed by all the progenitor subsets (277 genes). VS and SC transcripts are listed in Supplementary Table 3. d) Sparse partial least square discrimant analysis plot of the two principal components. The 95% confidence ellipses are shown to strengthen the cell population clustering (left) and heatmap (right) of the top 300 most differentially expressed genes defining progenitor subset-specific profile independently on SC and VS AT location. Color code is setup according to gene expression (high expression in red, low in blue). e) GO pathway analysis of progenitor subset-specific genes independently on AT location, -/- obtained from gene cluster 1, -/CD271+ from gene cluster 3 and MSCA1+ from gene cluster 2 (clusters are indicated on the heatmap).

Supplementary Figure 4

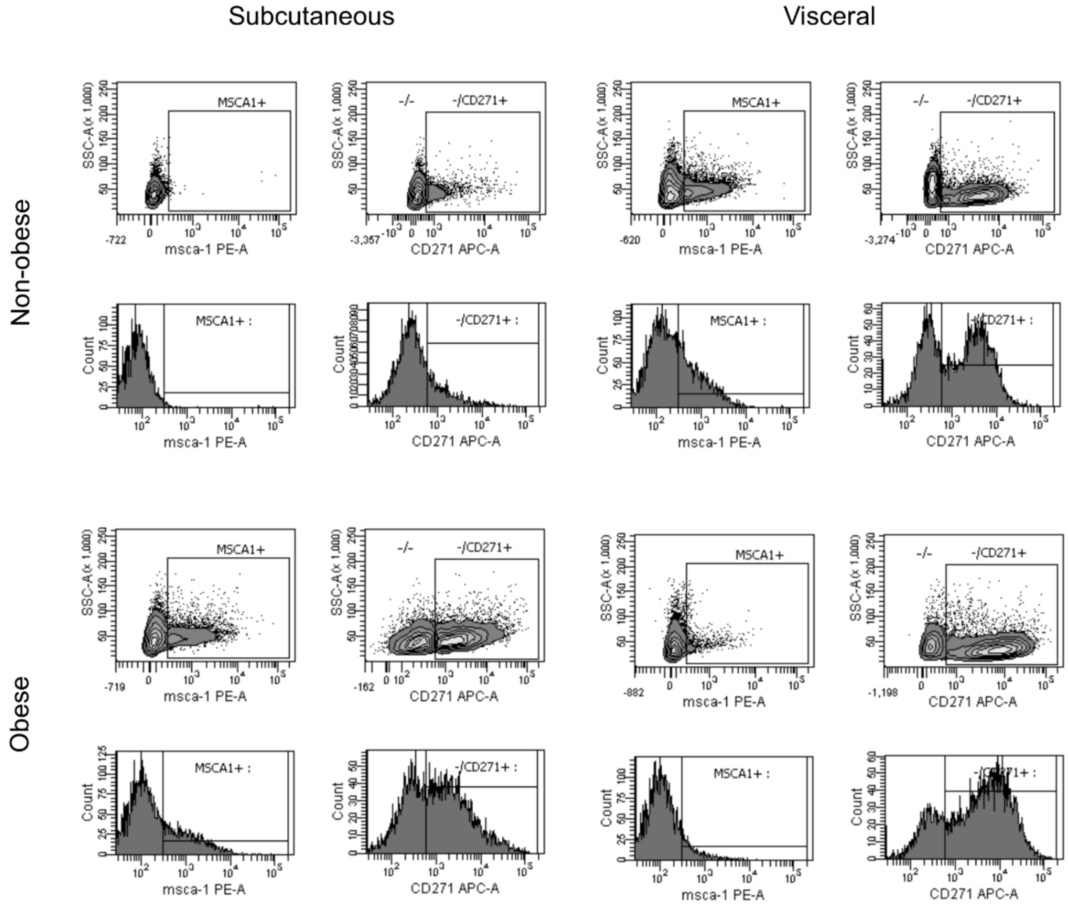

Supplementary Figure 4: flow cytometry analyses of non-obese and obese SC and VSAT progenitor cells

MSCA1+, -/CD271+ and -/- progenitor cell subsets were analyzed by flow cytometry on SVF cells isolated from paired subcutaneous (left) and visceral (right) AT, from lean (up) and obese donor (down) corresponding to the data presented in Figure 8. Representative dot-plots and histograms of MSCA1 and CD271 stainings gated in the CD45-/CD31-/CD34+ progenitor population (same gating strategy shown in Supplementary Figure 2). The experiment was performed on 23 non-obese and 123 obese women.

Supplementary Figure 5

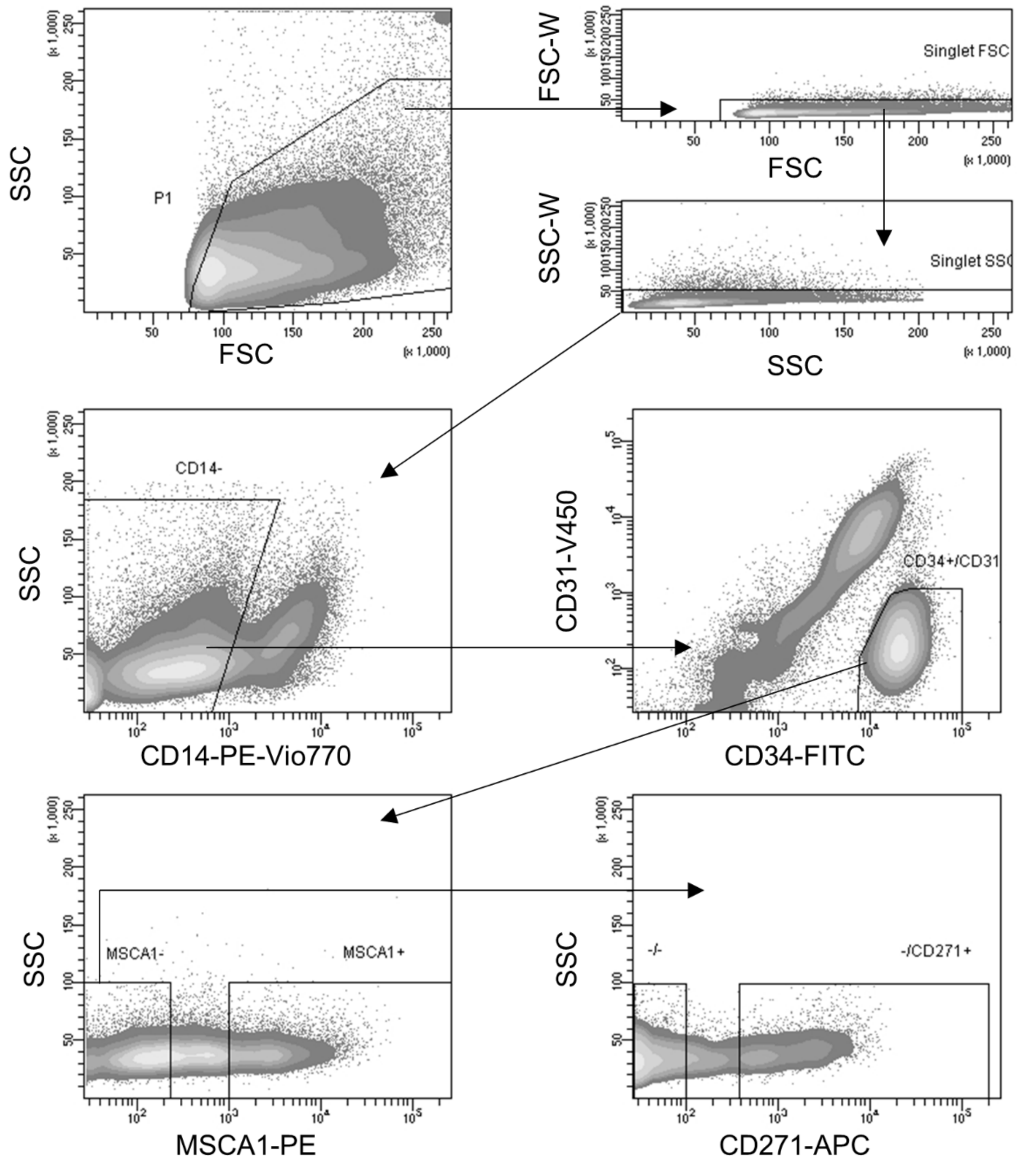

Supplementary Figure 5: Gating strategy for progenitor subset sorting

Gating strategy to sort the progenitor subsets from freshly isolated stromal vascular cells. Doublets were eliminated using FSC and FSC-W, then SSC and SSC-W. CD14 negative cells were gated into single cells. CD34+/CD31- progenitor cells were gated into CD14- cells. Then, MSCA1+ progenitor cells were gated into CD34+/CD31- progenitor cells and sorted. -/CD271+ and -/- progenitor cells were gated into MSCA1 negative cells and sorted.

Supplementary Table1

	Non-obese women	Obese women without metabolic syndrome	Obese women with metabolic syndrome
Number of participants	23	57	66
Age (years)	43,32 ± 8,67 (21,37 - 59,38)	37,54 ± 8,53 (24,8 - 58,07)	44,62 ± 10,55 (18,91 - 64,64) ##
BMI (kg/m²)	23,92 ± 2,66 (18,73 - 28)	43,67 ± 6,23 (26,1 - 63,4) ***	44,15 ± 6,32 (33,9 - 62,8) ***
Waist (cm)	82,23 ± 8,3 (70 - 98)	117,65 ± 13,92 (96 - 152) ***	122,63 ± 12,57 (96 - 152) ***
Hip (cm)	97,05 ± 9,21 (78 - 108)	132,35 ± 10,68 (101 - 160) ***	127,11 ± 12,71 (99 - 169) ***, #
Waist/Hip ratio	0,85 ± 0,07 (0,72 - 0,9694)	0,89 ± 0,09 (0,6621 - 1,118) **	0,97 ± 0,1 (0,7619 - 1,31) ***
Fasting Glucose (mmol/l)	4,93 ± 0,38 (4,24 - 5,66)	5,1 ± 0,42 (4,1 - 6)	6,36 ± 1,55 (4,4 - 10,6) ***, ###
HbA1c (%)	5,33 ± 0,35 (4,5 - 5,9)	5,54 ± 0,41 (4,6 - 6,8)	6,23 ± 0,95 (4,9 - 10,7) ***, ###
Triglycerides (mmol/l)	0,95 ± 0,51 (0,41 - 2,48)	0,92 ± 0,35 (0,42 - 2,06)	1,66 ± 0,8 (0,47 - 4,34) ***, ###
Total Cholesterol (mmol/l)	5,13 ± 1,15 (3,36 - 8,66)	5,1 ± 0,97 (3,33 - 7,61)	5,27 ± 0,83 (2,96 - 7,14)
HDL Cholesterol (mmol/l)	1,57 ± 0,45 (0,93 - 2,79)	1,34 ± 0,29 (0,8 - 2)	1,09 ± 0,22 (0,6 - 1,7) ***, ###
LDL Cholesterol (mmol/l)	3,13 ± 0,84 (2,12 - 5,89)	3,34 ± 0,87 (2 - 5,6)	3,43 ± 0,79 (1,2 - 5,3)
CRP (mg/l)	3,35 ± 3,75 (0,1 - 15)	10,74 ± 7,64 (2 - 44) ***	10,30 ± 7,5 (2 - 43) ***
Gamma GT (U/l)	16,52 ± 5,09 (8 - 25)	35,54 ± 22,12 (10 - 124) **	44,91 ± 23 (19 - 157) ***, #
SGOT / ASAT (U/l)	17,78 ± 4,8 (11 - 34)	22,23 ± 9,63 (10 - 68)	24,06 ± 10,53 (11 - 65) **
SGPT / ALAT (U/l)	16,61 ± 9,59 (7 - 55)	31,79 ± 17,44 (11 - 96) **	37,24 ± 18,73 (15 - 96) ***
SBP (mmHg)	118,65 ± 16,02 (94 - 149)	123,72 ± 9,97 (94 - 144)	136,52 ± 12,63 (101 - 160) ***, ###
DBP (mmHg)	75,78 ± 7,68 (64 - 90)	65,82 ± 9,16 (41 - 87) ***	73,06 ± 9,54 (35 - 91) ###
Number of participants with: high blood pressure medication	1	4	23
dyslipidemia medication	0	0	11
diabetes medication	0	0	17
impaired fasting glucose	0	0	30
low HDL	7	26	62
high TG	2	2	25

Supplementary Table1: Donor characteristics from SENADIP cohort.

Data are presented as mean +/- standard deviation (SD), SD range is indicated in between parenthesis for each group of patient. One way ANOVA followed by Tukey's multiple comparison test, ** P<0.01, *** P<0.001 compared to non-obese group, # P<0.05, ## P<0.01, ### P<0.001 obese without metabolic syndrome group compared to obese with metabolic syndrome group.

Supplementary Table 2: list of transcripts specific for -/-, -/CD271+ and MSCA1+ subsets isolated from subcutaneous AT

Gene name followed by the fold change over indicated subset and raw P value

Up in -/- cells from SC

Gene name	FC vs -/CD271+	raw P value	FC vs MSCA1+	raw P value
AADACL2	20,1	0,0012	5,5	0,0327
ABCA3	3,6	4,06E-05	2,3	0,0015
ABCB5	5,8	8,84E-05	2,0	0,0374
ADGRG2	28,5	0,0003	5,7	0,0227
ADGRG6	8,8	2,36E-05	3,6	0,0019
AHNAK2	2,6	0,0012	2,3	0,0038
AIF1L	6,2	0,0056	3,3	0,047
AMACR	3,1	0,001	2,0	0,0196
ANOS1	4,1	0,0168	3,5	0,0295
APCDD1L-AS1	4,8	0,0208	6,8	0,007
AQP1	2,2	0,0285	3,7	0,0015
ARL4C	4,4	0,0008	2,8	0,0089
BMP3	9,0	0,0128	5,3	0,0466
BMP7	12,9	8,18E-05	4,5	0,0051
BTC	2,7	0,0112	3,0	0,0061
C1QTNF3	14,1	0,0012	4,9	0,0263
C1QTNF3-AMACR	23,2	0,0019	16,7	0,0041
CADM3	2,4	0,0391	2,6	0,0272
CCR1	3,2	0,0042	2,2	0,0334
CD24	8,5	0,0064	6,0	0,0172
CD55	2,5	0,0043	2,5	0,0042
CDA	3,2	0,0014	2,6	0,0062
CDKN2A	3,4	0,0158	3,6	0,0129
CDKN2B	2,3	0,0239	2,1	0,0394
CDS1	4,5	0,0034	5,8	0,0011
CKMT2	2,1	0,0121	2,2	0,0079
CLEC3B	2,7	0,0013	2,0	0,0124
DKK1	3,2	0,0247	3,0	0,0333
DUOX2	3,8	0,0025	4,4	0,0012
EFHD1	2,4	0,0282	2,3	0,0426
ELL3	2,6	0,009	2,7	0,0068
ELMO1	2,6	0,0077	2,0	0,0365
EMILIN2	2,8	0,0018	2,2	0,0089
ERBB3	4,7	0,0097	3,1	0,0462
ERC2	2,0	0,0099	2,3	0,0029
F5	4,1	0,0247	3,6	0,0366
FAM133A	5,2	0,0059	3,1	0,0423
FAM19A5	6,8	0,0224	7,5	0,0172
FAM69C	4,0	0,0098	4,6	0,0055
FGF16	3,2	0,0028	3,2	0,0033
FHOD3	3,5	0,0084	2,6	0,0349
FNDC1	7,9	8,05E-06	3,5	0,0008
FNDC5	3,4	0,0017	2,1	0,0278

GALNT15	2,2	0,0324	2,1	0,0453
GATM	4,0	0,0038	2,9	0,018
GDF15	3,2	0,0063	2,7	0,0149
GGT1	2,5	0,0386	3,2	0,0126
GLRB	5,2	0,0015	2,8	0,0246
GPER1	2,4	0,0047	2,3	0,0071
GUCY1A2	6,5	0,0038	3,8	0,0268
HIST3H2BB	2,1	0,0017	2,3	0,0006
HSPA1L	2,8	0,0159	2,8	0,0165
HSPA7	2,7	0,0283	3,1	0,0167
IGFBP5	3,4	4,34E-05	2,3	0,001
IGFBP6	2,6	2,18E-05	2,0	0,0003
IL17RD	2,4	0,0003	2,0	0,0017
IL22RA1	2,6	0,0022	4,5	4,94E-05
ITGA11	4,1	8,20E-05	3,0	0,0008
ITGBL1	2,3	0,0329	3,1	0,0066
KCNJ16	12,8	0,0006	5,5	0,0087
KIAA1671	2,4	0,0264	2,4	0,0294
KIRREL3	4,5	0,0176	5,6	0,0087
KLHL4	9,6	0,0004	3,8	0,0134
LGI1	8,1	0,0066	6,8	0,0111
LINC01133	3,0	0,0208	2,7	0,03
LRRC17	2,6	0,0122	2,4	0,0203
LTBP1	6,0	0,0007	2,8	0,0236
LUZP2	6,9	0,0012	3,9	0,0116
MAPRE2	3,8	0,0077	2,8	0,0308
MAST4	2,1	0,0305	2,2	0,023
MATN4	15,0	0,0003	5,6	0,0087
MET	2,2	0,0321	2,1	0,0379
MMP27	3,2	0,0045	2,4	0,0212
MPP7	5,6	4,39E-05	3,9	0,0004
MUC13	2,5	0,0097	2,1	0,0317
MYO5B	5,0	0,0314	8,9	0,006
MYOM3	2,9	0,0031	2,9	0,0033
NCAM1	3,8	0,0234	6,3	0,0037
NCAM1-AS1	3,9	0,0035	3,7	0,0048
NEDD9	2,7	0,0076	3,0	0,0041
NTM	6,5	2,66E-05	3,1	0,002
NTN1	3,4	0,0084	5,6	0,0008
NTN4	2,9	0,0034	3,2	0,002
PAMR1	3,1	0,0008	2,8	0,0018
PCSK6	7,2	0,0017	2,9	0,0487
PDE10A	2,8	0,0147	2,7	0,0189
PDGFA	2,2	0,0087	2,5	0,0028
PDGFC	3,3	0,0009	2,2	0,0137
PIEZO2	6,5	0,0004	3,3	0,0097
PNLIPRP3	3,7	0,0138	3,5	0,0179
PRG4	9,5	0,0003	4,9	0,0035
PSD3	2,8	0,0004	2,3	0,0022
PXDNL	18,1	0,0003	5,6	0,0109
QPCT	2,1	0,0429	2,3	0,0306
SEMA3C	5,0	0,0004	2,1	0,0492

SFRP4	4,4	0,0167	5,1	0,0105
SHISA6	4,0	0,0056	4,1	0,0051
SPAAR	2,6	0,0493	6,4	0,0013
SPAG17	2,6	0,0012	2,0	0,0086
SPRR2E	4,8	0,0001	3,3	0,0012
TEK	4,5	0,0118	4,8	0,0095
TFCP2L1	3,2	0,0447	3,6	0,0294
TMEM255A	3,3	0,0049	2,7	0,0123
TMEM35A	6,1	3,61E-05	2,7	0,0045
WIF1	4,6	0,0012	3,5	0,0048
WNT10B	7,2	0,0043	4,5	0,0207
WNT4	11,5	0,0011	4,5	0,0218

up -/CD271+ from SC

Gene name	FC vs -/-	raw P value	FC vs MSCA1+	raw P value
A2M	4,3	0,004	2,4	0,0122
ACE2	2,9	0,0484	7,3	0,0012
ADAMTSL2	6,1	6,71E-07	2,1	0,0091
ADCY1	2,1	0,0016	2,1	0,0039
ADD2	2,9	0,017	7,3	0,0019
ADGRB1	4,2	0,0074	4,2	0,0177
ANKRD29	2,1	0,003	2,0	0,0014
ANO1	2,5	0,0172	2,8	0,0277
APLNR	16,7	0,0062	12,5	0,0274
ARHGAP15	7,6	0,0007	6,9	0,0056
ARHGAP30	2,7	0,0133	3,7	0,0008
ATRNL1	4,7	0,0007	4,6	0,006
BRSK1	2,5	0,0026	2,2	0,0304
CAMK4	2,1	0,0101	3,1	0,0028
CARD16	2,3	0,0158	2,3	0,0061
CBLN1	4,2	0,0147	7,8	0,0036
CCL13	4,1	0,0184	4,6	0,031
CCL21	8,9	0,0077	3,1	0,0342
CD200R1	2,6	0,031	2,7	0,0457
CGNL1	4,4	0,0015	6,1	0,0008
CHRM3	4,0	0,0169	3,7	0,0486
CHRNB4	6,5	0,0011	4,0	0,0214
CLDN1	16,0	0,0061	14,6	0,0228
COL21A1	2,9	0,0023	2,8	0,0086
COL4A4	7,3	0,0026	2,4	0,0424
CORIN	2,9	0,0046	3,5	8,45E-05
COX4I2	3,0	0,0061	4,7	0,0009
CRLF1	8,9	0,0003	5,5	0,0072
CX3CL1	3,7	0,0461	3,5	0,0125
CYP19A1	6,4	0,0004	3,0	0,0064
DCLK3	2,8	0,0083	2,4	0,037
DCX	12,7	9,56E-06	8,8	0,0005
DIO2	4,6	2,10E-06	5,8	1,07E-05
DYNC1I1	2,8	0,0004	3,5	0,0011
EDNRA	3,1	0,0028	2,7	0,0086
ELOVL2	7,0	1,41E-05	3,6	0,0028

EMID1	3,0	0,0047	3,5	0,0023
ENTPD2	3,6	0,0263	9,9	0,005
EPB41L2	2,0	0,0283	2,1	0,0374
EPHA3	14,6	5,75E-06	29,4	1,56E-05
EVA1A	3,3	0,0042	2,1	0,0305
F2RL2	2,1	0,0134	2,6	0,0048
FAIM2	3,3	0,001	2,3	0,0232
FGF12	2,6	0,0041	2,3	0,0246
FGF13	3,4	0,0196	3,9	0,0048
FMN2	6,9	0,0006	4,5	0,0144
FRZB	6,8	0,0003	3,2	0,0265
FZD10	3,0	0,001	3,5	0,0008
FZD10-AS1	3,8	0,0087	3,0	0,0027
GIPC3	10,6	0,0009	4,4	0,0082
GNAO1	2,6	0,0031	2,2	0,0013
GNAS	2,9	0,001	3,4	0,0028
GP1BA	4,2	0,0015	3,1	0,0151
GPR162	2,9	0,0053	2,8	0,0042
GRIK3	4,9	0,0079	5,8	0,0179
HES4	2,3	0,0001	2,2	0,0013
HEYL	6,8	0,0001	7,7	0,001
HHIP	12,5	5,82E-06	33,2	5,50E-06
HMX3	2,3	0,0396	3,4	0,0198
HR	3,1	0,003	2,9	0,0159
IGFBP7	7,2	1,02E-07	4,3	4,36E-05
IGFN1	17,6	8,99E-08	21,7	3,81E-06
IKZF3	2,6	0,0117	2,8	0,009
IL10RA	2,4	0,012	2,3	0,0332
JAK3	3,6	1,40E-05	2,3	0,0041
KCNIP1	3,6	0,0113	4,0	0,0124
KCNMB4	2,0	0,0015	2,0	0,0028
KLK1	7,6	6,25E-05	6,4	8,43E-05
LAMB2P1	2,5	0,0262	2,3	0,0414
LRRC55	5,0	0,0001	4,1	0,0009
LSAMP	3,2	0,0032	2,5	0,0357
LY75-CD302	3,1	2,05E-05	2,9	8,98E-05
MAP2	3,9	0,003	4,2	0,0018
MDFI	2,8	0,0142	2,1	0,0463
MEOX2	2,9	0,0085	3,9	0,005
MRAP2	7,4	0,0004	4,6	0,0118
MYL4	3,3	0,0264	2,9	0,0447
MYOC	2,5	0,0462	4,2	0,0099
NDUFA4L2	3,2	0,0335	3,4	0,0062
NGFR	13,5	1,45E-08	2,4	0,0028
NLGN3	2,8	1,85E-05	3,1	0,0003
NOTCH3	3,5	1,36E-06	3,0	4,06E-05
NOV	2,5	0,0084	6,5	0,0002
NOXRED1	2,3	0,0149	2,3	0,049
NTRK3	6,6	0,0066	11,1	0,0046
NUDT10	2,5	0,0036	3,0	0,0033
OXCT2P1	2,3	0,019	2,3	0,033
P2RY14	4,8	0,0019	3,8	0,0168

PAG1	3,5	0,0002	3,9	0,0009
PCDH17	2,6	0,0245	8,0	0,0007
PDE3A	2,1	0,0005	2,1	0,0033
PDLIM2	3,9	0,0018	3,0	0,0077
PDZD4	3,5	0,0035	2,3	0,0024
PGR	2,6	7,72E-05	3,2	1,38E-05
PKNOX2	3,0	0,0038	2,5	0,0287
PLD5	14,0	0,0002	14,6	0,0013
PLPPR4	3,2	9,18E-05	2,6	0,0042
POM121L9P	2,5	0,0063	2,2	0,0192
PPFIA2	2,7	0,0087	7,2	5,81E-06
PREX2	2,8	0,0007	2,5	0,0006
PTCH2	5,4	5,35E-07	6,7	1,40E-05
RASSF2	2,2	0,0137	2,5	0,0098
RBM11	3,2	0,0407	2,3	0,0274
RELN	3,7	0,0017	2,2	0,0415
RNASE10	2,1	0,001	2,1	0,008
RUNX2	4,3	4,79E-06	2,7	0,001
RXRG	2,8	0,021	2,6	0,0344
SAMD11	10,7	0,0002	3,3	0,0281
SCG5	2,6	0,0464	2,6	0,0473
SCN4B	6,3	5,29E-05	9,0	1,49E-06
SCN7A	11,8	1,96E-06	6,7	0,0003
SEMA5A	6,9	3,86E-06	3,0	0,0018
SEMA5B	6,0	0,0011	2,9	0,0406
SERPINA3	2,1	0,0085	3,0	0,0009
SERPINE2	2,9	0,005	2,1	0,043
SHANK1	9,3	1,27E-05	2,9	0,0043
SHOX2	2,4	0,0053	3,3	0,0034
SLC7A3	3,9	0,0088	2,8	0,0491
SLC8A3	4,9	0,0005	5,0	0,0026
SLCO2B1	8,0	5,23E-06	4,6	0,0006
SLIT2	2,4	5,04E-06	2,1	0,0002
SNAI1	3,3	0,0012	2,2	0,0286
SOCS2-AS1	2,6	0,005	2,9	0,0044
SOSTDC1	7,2	7,84E-06	7,2	0,0003
SOX8	2,0	0,0167	2,1	0,004
SPARCL1	2,8	0,0211	2,8	0,0122
SPINT1	2,4	0,0372	2,6	0,0237
SSTR2	4,2	1,54E-05	2,0	0,006
STMN2	8,9	0,0085	8,0	0,0172
SUSD2	4,9	5,24E-05	2,2	0,0291
SV2A	3,8	0,0113	4,7	0,0052
SV2B	4,6	0,0011	9,0	0,0009
SYNDIG1	11,6	0,0003	7,7	0,0038
TAC1	2,9	0,0055	2,8	0,0234
TENM2	6,5	0,0018	8,3	0,002
TENM4	4,1	8,23E-05	3,4	0,0023
THBS4	5,2	0,0207	4,2	0,0423
TLL1	2,9	0,0034	4,1	0,0011
TMC4	5,5	5,49E-05	4,3	0,0015
TMEFF2	11,2	8,31E-05	9,5	0,0003

TMEM233	2,1	0,0052	4,8	8,32E-05
TNFAIP8L3	4,1	0,0014	8,4	0,0001
TOX	12,3	0,0002	11,6	0,0006
TRNP1	2,5	0,0091	3,4	0,0067
TRPC6	6,5	0,0006	5,1	0,0073
TSPAN11	5,7	1,36E-05	2,7	0,006
TSPAN8	5,3	0,0001	6,3	0,0004
UNC5B	2,5	8,28E-05	2,6	5,06E-05
VAV1	2,5	0,0213	2,4	0,0148
VDR	2,1	0,0007	2,3	0,0007
VIPR2	4,4	1,79E-05	2,6	0,0016
VMO1	2,4	0,0007	2,9	0,0005
VSTM2L	5,3	0,002	7,0	0,0023
ZFP69B	2,4	0,0129	2,5	0,0122

up in MSCA1+ from SC

Gene name	FC vs -/CD271+	raw P value	FC vs -/-	raw P value
ADAM12	2,6	0,0489	3,3	0,0194
ALPL	18,8	7,60E-05	13,1	1,03E-05
BUB1	2,5	0,0396	3,4	0,0222
CHI3L1	3,2	0,0045	12,1	1,44E-05
CLEC2L	2,1	0,0055	2,9	0,0002
CPA1	3,0	0,0085	2,6	0,0134
EGFL6	3,2	0,0258	4,0	0,0165
GINS1	2,4	0,0293	2,6	0,0198
GRIA1	2,8	0,0029	2,6	0,0049
MEST	2,6	0,0067	2,3	0,0054
MOXD1	2,3	0,0123	2,9	0,0069
PRSS8	3,0	0,0208	2,3	0,041
RAD51AP1	3,3	0,0255	2,5	0,0494
SBSPON	3,9	0,0181	3,0	0,0277
SEPT4	2,7	0,038	4,1	0,0033
SLC25A18	2,5	0,004	2,6	0,0004
SLC7A10	3,3	0,017	3,8	0,0126
SLC7A8	2,3	0,0124	2,1	0,0193
SUSD3	3,0	0,0221	2,9	0,0214
TCF21	4,8	0,0178	3,8	0,0357
UBQLNL	2,2	0,0018	2,4	0,0115

Supplementary Table 3: list of transcripts expressed by the 3 progenitor subsets specifically in subcutaneous or visceral AT

Gene name followed by the fold change of indicated subset from a location over the same subset in the other location and raw P value

up in SC versus VS

Gene name	FC -/-	raw P value	FC -/CD271+	raw P value	FC MSCA1+	raw P value
AADAC	6,7	0,0036	30,2	2,01E-05	102,4	4,16E-08
ABCB4	25,1	7,82E-06	7,3	0,0002	7,7	0,002
ABCF1	2,4	0,0038	2,5	0,0166	2,3	0,0099
ADA	5,4	0,0003	2,4	0,0007	2,6	0,0052
ADGRE5	4,1	0,0002	3,6	0,0002	4,6	1,14E-05
AGMO	2,9	0,0375	3,3	0,0003	3,1	0,0159
AKR1B15	11,2	0,0068	10,9	0,0066	6,3	0,0141
ALKBH1	2,0	0,0031	2,8	0,0055	2,9	0,0011
ALX1	24,6	3,90E-07	12,8	0,0002	23,9	4,17E-07
ANGPTL4	4,4	0,0019	3,3	0,006	2,4	0,012
ANPEP	5,4	0,0005	4,0	0,0012	3,5	0,0006
APOC1	60,0	8,94E-06	19,7	5,90E-07	14,6	6,65E-05
AQP7	10,1	7,48E-05	3,2	0,0078	6,6	0,001
ARNTL2	2,8	0,0063	8,8	0,0018	6,7	0,0099
BCCIP	2,4	0,0085	2,2	0,045	2,1	0,0291
C11orf53	21,4	4,65E-06	13,1	2,17E-05	16,1	0,0014
C14orf180	84,2	3,30E-09	10,3	0,0021	18,3	0,0023
C14orf39	26,5	2,60E-07	19,6	0,0005	14,5	0,0004
C3orf36	7,2	2,15E-06	3,7	0,0009	3,1	0,008
C3orf67	9,9	3,13E-07	3,9	0,0219	4,4	0,007
C5orf38	21,0	3,35E-06	17,3	1,77E-06	16,5	0,0005
C6orf141	4,8	0,0231	3,3	0,0416	4,2	0,0143
C8orf76	45,9	0,0086	42,1	0,0282	366,4	4,71E-10
CA12	2,6	0,0101	2,3	0,0263	3,1	0,0043
CA2	5,8	2,11E-05	2,4	0,0073	4,5	0,0419
CACNB4	2,9	0,0025	2,9	0,0044	3,1	0,0006
CCL19	11,1	0,0068	14,3	0,0005	3,8	0,0104
CCNA1	4,9	0,006	9,7	0,0074	7,3	0,0001
CD36	39,8	5,15E-05	7,3	0,0032	10,9	0,0012
CD9	2,0	0,0473	3,5	0,0001	8,4	0,0002
CDH12	16,7	0,0052	11,1	3,86E-05	20,6	0,0004
CDHR4	3,8	0,0047	3,1	0,0237	9,3	0,0012
CDKL1	3,3	0,004	2,2	0,0072	2,7	0,0281
CDYL	2,3	0,0009	2,1	0,0009	2,4	5,52E-05
CEBPG	2,5	0,0065	2,5	0,013	3,0	0,0037

CFH	4,2	0,0003	5,3	4,19E-05	4,1	0,0006
CGREF1	7,3	0,0014	8,2	0,0005	3,4	0,0024
CHRNA5	2,5	0,0016	3,9	7,46E-05	2,5	0,0187
CIDEC	42,7	0,0001	7,0	0,0019	6,6	0,0067
CLIC6	11,6	0,0015	7,6	0,0002	8,5	7,48E-05
CMA1	4,2	0,0179	3,9	0,0463	4,8	0,0112
CNIH3	7,2	0,0001	4,2	0,0007	4,1	0,0039
CNTN3	31,2	1,66E-06	3,2	0,0081	5,4	0,0012
COPS8	2,2	0,0008	2,8	0,0029	2,5	0,0021
COX4I2	7,1	0,0006	16,0	0,0003	3,0	0,0404
CPM	11,8	0,0022	4,5	0,0006	5,5	0,0157
CPT1B	4,4	0,0123	2,1	0,0359	7,0	0,0196
CRNDE	97,2	6,24E-06	121,5	2,39E-08	93,3	1,57E-06
CRYM	3,1	0,019	4,1	0,0164	3,5	0,018
CSMD2	2,4	0,0042	5,7	0,003	3,9	0,0092
CTHRC1	24,9	0,0001	7,8	0,0006	5,3	0,0285
CTSG	3,5	0,028	10,7	0,0021	10,7	0,0031
CTSS	3,0	0,007	2,4	0,0003	2,1	0,0021
CXCL14	30,5	5,07E-09	2,9	0,003	2,5	0,0396
CYP19A1	5,2	0,0102	14,9	1,38E-06	5,3	0,0097
CYP4F12	2,0	0,0089	2,3	0,0037	3,1	0,0012
DCAF12L2	17,7	0,0003	24,7	1,47E-05	12,7	0,0007
DCLK3	4,6	0,0006	6,1	0,0005	4,3	0,009
DGAT2	16,4	0,0006	11,7	0,001	24,2	0,0003
DHRS9	22,6	0,0003	14,7	0,0002	25,0	0,0007
DIO2	4,7	0,0362	21,3	2,37E-07	5,1	7,06E-05
DKC1	2,7	0,0304	2,8	0,027	2,7	0,0209
DKK2	9,1	0,0005	65,3	2,30E-06	22,9	0,0003
DMRT2	57,2	3,49E-05	14,4	0,0024	11,4	0,0004
DTX4	4,2	0,0022	2,4	0,0063	2,5	0,0043
E2F3	2,9	0,0284	3,1	0,0409	3,0	0,0084
ECM1	5,4	0,0034	2,3	0,0191	4,0	0,0082
ELOVL4	2,9	0,0468	2,3	0,005	3,3	0,0011
EMCN	39,6	3,42E-06	31,2	2,11E-05	49,2	0,0011
EMX2	24,7	0,0003	19,6	0,002	47,5	4,37E-05
EMX2OS	29,7	7,12E-05	19,6	0,0007	35,0	0,0008
EN1	14,8	0,0005	23,4	4,28E-07	22,8	0,0016
ENTHD1	3,2	0,0012	3,8	0,0123	4,0	0,0251
EPHA4	3,4	0,0047	3,4	0,0074	5,2	0,002
ETS2	3,7	2,69E-05	3,1	0,0012	2,9	0,0043
F2R	4,4	0,0002	6,9	9,19E-05	5,3	0,0112
FAM196A	2,7	0,0149	3,0	0,0327	3,4	0,0007
FGFBP2	45,4	3,20E-06	8,0	0,0004	30,5	0,0003
FGL2	12,8	0,0005	5,6	0,0004	4,4	0,0042
FLVCR1	2,2	0,0145	2,5	0,0083	2,2	0,0472

FNDC1	5,1	0,0048	5,0	0,0009	9,7	0,0033
FNIP2	2,7	0,0048	2,7	0,0052	2,6	0,0019
FOXD2-AS1	6,6	0,0019	3,0	0,0096	3,2	0,0323
FOXF2	8,0	0,0002	4,4	0,0002	3,4	0,0019
FOXQ1	4,5	0,0082	3,4	0,0492	5,5	0,0081
FST	15,1	0,0003	3,0	0,0222	4,9	0,0023
FUT4	3,3	6,65E-05	2,4	0,0005	2,9	6,81E-05
GDF10	45,1	6,68E-08	10,5	1,15E-05	7,3	0,0002
GINS3	3,2	0,0006	2,9	0,0013	3,2	0,0007
GJC1	15,8	6,43E-05	6,2	0,0152	3,2	0,0428
GLDN	11,3	0,0001	10,2	0,0001	2,6	0,0179
GLRB	2,9	0,0413	3,0	0,0125	5,9	0,0055
GNA13	2,8	0,0003	2,2	0,0001	2,8	0,0001
GPBAR1	4,3	0,0194	3,4	0,0237	11,1	5,25E-06
GPD1	20,1	0,0005	13,5	0,0045	22,8	0,0008
GPR68	9,0	0,0003	11,3	0,002	6,5	0,0004
GRM7	9,2	0,0121	13,1	0,0014	17,0	0,0045
GRWD1	2,1	0,0131	2,5	0,0095	2,5	0,0122
HAGLR	3,9	0,0019	4,1	0,0017	3,5	0,0186
HAS2	5,3	0,0008	3,7	0,0001	4,5	0,0002
HAVCR1	10,0	0,0002	3,8	0,0004	8,0	0,0048
HDAC9	15,5	1,54E-06	17,9	1,77E-05	6,9	0,0208
HES1	7,1	0,0023	6,9	3,71E-05	5,0	0,0019
HIPK2	3,0	0,0092	3,3	0,0113	3,1	0,0112
HIVEP3	4,1	0,0472	2,7	0,0226	2,2	0,0487
HMGA2	18,9	0,0005	20,1	0,0014	12,7	0,0064
HMGCR	2,2	0,0056	2,5	0,0014	3,5	0,0005
HOXA10	6,6	0,0002	30,1	2,45E-05	31,2	4,11E-06
HOXA9	12,7	0,0001	18,5	5,20E-06	16,1	0,0011
HOXC10	58,2	6,39E-08	52,0	1,46E-05	77,2	2,90E-07
HOXC8	15,7	5,41E-06	30,6	2,88E-06	20,9	0,0011
HOXC9	62,1	3,01E-07	73,6	1,13E-07	30,9	0,0006
HOXD-AS2	23,7	1,16E-06	21,5	2,34E-07	13,6	0,0003
HOXD1	4,0	0,0025	5,2	0,0001	3,0	0,0088
HOXD3	3,1	0,0075	4,5	9,37E-07	3,3	0,0297
HOXD4	4,5	0,0005	5,9	0,0016	8,8	5,57E-05
HOXD8	29,5	3,79E-05	24,4	0,0001	18,4	0,0008
HOXD9	6,2	0,0015	3,6	0,0274	4,9	0,0211
HPGD	5,8	0,0136	4,7	0,0015	4,7	0,0083
HPSE2	15,0	0,0015	2,8	0,0246	10,4	0,0039
HS3ST2	43,7	0,0007	34,5	0,0003	24,5	0,0008
IGFN1	2,6	0,0031	22,6	0,0004	3,3	0,0001
IL7	3,9	0,0024	8,4	9,58E-05	8,7	0,0015
IMP4	2,0	0,0219	2,4	0,0403	2,2	0,0412
IMPAD1	2,3	0,0028	2,2	0,0165	2,2	0,0016

IRAK2	8,8	0,0008	3,5	0,0111	4,4	0,0111
IRF4	40,9	5,28E-06	9,7	0,0002	8,3	0,0002
IRX1	48,0	4,55E-06	245,8	5,78E-07	137,0	5,87E-05
IRX2	245,0	1,44E-10	137,6	8,61E-08	182,7	2,08E-07
IRX3	28,5	3,96E-05	16,0	1,66E-07	6,7	0,0007
IRX5	48,3	4,59E-08	71,8	4,66E-07	70,1	7,78E-07
ITGA4	14,0	3,57E-05	4,1	0,0001	2,6	0,0123
ITGA7	3,6	0,0004	7,8	5,02E-06	7,9	0,0006
ITGBL1	22,5	0,0002	25,4	1,77E-05	16,5	0,001
ITM2A	3,1	0,0275	2,6	0,0048	2,5	0,0333
KCNJ15	15,2	0,0002	21,5	0,0029	14,4	0,0044
KCTD5	2,3	0,0031	2,3	0,0445	2,2	0,0268
KDM7A	3,1	0,0008	2,7	0,0037	2,3	0,0138
KHDRBS3	2,3	0,0012	3,4	0,0001	3,3	0,0007
KIAA0930	3,7	1,06E-05	2,3	3,19E-05	2,5	1,44E-06
KIAA1549L	26,6	3,07E-06	35,1	4,35E-07	25,7	0,0012
KSR2	5,2	0,001	4,1	0,0493	7,2	0,0006
LANCL3	9,0	0,0016	9,2	0,0003	9,1	0,0041
LGR4	3,1	0,0055	5,3	1,55E-05	4,2	0,0004
LINC00645	10,6	9,79E-05	4,1	0,0039	7,4	0,0009
LINC01013	4,8	0,0063	6,2	0,0031	5,5	0,007
LINC01060	16,9	0,0002	23,0	0,0009	20,8	0,0188
LINC01116	12,1	0,0012	11,2	0,0049	5,0	0,0002
LOC154761	3,7	0,0011	2,1	0,0164	2,4	0,0472
LPL	22,9	4,23E-06	5,8	4,23E-05	3,0	0,0466
LRRC15	17,6	0,0002	7,7	0,0001	3,1	0,0084
LRRC32	5,0	0,0001	6,5	3,54E-05	3,8	0,0112
MAP1A	3,1	0,0008	3,1	0,0008	3,8	0,0002
MAP3K7CL	2,0	0,0058	4,2	4,08E-06	3,4	0,0088
MAPK13	2,5	0,0091	3,6	0,0048	2,1	0,0009
MARS2	2,9	0,0004	4,2	0,0097	3,6	0,0042
MAS1	6,4	0,0002	6,6	0,0027	2,9	0,0453
MC5R	2,2	0,002	2,3	0,0282	3,7	0,0092
MEOX1	3,2	0,048	4,1	0,0049	3,7	0,0386
MGC12916	5,8	0,005	7,0	0,0013	9,4	0,008
MINDY4	68,2	0,0005	36,9	0,0018	24,9	0,0046
MME	15,4	5,85E-05	5,8	0,0027	5,0	0,0149
MMP27	4,7	0,0083	8,1	0,006	13,5	0,0014
MMP3	7,8	0,0016	5,6	0,0023	3,8	0,0447
MRAP2	5,2	0,0006	20,8	0,0001	9,9	0,0022
MSR1	16,7	1,27E-07	5,3	0,0144	7,5	0,0053
MSX1	5,2	0,0101	3,1	0,0234	2,1	0,0486
MXD1	2,9	0,0002	2,1	0,0038	2,3	0,0028
MYADM	2,2	0,0017	2,2	1,53E-05	2,3	0,0002
MYH14	3,3	0,0198	8,4	0,008	9,8	0,0064

MYO1D	2,9	0,0466	4,5	3,86E-05	3,9	0,0005
NCOA5	2,3	0,002	2,3	0,0077	2,8	0,0002
NDNF	3,4	0,0322	8,9	1,46E-05	6,4	0,0005
NDUFAF4	2,4	0,0012	2,3	0,0153	2,4	0,0296
NFATC2	5,1	0,0017	3,7	0,002	2,6	0,036
NIFK	2,1	0,0226	3,3	0,0037	3,1	0,003
NIP7	2,5	0,0136	2,7	0,0165	3,1	0,0042
NKAIN1	5,5	0,0016	9,9	0,0001	4,1	0,0406
NLN	2,1	0,0169	2,9	0,0185	2,3	0,0428
NMUR2	16,6	0,0005	30,9	6,94E-07	47,0	3,96E-06
NNAT	7,9	0,0004	5,0	8,32E-05	3,4	0,0327
NOV	4,4	0,0028	29,4	1,79E-05	13,6	0,0005
NPAS2	2,2	0,0223	2,3	0,0052	3,1	0,0008
NRG2	3,5	0,0101	2,1	0,0217	4,5	0,0015
NRN1	47,2	2,96E-06	32,3	0,0002	60,5	3,56E-05
NUDT15	2,0	9,81E-05	2,5	0,0002	2,7	2,68E-05
OLFM1	3,5	0,0032	2,2	0,011	2,8	0,0271
PAX3	280,3	3,62E-06	597,3	1,54E-09	281,9	7,01E-07
PCSK1	25,0	5,66E-05	13,0	0,0007	8,8	0,0024
PDE1B	29,6	0,0001	2,2	0,0085	2,6	0,0024
PEAK1	2,8	0,0006	2,3	0,0017	2,1	0,0032
PHACTR2	2,4	0,0004	3,7	0,0001	2,2	0,0154
PLIN1	25,0	0,0011	7,8	0,0081	9,2	0,0087
PLXNB3	8,5	6,81E-05	2,5	0,0022	3,0	0,0034
PNLIPRP3	15,3	0,0002	4,7	0,0185	5,7	0,0023
PNP	2,8	0,0077	2,5	3,65E-06	2,0	0,0005
POLR1C	3,4	0,0004	2,7	0,0216	3,0	0,0153
POLR2D	2,2	0,0011	2,6	0,0054	2,3	0,0055
POPDC3	4,1	0,0068	16,7	0,0007	15,4	0,0002
PPAN	2,5	0,012	2,7	0,0394	2,9	0,0043
PPL	2,2	0,0055	3,0	0,0058	4,2	0,0013
PPM1L	5,3	0,0007	4,0	1,22E-05	5,9	6,90E-05
PRKG2	6,1	0,0022	17,0	3,95E-05	48,9	1,21E-05
PTCH2	11,8	1,56E-05	12,9	0,0003	5,0	0,0013
PTGER2	7,2	0,0014	2,5	0,0293	3,3	0,0162
PTGER3	3,9	0,0071	7,0	0,0003	4,5	0,0004
PTGES	2,8	0,0131	2,8	0,0004	2,8	0,0026
PTGFR	3,9	0,0015	3,1	0,0125	3,3	0,0009
PTPRE	4,9	0,0236	8,3	0,0066	8,4	0,0018
PTPRT	5,0	0,0006	4,6	0,0006	14,2	0,0048
PXDC1	2,8	8,94E-05	2,0	0,0016	2,1	7,58E-05
RANBP17	4,7	0,0179	5,3	0,0006	4,5	0,0099
RBM3	2,6	0,0009	2,4	0,0025	2,3	0,0067
RELN	3,6	0,002	3,7	0,0111	4,3	0,0009
REXO5	3,0	0,0039	2,1	0,0076	2,1	0,017

RPP40	2,0	0,0057	3,4	0,002	3,0	0,0062
RPSAP52	13,3	0,0043	17,9	0,0004	21,0	0,0072
RRN3	2,2	0,0038	2,1	0,0187	2,2	0,0036
RRS1	2,2	0,016	2,5	0,0291	2,7	0,0282
RSRP1	3,8	0,0005	2,8	0,0026	2,2	0,0151
S100P	7,2	0,0139	11,3	0,0086	9,1	0,0204
SACS	2,2	0,0048	2,1	0,0024	2,1	0,0039
SALL1	16,1	0,0006	12,3	0,0006	25,4	0,0081
SATB2	7,2	0,0011	6,4	1,45E-05	4,5	0,0036
SBK3	12,1	0,0007	3,7	0,0033	6,9	0,0041
SEMA3G	22,3	5,93E-05	10,5	0,0023	8,6	0,0018
SERINC2	2,3	0,0175	4,7	0,0017	8,4	0,0022
SERPINA5	78,4	5,24E-06	14,4	7,26E-05	19,6	0,0007
SERPINF2	5,0	0,0036	2,6	0,015	2,2	0,0395
SGIP1	6,1	0,0022	6,7	0,0007	10,7	0,002
SHC3	3,1	0,0121	2,6	0,0112	4,6	0,0493
SHISA2	15,8	1,76E-05	3,2	0,0006	4,5	0,0068
SIM1	11,3	4,45E-05	6,4	4,59E-06	10,4	7,78E-05
SIM2	7,3	0,0258	6,9	0,0271	12,4	0,0141
SIX1	5,9	0,0262	6,9	0,002	7,2	0,0007
SLC35G1	2,3	0,0079	3,0	0,0137	2,6	0,0057
SLC5A3	4,5	0,0002	3,4	0,0022	2,6	0,0487
SLC6A15	75,1	5,30E-06	57,8	5,76E-06	39,3	0,0007
SLC6A2	15,8	0,0012	9,1	0,0005	6,4	0,0029
SLCO2A1	26,7	8,02E-05	16,1	8,23E-05	22,7	3,83E-05
SMOC1	232,3	1,81E-06	16,4	0,0034	21,8	0,019
SNX7	2,1	0,0162	2,3	0,0001	3,4	1,15E-06
SORT1	10,1	4,50E-05	3,1	0,0095	9,0	0,001
SP9	11,3	0,0002	4,8	0,002	4,7	0,0089
SPAG17	32,5	7,55E-05	21,6	1,36E-05	25,4	5,60E-06
STAC	7,3	0,0002	5,4	0,0162	6,6	0,0111
STMN2	17,4	0,0004	155,7	4,56E-07	19,4	0,0012
SUCNR1	5,9	0,0149	7,4	0,012	5,8	0,0227
SYNM	6,7	0,0006	2,3	0,0222	2,8	0,0098
TAF5	3,2	3,90E-05	2,2	0,009	2,9	0,0005
TBX4	4,0	0,0031	3,1	0,0141	3,6	0,0228
TBX5	46,1	2,09E-05	53,2	2,34E-06	57,4	7,09E-07
TENM1	5,6	0,0068	39,1	1,60E-05	56,0	3,46E-06
TFIP11	2,2	0,0002	2,3	0,0009	2,4	0,0002
TM6SF2	3,0	0,0012	4,6	0,0153	2,8	0,0006
TMEM47	3,1	1,72E-05	2,6	0,0006	2,8	0,0002
TNMD	19,4	1,41E-05	5,1	0,0016	9,1	0,0015
TRABD2A	8,1	0,0059	5,1	0,0032	8,3	0,0018
TRIM35	3,1	4,84E-05	2,9	0,0108	4,1	0,0039
TRIM36	3,7	0,0056	2,3	0,0069	2,6	0,0315

TSPAN11	4,6	1,45E-05	3,5	0,0011	2,5	0,0131
TSPAN15	14,0	5,74E-05	3,4	0,0128	3,9	0,0161
TTPAL	3,3	0,0041	2,6	0,0248	2,9	0,0039
UPP1	3,7	0,0007	2,7	0,0222	3,1	0,0148
URB2	2,7	0,0158	3,3	0,0093	2,9	0,0066
USPL1	3,1	0,0002	2,4	0,0071	2,6	0,0096
UTP15	2,1	0,0281	2,3	0,0088	2,7	0,0034
VCAN	4,3	0,0213	8,1	0,0001	5,0	0,0261
VGLL4	2,2	0,0411	2,4	0,0127	2,2	0,0138
WDCP	3,0	0,0239	3,7	0,0004	4,1	0,0052
WDR47	2,1	0,0007	2,1	0,0016	2,2	0,0098
XG	44,7	1,08E-06	18,5	5,19E-07	37,1	8,39E-06
ZCCHC10	2,1	0,0009	2,2	0,0075	2,9	0,0028
ZNF296	3,5	0,0005	3,1	3,90E-05	2,4	0,0089
ZPR1	2,2	0,0131	2,6	0,0057	2,1	0,0398

up in VS versus SC

**Gene name**	**FC vs -/-**	**raw P value**	**FC -/CD271+**	**raw P value**	**FC vs MSCA1+**	**raw P value**
ACSS1	4,1	0,003	4,3	0,0008	7,3	0,001
ACTR3C	2,8	0,0394	6,6	0,0002	11,6	0,0011
ADAMTSL3	4,6	0,0025	4,3	7,91E-06	6,6	9,18E-05
ADCY6	2,3	0,0002	2,4	0,0002	2,5	0,0002
ADH1B	2,0	0,0253	4,2	0,0053	3,0	0,0228
ADORA1	5,0	0,006	5,7	0,0123	9,0	6,32E-05
AGTR2	2,5	0,0056	21,6	0,0002	18,5	0,0021
ALDH1A2	72,4	0,0002	87,8	1,14E-05	19,1	0,0114
ALDH1L1	9,2	0,0096	7,1	5,28E-05	7,1	4,28E-06
ALDH5A1	2,5	0,0327	2,0	0,0251	3,5	0,0076
ALOX15	1579,1	9,00E-06	425,3	5,54E-08	38,5	0,0116
ANGPTL7	178,9	8,36E-06	12,2	9,91E-05	11,8	0,0098
ANKRD29	6,4	0,0007	2,1	0,038	5,3	0,0008
ANKRD46	3,5	0,0382	3,5	0,0107	4,5	0,0127
ANXA8	1923,0	7,83E-06	434,1	1,39E-07	43,6	0,0111
ANXA8L1	971,2	1,78E-05	195,5	9,77E-06	33,2	0,0089
ANXA9	97,8	2,02E-05	23,0	2,25E-05	2,8	0,0296
AP1M2	18,0	4,92E-05	2,2	0,0495	2,2	0,0175
ARHGEF28	43,3	6,49E-05	10,1	0,0065	27,6	0,0002
ARID5B	3,7	0,0163	4,4	0,0038	4,2	0,0023
ARNT2	10,0	0,0012	4,4	0,0003	4,5	0,0037
ARRDC1-AS1	3,8	0,0035	2,3	0,0018	4,1	0,0154
ART4	4,7	0,0251	24,4	0,0009	71,0	6,76E-05
ASTN1	63,6	0,0002	21,9	0,0003	13,0	0,0008
ASTN2	4,0	0,0008	3,9	0,0117	13,7	0,002

ATP1B1	7,8	0,0004	4,5	0,0002	5,7	3,03E-05
BARX1	194,9	2,61E-07	329,4	1,60E-07	251,2	0,0002
BCAR3	5,5	0,0169	3,1	0,0104	2,7	0,0455
BCHE	7,2	0,0043	4,7	0,002	3,7	0,0152
BICDL1	69,8	2,20E-05	8,5	0,0003	4,4	0,0459
BST2	2,7	0,0098	2,7	0,0055	3,0	0,0211
BTBD8	2,5	0,0299	7,0	0,0004	7,3	0,0093
C17orf107	3,1	0,0292	3,3	0,0148	3,3	0,0101
C4A	88,3	8,11E-05	7,4	0,0019	5,7	0,0003
CACNA1H	14,6	0,0009	2,4	0,0324	2,7	0,0002
CAND2	3,2	0,0045	2,1	0,0074	2,3	0,0482
CATSPERB	12,2	0,0027	9,8	0,0003	9,0	0,0031
CAVIN2	22,8	0,0118	8,5	0,032	8,0	0,0075
CBLN4	33,2	0,0002	296,6	3,41E-07	227,9	1,31E-05
CBX1	3,0	0,0006	2,2	0,0398	2,2	0,02
CCDC102B	2,6	0,0032	4,2	0,0045	9,7	0,0004
CCDC68	7,6	0,0226	4,3	0,0225	5,6	0,0034
CCL11	11,8	0,0061	60,2	5,73E-06	283,8	6,38E-07
CD4	4,7	0,0057	4,2	0,0149	10,0	8,67E-05
CDCA7L	4,7	0,0084	2,7	0,003	2,8	0,0046
CDH4	9,3	9,99E-05	7,6	0,0004	4,2	0,0214
CDIP1	2,8	0,001	2,5	0,0019	2,8	0,0017
CDK18	14,3	0,0005	6,5	0,0052	7,8	0,0017
CDK20	2,6	0,0088	3,1	0,0363	2,3	0,0325
CELSR2	4,6	0,0116	3,7	0,0157	3,4	0,0452
CERS6	3,7	0,0061	3,2	0,0017	3,4	0,0041
CETN3	2,6	0,0105	2,4	0,0247	3,0	0,0055
CFI	40,8	2,55E-06	16,8	4,66E-06	19,9	1,51E-05
CHN2	4,8	0,0015	4,9	0,0014	6,3	0,0185
CITED4	3,8	0,0008	3,2	0,002	2,6	0,0301
CLDN1	654,1	1,91E-06	5,3	0,0036	27,6	0,0105
CLDN11	2,5	0,0104	6,5	1,02E-05	3,1	2,04E-05
CLDN15	48,7	2,27E-05	6,1	2,12E-06	2,9	0,0282
CNN1	11,9	0,0023	10,6	0,0005	15,8	0,0059
COL4A6	3,1	0,0479	3,0	0,0071	2,7	0,0489
COL9A3	26,1	0,0014	4,1	0,0086	9,6	0,0007
COLGALT2	3,4	0,0022	5,2	2,43E-05	6,0	0,0003
CPNE5	15,6	0,0003	3,9	0,0066	6,7	7,95E-05
CPT1C	8,6	5,50E-07	3,6	0,0013	5,1	0,0002
CSDC2	53,2	2,80E-05	27,5	0,0003	13,6	0,0056
CSGALNACT1	4,2	0,0213	3,1	0,0049	6,3	0,0055
CSRP1	2,3	0,0016	2,9	0,0002	3,0	4,60E-05
CTNNA2	30,6	6,02E-05	8,6	0,0005	4,6	0,0422
CTTNBP2	2,1	0,0064	2,5	0,0003	2,7	0,0286
CXCL13	14,8	7,93E-05	8,4	0,0056	5,5	0,0071

DAPK1	13,7	0,0005	3,1	0,0035	3,3	0,0055
DBNDD1	14,4	0,0002	17,1	0,0006	12,4	0,0004
DDR1	6,6	0,0002	3,1	0,0062	3,4	0,0021
DDX60	4,5	0,0004	3,0	0,007	2,8	0,0012
DES	135,5	3,46E-05	30,7	0,0006	35,3	0,021
DNAJB1	3,2	0,0007	6,9	6,01E-06	4,1	0,0054
DNAJC12	5,0	0,0126	2,6	0,014	4,1	0,0076
DOCK4	3,9	0,0031	5,0	0,0005	2,5	0,0147
DOCK5	9,3	0,0014	8,1	4,28E-05	5,2	0,0003
DPYS	22,0	0,0003	18,6	7,36E-05	18,4	0,0005
DSC3	41,5	0,0001	7,8	0,0008	3,4	0,0158
DTX3	3,1	0,0004	2,7	0,0007	2,6	0,0014
DUS4L	2,2	0,021	2,2	0,0259	2,2	0,0185
EDN1	2,9	0,0384	2,6	0,0454	5,3	0,0042
EGFL6	176,1	0,0006	26,4	0,0026	5,9	0,011
EIF4A2	2,1	0,0011	2,2	0,0007	2,5	3,76E-05
ELL3	2,1	0,02	3,7	0,0147	4,1	0,0097
EMB	164,2	1,02E-07	9,1	0,0002	4,0	0,0431
EPHA2	4,7	0,0036	3,1	0,0023	2,6	0,014
EPYC	7,8	0,0109	24,1	0,0005	20,7	0,0015
ERBB4	39,5	4,63E-05	5,2	0,0083	3,8	0,0447
ESR1	3,0	0,0032	5,4	0,0002	6,9	0,0008
EYA2	5,6	0,0183	25,7	0,0002	8,8	0,0001
FADS2	5,4	0,0017	4,1	0,0018	4,9	0,0038
FAIM2	3,1	0,001	2,1	0,0359	2,5	0,0286
FAM131B	3,7	0,0005	4,7	7,03E-05	8,0	0,0121
FAM153C	102,2	3,63E-05	16,2	0,0027	4,9	0,0433
FAN1	2,1	0,0194	2,7	0,0093	2,6	0,0356
FAT3	17,3	3,06E-06	4,4	0,0024	7,9	0,0003
FBP1	10,7	0,0001	4,1	0,0348	5,5	0,001
FLNC	4,3	0,0001	2,2	0,0053	2,4	0,0437
FLRT3	24,4	0,0002	18,7	3,97E-06	4,1	0,0224
FMN2	25,2	0,0003	4,9	0,0175	22,2	0,0006
FNTB	4,3	0,0005	2,9	0,0098	3,5	0,0083
GALNT14	39,8	0,0006	9,5	0,0014	6,3	0,0063
GATA5	24,3	0,0003	4,2	0,0261	4,2	0,0075
GATA6	3,1	0,0026	2,4	0,01	4,0	0,0046
GNA14	3,6	0,0084	4,9	0,0016	11,7	0,0001
GNG4	2,7	0,0018	4,4	0,0011	8,1	0,0004
GPRASP2	3,0	0,0049	2,6	0,005	3,7	0,0003
GREM1	6,8	0,0158	27,3	0,0002	150,0	2,23E-05
HAND2-AS1	3,9	0,002	4,5	0,0023	3,4	0,002
HBA1	3,8	0,0012	5,9	0,0002	4,4	0,001
HBA2	5,5	0,0051	10,5	0,0002	6,4	0,0009
HBB	9,3	0,0055	13,9	1,21E-05	9,9	0,0038

HBEGF	3,1	0,0049	2,7	0,004	2,1	0,0072
HEPACAM	32,4	6,72E-05	5,0	0,0001	3,3	0,002
HERC4	2,1	0,0002	2,3	0,0006	2,0	0,0057
HINT3	3,2	6,04E-05	3,1	4,56E-05	2,3	0,0054
HLTF	2,6	0,0011	2,3	0,0219	2,0	0,0498
HMGCL	2,8	0,0075	3,4	0,008	2,4	0,0137
HOOK1	29,2	7,25E-06	5,5	0,0024	2,4	0,0082
HOTS	3,4	0,0025	4,3	0,0015	4,8	0,0008
HOXA3	4,2	0,0005	2,8	0,0366	4,1	0,0067
HOXA4	15,0	0,0003	6,2	0,0095	7,5	0,0069
HOXB9	2,6	0,0077	5,5	0,0059	7,4	0,0032
HS3ST1	45,0	0,0001	8,7	0,0011	4,3	0,0497
HSF4	2,6	0,0182	5,0	5,86E-06	2,9	0,0055
HSPA1A	3,0	0,004	4,9	7,22E-05	5,1	4,03E-05
HSPA1B	4,2	0,0034	4,7	0,0006	4,5	0,0008
HSPA6	8,5	0,0059	20,7	0,0015	15,0	0,0013
HSPA7	17,2	0,0006	18,6	0,0006	18,5	0,0006
IGFBP2	36,6	0,0003	29,0	1,45E-05	59,8	3,29E-05
IKZF4	2,9	0,0087	3,4	0,0041	3,5	7,63E-05
IL20	22,6	3,37E-05	5,3	0,0078	4,8	0,0227
INMT	13,0	0,0002	6,4	0,0009	12,1	0,0011
INMT-MINDY4	8,9	0,0003	3,9	0,0023	29,9	0,0023
IPP	2,2	0,0018	2,6	0,0067	2,5	0,0191
ISL1	69,4	3,63E-06	71,6	1,03E-07	30,1	0,0005
ITLN1	2911,3	7,15E-06	900,8	1,20E-06	124,2	0,0085
JAKMIP2	5,0	0,0021	31,1	0,0005	11,1	0,0044
JPH2	25,2	0,0049	7,0	0,0015	3,4	0,0404
JPH4	14,9	0,0018	6,8	0,0138	6,1	0,0007
KANK4	27,6	0,0003	85,0	3,82E-05	103,3	4,62E-05
KCNB1	14,8	0,0006	9,5	0,0006	4,0	0,0384
KCND1	2,3	0,0167	3,3	0,0039	3,2	0,0013
KCNN3	18,0	9,24E-06	39,4	4,43E-05	17,6	0,001
KCTD16	4,0	0,0034	7,9	0,0033	14,1	0,0001
KDM2B	3,2	0,0142	3,1	0,0451	3,2	0,0072
KIAA0319L	2,6	0,0012	2,5	0,0153	2,2	0,0409
KIAA1211	18,7	0,0003	8,0	0,0016	11,4	0,0012
KIAA1755	2,7	0,0141	4,2	0,0034	4,3	0,0091
KLK11	652,1	2,71E-06	98,5	2,95E-07	17,7	0,0156
KLK5	275,4	3,71E-05	31,5	0,0001	7,4	0,0393
KLK8	15,8	0,0001	2,6	0,0269	2,0	0,0413
KNDC1	22,4	0,0002	3,4	0,0346	5,0	0,0021
KRT18	1417,5	1,87E-05	179,7	4,96E-05	69,2	0,0047
KRT19	1543,6	1,36E-05	296,9	3,19E-06	49,8	0,0115
KRT222	6,0	0,0257	4,9	0,0089	8,0	0,0289
KRT5	614,4	5,44E-07	77,1	7,80E-05	15,4	0,0159

KRT8	725,9	7,90E-06	116,4	1,47E-05	28,9	0,0042
LAMB1	2,2	0,0075	3,3	4,80E-05	4,6	3,28E-05
LAMP5	5,9	0,0186	27,9	0,0012	33,7	0,0089
LDLRAP1	2,1	0,0103	2,5	0,0043	3,2	0,0009
LGALS2	88,8	3,75E-05	10,2	0,003	5,5	0,0009
LIMS3-LOC440895	9,5	0,0127	4,9	8,67E-05	3,8	0,0098
LINC00665	8,2	0,0012	2,3	0,0109	2,2	0,0217
LINC01018	70,5	1,57E-05	11,2	5,18E-06	5,9	0,0033
LINC01096	8,4	0,019	7,5	0,0088	23,9	0,0002
LINC01638	4,2	0,0045	2,9	0,0045	6,8	0,0001
LOC642852	4,4	0,0039	3,3	0,0007	8,5	0,0068
LOC728392	6,1	0,0002	2,4	2,75E-05	3,7	0,001
LRCH4	2,6	0,0075	2,1	0,0141	2,4	0,0108
LRFN5	2,5	0,0475	3,9	0,0036	3,7	0,0036
LRRN4	514,6	4,82E-06	96,0	1,40E-06	10,4	0,0348
LRSAM1	2,2	0,0411	3,0	0,0128	2,5	0,0016
LTBP1	8,9	0,0059	9,8	0,0006	3,3	0,0328
MALL	2,5	0,0218	3,7	0,0028	4,8	0,0012
MAP6	8,8	0,0007	2,1	0,0224	3,3	0,008
MAPK12	9,1	0,0189	5,4	0,0004	11,6	0,0003
MAST3	3,6	0,0008	2,8	0,0118	4,5	0,0018
MAST4	3,5	0,0023	2,6	0,0045	2,2	0,0316
MDFI	4,7	0,0068	18,0	5,56E-05	60,0	3,15E-05
MEIS1	3,3	0,0066	4,0	0,0006	3,5	0,0016
MMP23A	2,6	0,0192	2,2	0,0144	3,4	0,0089
MORN1	2,2	0,0167	2,2	0,0092	2,5	0,0027
MOV10	2,1	0,0016	2,1	0,0001	2,0	0,0089
MSLN	1701,4	1,71E-06	339,9	2,00E-07	51,7	0,0092
MST1R	6,7	0,0046	2,6	0,0293	2,7	0,0135
MT1B	10,4	0,014	6,2	0,0198	5,1	0,0445
MT1E	5,9	0,0022	5,8	0,006	4,9	0,0063
MT1G	27,8	0,006	17,7	0,0038	12,2	0,0128
MT1H	15,5	0,0177	11,3	0,0148	8,6	0,0188
MT1JP	3,0	0,0291	6,1	0,001	4,0	0,0095
MT1L	4,2	0,0125	3,0	0,0086	4,5	0,0009
MYRF	168,1	1,50E-05	18,9	8,38E-05	14,2	0,0015
NAPEPLD	4,1	0,001	2,7	0,028	2,3	0,0169
NAV2	6,1	0,0002	11,5	5,59E-05	29,5	0,0002
NBAS	2,3	0,0005	2,1	0,0007	2,1	1,90E-06
NBEA	5,8	0,001	3,6	0,0003	3,5	0,0032
NDC80	5,9	0,0024	7,3	0,001	10,7	0,0014
NECAB1	11,8	0,0004	45,8	0,0004	20,7	0,0002
NECTIN2	4,4	0,0005	2,6	0,0009	2,3	0,0022
NELL1	43,8	4,86E-05	9,0	0,0017	10,3	0,0092

NFASC	6,6	0,0146	69,3	5,31E-07	116,6	2,20E-08
NKX3-2	16,7	0,0008	26,0	5,80E-05	32,5	3,36E-05
NOL3	2,2	0,0251	3,3	0,0045	6,7	0,0002
NPNT	79,0	0,0001	92,6	7,00E-05	146,1	0,0003
NPTXR	6,4	0,0001	5,1	1,85E-05	6,2	0,0135
NR2F1	14,8	0,0002	32,2	4,58E-06	21,9	9,00E-06
NR2F1-AS1	6,4	0,0009	5,5	0,0033	2,7	0,0259
NRARP	5,3	0,0056	4,1	0,0042	5,7	0,0005
NRK	8,6	0,0003	5,1	0,0017	27,2	0,0007
NSG1	302,5	3,35E-06	19,8	6,20E-05	8,2	0,0274
NTNG1	3,6	0,0053	4,3	0,0007	4,4	0,0095
NYNRIN	3,7	0,0045	3,5	0,0148	4,0	0,0033
OLR1	30,3	0,0006	8,8	0,003	4,6	0,032
P2RX2	15,6	0,0005	2,6	0,0004	2,3	0,0099
P3H2	5,2	2,99E-05	22,1	2,27E-06	29,9	7,18E-05
PARD6B	51,8	8,36E-05	17,6	0,0001	6,0	0,0154
PBX1	2,1	0,0022	2,5	0,0019	2,2	0,0091
PCDHAC2	45,7	0,0002	6,9	0,0028	5,7	0,006
PCK1	12,5	0,0016	12,3	0,0001	8,4	0,0059
PDE1A	3,8	0,0239	4,7	0,0006	2,2	0,0104
PDE9A	2,4	0,0306	2,4	0,002	2,7	0,0115
PDLIM2	28,1	0,0005	4,7	0,0058	23,9	0,0001
PDZK1IP1	160,8	2,85E-05	14,7	1,10E-05	7,6	0,0159
PGM5P3-AS1	3,0	0,0469	6,7	0,0014	9,1	0,0046
PIK3CD	6,3	0,0009	3,9	0,0049	3,2	0,0027
PLA2G4A	2,4	0,0039	2,5	0,0001	2,5	0,0017
PLAT	7,4	0,0006	10,1	0,0001	20,8	4,43E-05
PLCB4	6,7	0,0437	36,8	0,0012	40,2	2,24E-05
PLEKHA2	3,8	0,0033	4,5	0,0007	3,6	0,0025
PLEKHA6	2,4	0,0137	2,4	0,0329	3,8	0,0203
PLEKHG3	6,6	0,0008	4,1	0,0016	3,6	0,012
POLR3G	3,6	0,0121	3,1	0,0002	2,4	0,0061
PON3	2,4	0,0113	3,7	0,0002	3,8	0,0034
PPFIA3	4,2	0,0093	2,9	0,022	5,2	0,0062
PPP1R9A	4,5	0,0095	10,0	5,51E-05	8,2	0,0001
PREX1	4,3	0,0208	3,2	0,0194	4,8	0,0002
PRKG1	2,2	0,0095	2,2	0,0006	2,1	2,19E-05
PTN	8,7	5,11E-05	55,5	5,92E-07	48,9	1,61E-06
PTPRQ	19,2	0,0003	7,1	0,0028	7,1	0,0016
QPRT	20,2	0,0003	5,9	0,0004	17,1	2,30E-06
RAB11FIP4	5,8	0,0024	2,3	0,0003	2,7	0,0133
RAMP1	41,8	5,94E-05	12,3	0,0017	26,3	0,0001
RAPGEF3	4,9	0,0148	5,1	0,0026	7,4	3,69E-05
RARB	9,8	0,0003	6,5	0,0013	5,5	0,0217
RARRES1	23,5	9,74E-06	11,6	3,24E-05	3,5	0,0347

RBP1	5,5	0,0056	7,2	0,0004	6,4	0,0001
RDH10	2,1	0,0417	2,7	0,0056	2,7	0,001
REC8	18,1	0,001	4,4	0,0009	4,4	0,0242
RGL1	2,7	0,0047	2,5	0,0067	2,0	0,0395
RIMBP2	3,7	0,0084	4,2	0,0018	4,6	0,0144
RIMS1	15,1	0,0015	9,1	5,45E-06	20,6	0,0014
RIPK4	166,8	7,18E-06	17,4	5,08E-05	5,5	0,0414
RNF150	3,5	0,0314	8,5	0,0002	10,3	1,95E-05
RNF207	3,2	0,0012	3,6	0,0165	3,1	0,001
RNF214	4,3	9,98E-05	2,9	0,0153	2,5	0,043
ROR2	14,2	0,0001	8,1	0,0001	10,9	0,0001
RSPO1	215,0	8,11E-06	19,0	0,0001	17,3	0,0021
RTP4	6,1	0,0077	3,2	0,0254	2,8	0,0171
SCG5	17,9	0,0002	2,5	0,0112	7,2	0,0023
SCIN	4,1	0,0046	17,0	9,64E-05	22,8	0,0001
SCN2B	3,1	0,027	4,3	0,0004	3,9	0,0124
SCN7A	2,7	0,04	2,6	0,0167	5,7	0,0263
SEMA3B	12,0	4,08E-05	35,1	3,45E-05	8,3	0,0022
SEPSECS-AS1	3,2	0,0267	4,3	0,014	7,2	0,0004
SEZ6L2	72,8	0,0004	8,6	0,0011	10,4	0,0137
SFMBT2	2,4	0,0013	2,6	0,0017	2,1	0,0045
SFRP2	2,1	0,0264	10,1	4,18E-05	8,2	0,0004
SGPP2	83,4	2,19E-05	10,8	0,0013	4,9	0,0114
SH3GL3	4,9	0,0017	2,3	0,0248	6,7	0,0003
SKINT1L	7,6	0,0037	4,0	0,0011	3,7	0,0099
SLC16A14	2,6	0,0438	6,1	0,0004	4,1	0,0003
SLC16A4	4,2	0,0051	2,1	0,0064	3,0	0,0065
SLC16A9	5,0	0,0229	3,3	0,0427	8,9	0,0024
SLC24A3	3,4	0,0012	7,1	1,01E-08	5,2	3,34E-06
SLC30A1	4,2	0,041	7,2	0,0142	6,0	0,0176
SLC45A3	3,7	0,0279	3,5	0,0281	7,2	0,0098
SLC4A11	6,7	0,0015	45,4	3,41E-06	50,7	2,87E-07
SMIM1	2,7	0,0188	3,5	0,0101	4,9	0,0004
SMTNL2	19,8	0,0023	12,9	0,0001	5,8	0,0341
SORL1	5,4	0,002	2,9	0,0457	7,8	0,0005
SOX6	46,9	2,81E-05	9,8	0,0029	4,9	0,0363
SPARCL1	6,3	0,0036	6,6	3,74E-06	12,4	4,40E-05
SPEG	23,0	0,0002	6,2	0,001	4,4	0,0477
SPON1	7,2	0,0072	3,3	0,0013	7,5	0,0002
SRGAP2	2,0	0,0187	2,5	0,0109	5,0	0,0277
ST6GAL2	5,4	0,0092	3,7	0,0078	4,2	0,0135
ST6GALNAC5	14,7	0,0077	15,3	0,0008	10,6	0,0463
STAMBPL1	2,5	0,0005	2,9	3,64E-05	2,1	0,0276
STK36	2,5	0,0337	2,4	0,0234	2,3	0,0369
SULT1C4	8,9	0,0015	49,8	1,54E-05	84,0	1,29E-06

SUSD3	11,7	0,0039	6,7	0,0152	3,9	0,0065
SYNPO2	10,1	0,0008	5,9	0,0008	10,4	0,0041
SYT4	231,2	2,91E-08	14,9	0,0002	5,2	0,0222
SYTL4	2,2	0,0005	2,4	0,0053	3,2	0,0247
TACR3	6,7	0,009	3,6	0,0229	4,7	0,0253
TC2N	3,3	0,0285	3,7	0,0114	3,5	0,0106
TCF21	91,4	1,29E-05	207,3	9,00E-07	50,4	7,78E-05
TFPI2	3,9	0,0134	2,0	0,0147	2,9	0,0011
TGM1	381,1	2,36E-05	43,9	1,36E-05	10,4	0,0364
TGM2	50,6	0,0005	9,6	0,0042	5,6	0,0293
THAP2	3,2	0,0028	2,2	0,0238	2,0	0,0006
THSD4	18,0	5,07E-05	3,2	0,0073	4,3	0,0381
TMEM132B	11,5	0,0006	4,2	0,0065	3,7	0,0031
TMEM161B	2,6	0,0025	2,4	0,0069	2,0	0,0152
TMEM246	12,0	0,0028	5,9	0,0215	9,8	0,0197
TMOD2	2,5	0,0097	5,5	0,0001	4,4	0,0006
TNFSF14	5,0	0,0058	3,1	0,0082	5,1	0,0451
TNS3	4,2	0,0056	3,1	0,0006	4,0	0,0009
TPD52L1	11,1	0,0004	3,5	0,0054	5,5	0,0008
TRIM17	22,3	5,74E-05	7,2	0,0018	4,5	0,0244
TRIM5	2,9	0,0004	2,4	0,0022	2,4	0,0004
TRNP1	40,2	5,16E-05	3,6	0,0008	13,8	0,0002
TST	11,6	0,0005	2,2	0,0242	2,2	0,0321
TTLL7	2,4	0,0029	3,1	0,0003	2,3	0,0146
TUBA4A	5,2	0,0002	4,8	1,21E-05	5,3	6,33E-05
TVP23A	14,7	2,61E-05	11,6	7,33E-05	12,8	0,0003
TXNIP	2,6	0,0049	3,1	0,0006	3,5	0,0077
UBR2	2,4	0,0079	2,2	0,0105	2,1	0,0134
UPK1B	350,3	2,26E-07	45,1	1,67E-05	7,2	0,0468
UPK3B	686,0	4,57E-05	91,1	9,93E-05	12,0	0,019
VIPR2	7,7	0,0006	3,1	0,0013	5,8	5,24E-05
VSNL1	63,1	0,0001	14,3	0,006	10,3	0,0365
WDR6	4,2	0,0021	2,7	0,004	2,8	0,0034
WDR86	3,2	0,0204	15,4	0,0002	8,2	0,0001
WDR86-AS1	20,1	0,0009	20,9	0,0002	5,7	0,013
WNK2	2,6	0,0069	13,3	1,15E-05	9,0	0,0016
WNK4	48,6	3,20E-05	19,5	5,20E-07	4,4	0,0249
WNT5A	13,1	1,75E-05	7,3	0,0006	9,4	0,0012
WT1	279,5	2,42E-08	206,9	1,75E-05	131,6	3,39E-05
ZBTB46	2,5	0,027	2,2	0,0189	2,6	0,0134
ZFHX2	2,6	0,0111	3,9	0,0121	4,4	0,0039
ZNF185	15,7	7,20E-05	3,9	0,0001	2,3	0,0496
ZNF204P	3,2	0,0029	2,6	0,0114	5,9	0,0097
ZNF229	8,9	0,0002	4,0	0,0079	2,4	0,0451
ZNF302	2,5	0,0427	3,4	0,0133	3,6	0,0149

ZNF467	6,6	0,0018	4,0	0,0161	6,9	0,0023
ZNF471	2,7	0,0115	3,1	0,0098	3,5	0,0024
ZNF483	10,9	0,0002	5,4	0,0078	3,7	0,0381
ZNF608	4,8	0,003	7,0	0,0001	7,3	0,0001
ZNF70	2,5	0,01	2,0	0,004	2,6	0,0467
ZNF75D	2,1	0,027	2,1	0,0298	2,7	0,0079
ZNF775	3,1	0,0311	3,4	0,0233	4,1	0,0278
ZSCAN31	5,0	0,0246	4,6	0,0307	4,0	0,0424

Supplementary Table 4: list of transcripts specific for -/-, -/CD271+ and MSCA1+ subsets isolated from visceral AT

Gene name followed by the fold change over indicated subset and raw P value

up in -/- from VS

Gene name	FC vs -/CD271+	raw P value	FC vs MSCA1+	raw P value
AADAC	5,8	0,0016	14,0	5,70E-05
AADACL2	11,9	0,0187	29,7	0,0029
ABCA3	4,9	1,70E-05	9,3	4,96E-07
ABCC6P1	3,8	0,027	5,9	0,0055
ABCG1	6,8	0,0073	7,5	0,0054
ABHD4	2,3	0,0109	2,0	0,0275
ABLIM1	5,0	0,0001	10,0	3,18E-06
ABRACL	2,9	8,57E-05	3,3	3,40E-05
ACE	2,6	0,0476	2,7	0,0374
ACKR2	4,2	0,0062	3,3	0,0171
ACOT11	3,1	0,0259	4,9	0,004
ACOT7	2,2	0,0032	2,4	0,0015
ACP6	2,1	0,0113	2,5	0,0024
ACSBG2	5,2	0,0045	7,7	0,001
ADAL	2,6	0,0285	2,4	0,0426
ADAM10	2,4	3,92E-05	3,4	1,72E-06
ADAM15	2,4	0,0007	2,7	0,0002
ADAMTS16	3,7	0,0261	3,4	0,0352
ADAMTS6	7,7	0,0008	9,7	0,0004
ADCY8	12,3	0,0059	20,2	0,0018
ADGRD1	3,4	0,0005	5,7	2,30E-05
ADGRG2	18,2	0,0073	42,9	0,0013
ADGRG6	9,9	0,001	43,5	1,16E-05
ADI1	2,7	4,30E-06	2,4	1,49E-05
ADIRF	2,6	0,0191	4,1	0,0021
AFAP1L2	3,7	1,46E-05	7,1	2,32E-07
AGTPBP1	3,0	7,22E-06	3,4	2,80E-06
AHNAK2	2,3	0,0015	4,2	1,34E-05
AIF1L	8,0	0,009	9,8	0,0052
AJUBA	4,8	0,0313	4,5	0,0366
AKAP6	3,2	0,0408	3,8	0,0217
ALCAM	2,6	0,0214	3,6	0,0039
ALDH3B1	5,7	1,09E-05	6,6	4,75E-06
ALDOA	2,1	0,0456	2,6	0,0154
ALOX15	9,9	0,0008	30,7	2,54E-05
AMACR	2,8	0,0245	3,6	0,008
AMHR2	5,0	0,0335	10,7	0,0042
AMIGO3	2,4	0,0014	2,5	0,0012
AMMECR1	2,9	0,0144	3,0	0,0118
ANGPTL7	12,6	8,66E-05	16,3	3,49E-05
ANKEF1	4,8	0,0059	5,2	0,0041
ANKRD30B	6,9	0,0096	8,0	0,0061
ANO5	7,4	0,0036	17,1	0,0002
ANOS1	5,9	3,42E-06	6,8	1,63E-06

ANXA2	2,1	0,0143	2,0	0,0191
ANXA3	10,6	0,0018	39,6	4,30E-05
ANXA8	10,1	0,0025	27,6	0,0001
ANXA8L1	10,3	0,0023	23,0	0,0002
ANXA9	5,3	0,0018	15,7	2,62E-05
AOX1	2,7	0,0001	3,2	2,85E-05
AP1G2	2,2	0,0121	2,7	0,0029
AP1M2	7,2	0,0008	8,3	0,0005
AP1S2	2,3	0,0034	2,1	0,0078
AP1S3	4,8	0,0222	9,0	0,0033
APLP1	4,8	0,0053	4,7	0,0057
APOL2	3,2	0,0443	4,6	0,0119
AQP1	5,3	0,0013	8,9	0,0001
AQP9	9,0	0,0009	39,6	9,27E-06
AREG	3,8	0,0204	4,4	0,0108
ARFGEF3	7,1	0,0028	20,9	8,41E-05
ARHGAP28	4,3	0,01	2,8	0,0495
ARHGAP29	3,6	0,0002	4,7	2,84E-05
ARHGDIG	7,5	0,0036	4,4	0,0218
ARHGEF3	5,4	0,0007	6,6	0,0003
ARHGEF37	3,7	0,0428	9,9	0,0019
ARL4C	4,5	0,0004	5,2	0,0002
ARMC4	8,1	0,0003	9,6	0,0002
ARRB2	2,8	0,0028	4,2	0,0003
ASIC2	7,2	1,70E-05	6,5	2,84E-05
ASPHD1	8,4	0,01	15,0	0,0021
ATP13A2	2,4	0,021	2,2	0,032
ATP7B	3,6	0,0053	6,7	0,0003
ATP8A1	2,4	0,0061	3,8	0,0003
ATRNL1	7,4	0,0004	37,8	1,49E-06
AZGP1	2,7	0,0215	2,3	0,0423
B3GALT1	2,6	0,0499	3,2	0,021
B3GNT5	2,3	0,0096	2,1	0,0171
B9D2	2,1	0,0072	2,1	0,0077
BAIAP2L1	6,9	0,0013	14,1	9,56E-05
BAMBI	2,7	0,0002	2,6	0,0004
BANK1	3,4	0,0033	3,3	0,0038
BARX2	7,5	0,041	22,6	0,0041
BCAM	4,2	0,0091	7,7	0,0008
BCAS1	5,7	0,0369	15,7	0,003
BCAT1	2,4	0,0337	2,6	0,0233
BCO2	3,7	0,0009	5,5	8,44E-05
BEX1	3,7	0,0388	5,8	0,0085
BEX2	4,9	0,001	5,8	0,0005
BICDL1	10,4	0,0004	17,7	7,00E-05
BIRC3	3,3	0,0188	5,4	0,0025
BLOC1S1-RDH5	3,5	0,0026	3,5	0,0027
BMP3	7,9	0,0061	14,3	0,0011
BMP7	16,4	3,25E-05	26,8	6,74E-06
BMP8B	29,9	0,0038	41,9	0,002
BNC1	8,7	0,002	23,9	8,59E-05
BTC	7,4	0,0003	14,0	2,74E-05

C10orf107	2,9	0,012	5,0	0,0007
C10orf67	4,0	0,0061	7,3	0,0004
C11orf80	3,7	1,93E-05	5,2	2,13E-06
C12orf75	4,5	0,0026	2,5	0,0397
C15orf39	2,6	0,0042	3,4	0,0007
C17orf58	3,5	0,0249	4,4	0,0112
C19orf33	11,8	0,0033	27,2	0,0004
C1GALT1	2,3	8,27E-05	3,5	1,48E-06
C1orf226	5,6	0,0005	14,3	1,01E-05
C1orf53	4,7	0,0134	6,2	0,0052
C1QL1	5,5	0,0069	9,2	0,0011
C1QTNF3-AMACR	3,9	0,0257	4,8	0,0123
C2CD4C	2,8	0,0069	2,2	0,0238
C4A	4,0	0,0387	4,4	0,0287
C5orf30	2,6	0,0152	2,7	0,0104
C6orf132	5,3	1,08E-05	8,6	8,09E-07
CA10	4,1	0,0472	4,1	0,0472
CA11	3,2	0,0059	5,0	0,0005
CA9	11,8	9,30E-05	11,7	9,84E-05
CAAP1	2,1	2,96E-05	2,2	2,87E-05
CACNA1H	3,7	0,0198	5,5	0,0047
CACNB2	6,2	0,0007	7,9	0,0003
CACNG4	5,0	0,0035	12,1	0,0001
CADM3	6,3	1,26E-05	14,5	2,52E-07
CADM4	2,6	0,0312	3,0	0,0168
CALB2	11,3	0,0021	42,4	5,85E-05
CARD10	4,9	5,96E-05	6,1	1,81E-05
CARD16	4,1	0,0005	5,4	0,0001
CARNS1	7,8	0,0063	27,5	0,0002
CASC15	4,3	0,042	6,9	0,0106
CASZ1	3,2	0,0149	4,4	0,0038
CAV2	2,4	0,0024	2,2	0,0044
CAVIN2	4,8	0,0356	7,9	0,0089
CBLN2	5,2	0,0191	10,9	0,0021
CCDC183	2,7	0,0291	4,1	0,0046
CCDC51	3,5	0,0227	4,4	0,0096
CCDC6	2,1	0,0024	2,4	0,0009
CCDC69	2,6	0,0014	2,8	0,001
CCDC85A	2,3	0,037	2,4	0,025
CCDC85C	4,6	0,0045	7,5	0,0006
CCNYL1	3,1	0,0192	4,0	0,0063
CCSER1	3,1	0,006	5,5	0,0003
CD200	5,4	0,0002	6,2	0,0001
CD24	15,5	0,0027	7,7	0,0157
CD55	5,0	0,0172	6,8	0,0062
CDA	5,6	0,0141	5,5	0,0149
CDCP1	7,0	0,0094	17,5	0,0007
CDH1	4,5	0,0141	10,4	0,0008
CDH3	7,7	0,0199	28,1	0,0009
CDHR4	2,1	0,0194	4,0	0,0003
CDKN2A	5,2	0,0319	6,0	0,0225
CDS1	4,6	0,0006	5,1	0,0004

CELSR1	4,6	0,0103	9,6	0,0007
CERS1	3,1	0,0166	4,0	0,0054
CFAP57	3,2	0,0476	3,9	0,0226
CFB	4,5	0,0002	6,9	1,63E-05
CFL2	2,0	0,0088	2,1	0,007
CFTR	4,8	8,91E-05	6,0	2,67E-05
CGN	10,6	0,0012	18,8	0,0002
CHGB	4,6	0,0464	4,8	0,0405
CHMP4C	4,1	0,0003	5,7	3,92E-05
CHST15	5,2	0,0005	2,6	0,0175
CHST4	13,6	0,0014	18,6	0,0006
CLDN1	7,7	0,0019	21,6	6,63E-05
CLDN15	7,0	0,0005	11,8	6,91E-05
CLDN16	7,0	0,0018	4,6	0,0088
CLIC3	10,6	1,22E-05	19,3	1,20E-06
CMAHP	3,2	0,0097	3,9	0,0037
CNTN4	3,2	0,05	8,8	0,0016
CNTN6	4,7	0,0044	7,4	0,0007
CNTNAP3	6,2	0,0061	17,4	0,0002
COBL	9,3	0,0343	16,3	0,0112
COBLL1	3,7	0,0018	5,2	0,0003
COL2A1	2,8	0,047	3,3	0,0277
COL8A1	5,3	0,0496	10,9	0,0089
CORO1C	2,2	0,0006	2,4	0,0003
CPA4	6,0	0,0005	8,7	9,46E-05
CPNE5	5,5	0,0009	4,1	0,0033
CR1	5,8	0,0012	10,4	0,0001
CRB2	6,1	0,0153	16,6	0,0009
CRB3	3,5	0,0028	6,4	0,0001
CREB5	3,9	0,032	5,5	0,0108
CRIP1	2,9	3,66E-05	3,0	3,13E-05
CRIP2	2,4	0,0046	2,6	0,0028
CRMP1	4,1	0,0127	10,3	0,0004
CSNK2A3	3,4	0,0264	8,5	0,0008
CST6	8,0	0,0011	6,4	0,0026
CTNNA2	3,9	0,0024	4,4	0,0013
CTNNA3	6,8	0,0002	13,1	1,38E-05
CTSG	3,3	0,0407	4,3	0,0173
CTSV	3,3	0,0241	2,9	0,0387
CXADR	9,2	3,73E-05	27,5	6,53E-07
CXCL6	4,2	0,0312	4,3	0,029
CYFIP2	4,7	0,0213	9,5	0,0023
CYP21A2	2,2	0,049	2,4	0,031
CYP2J2	7,5	0,0008	20,3	2,43E-05
CYP2S1	10,7	0,0014	24,3	0,0001
CYP3A5	6,6	0,0053	25,5	8,15E-05
CYP4B1	5,4	0,044	5,9	0,0359
DACT2	15,0	0,0004	37,0	2,96E-05
DAPK1	3,9	0,0119	4,9	0,0044
DBN1	3,1	0,0093	3,0	0,0097
DBNDD2	4,2	2,29E-07	6,9	8,78E-09
DDI1	3,6	0,023	3,0	0,0432

DDR1	2,5	0,0183	2,4	0,0231
DEFB1	8,7	0,0424	9,6	0,0348
DENND3	3,3	0,0016	6,1	4,53E-05
DFNA5	4,2	3,32E-05	5,8	4,56E-06
DHCR24	3,2	0,0041	4,2	0,0009
DHCR7	3,3	0,002	5,2	0,0001
DHRS13	4,8	0,0016	2,8	0,0225
DIAPH1	3,5	0,0004	4,4	9,19E-05
DIAPH3	6,9	0,019	7,7	0,0143
DISP1	7,3	0,0002	14,6	1,00E-05
DKK1	5,2	0,0194	7,4	0,0068
DKK2	8,3	0,0011	4,3	0,0123
DLEC1	4,7	0,0067	3,9	0,014
DMTN	3,7	0,0416	7,0	0,0051
DNAAF3	7,1	2,18E-05	10,8	3,01E-06
DNAH5	2,5	0,0231	3,7	0,0029
DNAJC22	7,7	0,0127	27,6	0,0005
DNMT3L	4,9	0,0051	3,7	0,0158
DOC2A	3,1	0,034	3,0	0,0355
DOCK4	2,4	0,0282	2,8	0,0129
DPP10	5,0	0,0027	7,5	0,0005
DPP4	9,5	0,0002	17,7	1,59E-05
DPYSL2	3,7	0,0017	5,2	0,0003
DSC2	5,1	0,0013	8,8	0,0001
DSC3	6,2	0,0017	9,0	0,0004
DSG2	8,9	0,0005	18,8	3,95E-05
DSP	7,1	0,0167	41,9	0,0002
E2F5	2,1	0,0266	2,2	0,0199
E2F7	2,2	0,0393	2,5	0,0201
EBP	2,5	1,08E-07	2,7	4,43E-08
ECEL1	2,3	0,0294	3,9	0,0016
ECT2	2,5	0,0007	2,5	0,0007
EDEM2	2,8	5,99E-05	2,5	0,0001
EEF1A2	7,6	0,0104	14,8	0,0017
EFNA5	5,8	0,0002	11,2	9,28E-06
EMB	9,6	1,55E-07	18,5	8,94E-09
EMILIN2	2,2	0,0005	2,5	0,0001
EML5	3,8	0,0069	3,9	0,0066
EMP3	2,3	0,011	3,0	0,0016
ENC1	3,6	0,0223	6,1	0,0028
ENDOD1	2,1	0,0198	2,4	0,0074
ENPP4	4,6	8,25E-05	6,2	1,61E-05
EPB41	4,3	0,0036	5,8	0,001
EPB41L4A	2,6	9,79E-05	3,9	2,88E-06
EPB42	4,3	0,0165	8,8	0,0014
EPHB6	3,7	5,17E-06	5,8	2,43E-07
EPS8L1	7,2	0,0001	17,3	3,56E-06
ERBB3	11,9	3,76E-05	24,1	3,13E-06
ERBB4	8,1	0,0022	13,8	0,0004
ERC2	6,6	0,0031	12,4	0,0004
ERICH5	10,1	0,0002	18,1	3,00E-05
EVPL	6,8	0,0031	27,9	3,40E-05

EXOC6	2,6	0,0087	3,5	0,0012
EZR	7,9	0,0005	14,2	5,68E-05
F11R	5,4	0,0052	7,1	0,0019
F5	7,0	0,0253	7,4	0,0218
FAAH2	4,4	0,0075	5,3	0,0034
FABP3	3,1	0,0457	3,3	0,0386
FAM110C	5,1	0,025	7,0	0,0099
FAM133A	3,6	0,025	3,4	0,0324
FAM153C	7,0	0,0066	12,6	0,0011
FAM155A	7,6	0,0363	19,3	0,0048
FAM160A1	5,3	0,0153	11,3	0,0014
FAM163A	6,1	0,0372	16,6	0,0034
FAM167A	6,4	0,0022	5,7	0,0035
FAM189A1	6,1	0,0005	26,9	1,90E-06
FAM189A2	4,6	0,0248	37,7	5,05E-05
FAM19A3	4,6	0,0228	5,0	0,017
FAM212B	3,8	0,0167	6,9	0,0016
FAM213B	2,9	0,0047	2,0	0,0389
FAM221A	2,5	0,0093	2,5	0,0092
FAM3B	8,0	0,0006	11,0	0,0002
FAM46B	5,5	0,0051	3,8	0,0194
FAM49B	3,6	0,0001	4,6	2,64E-05
FAM69A	2,6	0,0072	2,3	0,0149
FAM78B	2,6	0,0382	3,4	0,0101
FAM83H	5,8	0,0103	10,2	0,0017
FAM84B	3,1	0,0383	5,3	0,005
FAM86JP	2,1	0,0215	2,2	0,0174
FANCF	2,6	0,0013	3,1	0,0003
FAR2	5,1	0,009	9,3	0,0011
FASTKD1	2,4	0,0072	2,5	0,0059
FAT3	3,3	0,0038	2,5	0,0182
FATE1	5,6	0,0012	5,6	0,0012
FBN1	2,9	0,0417	2,9	0,0429
FBP1	7,7	3,52E-05	3,9	0,0011
FBXO2	4,3	0,0068	6,9	0,001
FCRLB	7,1	0,0005	10,1	0,0001
FDXR	2,3	0,0306	4,0	0,0017
FGF1	8,9	0,0131	14,1	0,0042
FGF9	8,4	0,0012	19,6	7,70E-05
FGFBP1	6,1	0,0272	33,2	0,0004
FHDC1	2,9	0,0134	3,6	0,0043
FHOD3	5,8	0,0121	6,0	0,0114
FLJ27354	3,0	0,023	3,3	0,0152
FLNC	3,2	0,0071	2,3	0,0394
FLRT2	2,9	0,0443	3,8	0,0163
FLRT3	3,0	0,0218	3,7	0,0091
FNDC1	7,7	0,0032	6,6	0,0054
FOXI2	20,4	0,0001	15,7	0,0003
FRAS1	4,8	0,0442	13,8	0,0027
FRK	3,0	0,0384	3,2	0,0297
FRY	3,6	0,0303	4,6	0,0121
FUCA1	2,8	0,0008	2,5	0,0016

FZD6	4,7	0,049	11,0	0,0054
GAL3ST2	5,0	0,0104	11,0	0,0007
GALNT12	4,3	0,008	4,6	0,0059
GALNT14	4,9	0,021	8,6	0,0039
GALNT15	9,3	0,0006	14,0	0,0001
GALNT18	8,0	0,0004	8,7	0,0003
GALNT9	9,8	0,0133	45,3	0,0004
GALR1	7,9	0,0161	6,3	0,0281
GAP43	7,0	0,0013	8,3	0,0007
GATA5	5,8	0,025	5,7	0,026
GATM	6,1	0,0005	11,2	3,64E-05
GCNT1	5,5	0,0064	7,0	0,0027
GCNT2	4,8	0,0065	6,7	0,0018
GDF1	3,1	0,0158	4,1	0,0046
GFRA3	3,8	0,0092	4,1	0,0065
GGT1	3,2	0,0312	6,0	0,0029
GIPC2	2,5	0,0021	2,3	0,0047
GJA3	9,3	0,0006	20,8	3,76E-05
GKAP1	2,4	0,0385	2,3	0,0421
GLRB	5,4	0,0026	5,7	0,0021
GNAZ	6,7	0,0076	15,8	0,0006
GOLGA2P10	4,5	0,0023	5,5	0,001
GOLGA6L9	2,3	0,0033	4,3	3,56E-05
GPM6A	6,4	0,0049	18,6	0,0002
GPR1	9,8	0,001	15,4	0,0002
GPR143	9,6	0,0035	8,9	0,0044
GPR37	5,3	0,0001	9,6	7,27E-06
GPT2	3,9	0,0014	3,6	0,0021
GRB7	9,6	0,0045	11,5	0,0027
GRIK5	4,4	0,0073	22,3	1,99E-05
GRIN2A	5,0	0,0092	5,3	0,0071
GRIP1	5,2	0,0248	7,2	0,0095
GSAP	4,7	0,0078	6,7	0,002
GUCY1A2	5,8	0,0173	8,2	0,0063
HACD1	3,4	0,0016	3,0	0,0039
HAS1	5,6	0,0011	12,2	4,42E-05
HCFC1R1	3,2	0,0007	2,0	0,0177
HEG1	2,7	1,56E-06	3,4	1,85E-07
HEPACAM	7,9	6,42E-05	13,7	6,80E-06
HERC3	2,1	0,0021	2,4	0,0009
HERC5	2,0	0,0114	2,1	0,0086
HID1	5,2	0,0035	9,2	0,0004
HIST1H3D	7,6	0,0079	12,4	0,0019
HLA-F	2,9	0,0461	3,9	0,0144
HMSD	4,2	0,0097	3,0	0,0344
HNRNPA1P33	12,4	0,0022	29,2	0,0002
HOMER2	3,1	0,0056	3,3	0,0045
HOOK1	5,3	0,0037	12,0	0,0002
HOXA10	4,2	0,005	5,0	0,0023
HP	10,4	0,002	21,5	0,0002
HPCAL1	2,5	0,0015	2,5	0,0013
HPR	4,9	0,0224	7,8	0,0051

HPS6	2,0	0,0002	2,1	0,0001
HPSE	4,8	2,74E-06	7,2	2,27E-07
HRASLS5	3,6	0,0038	7,4	0,0001
HS3ST1	6,1	0,0077	12,2	0,0008
HSBP1L1	3,1	0,013	3,4	0,0093
HSD17B2	7,3	0,0066	14,5	0,0008
HSD17B6	11,8	0,0002	33,7	5,66E-06
HSD17B7	4,0	0,0005	4,4	0,0003
HSD17B8	2,2	0,0008	2,1	0,0014
HSD3B7	6,0	3,00E-05	6,8	1,59E-05
HSPB7	4,1	0,0031	3,5	0,0065
HUNK	5,2	0,0019	8,1	0,0003
HYAL1	5,0	1,41E-05	3,0	0,0005
IER3	2,5	0,0322	2,6	0,0233
IFI27	2,5	5,83E-06	3,1	8,38E-07
IFNE	3,7	0,0106	3,7	0,0102
IFNLR1	2,1	0,0262	2,5	0,0073
IGFL2	7,5	0,0162	24,3	0,0008
IGSF9	13,3	0,0001	38,6	5,08E-06
IGSF9B	2,9	0,0179	4,7	0,0017
IL17RE	6,5	0,0166	5,3	0,0292
IL18	7,8	3,19E-05	18,5	9,00E-07
IL1RAP	3,8	0,0088	6,6	0,0009
IL20	5,8	0,0023	11,0	0,0002
ILDR2	7,4	0,0034	11,2	0,0009
INO80C	2,7	0,0004	2,9	0,0002
INPP5J	2,7	0,0316	3,5	0,0087
IQCA1	6,6	0,0064	20,1	0,0002
IRF5	4,6	0,0007	7,4	6,33E-05
ISLR2	2,7	0,0147	2,5	0,024
ISM2	11,0	6,36E-05	10,0	9,42E-05
ITGA11	2,6	0,0085	4,2	0,0006
ITGA3	6,8	0,0016	25,5	1,79E-05
ITGA6	5,4	0,0066	20,8	7,61E-05
ITGB4	5,7	0,0071	15,8	0,0002
ITGB8	3,9	0,021	7,2	0,0024
ITLN1	9,2	0,0001	21,8	6,54E-06
ITLN2	10,5	0,0145	30,9	0,0013
JPH1	6,4	0,0107	8,2	0,005
KCNA1	3,6	0,0092	5,3	0,0015
KCNA3	3,1	0,0143	8,4	0,0001
KCNA4	8,5	0,0042	13,4	0,0011
KCNAB1	12,5	0,0011	15,3	0,0006
KCNB2	6,1	0,0188	13,1	0,0022
KCNH3	5,8	0,0052	11,7	0,0005
KCNH5	6,4	1,82E-05	18,7	1,57E-07
KCNJ11	2,4	0,0222	2,1	0,0451
KCNJ16	15,6	3,65E-05	17,6	2,42E-05
KCNJ5	11,4	0,0003	9,3	0,0006
KCNK5	8,1	0,0003	16,2	2,57E-05
KCNN4	4,7	0,02	4,8	0,0183
KCNT2	2,6	0,0033	3,3	0,0007

KCTD11	2,4	0,0181	2,4	0,0167
KCTD14	3,6	0,0382	11,5	0,0008
KDR	9,7	0,0001	21,9	6,89E-06
KHDC1	2,6	0,0216	2,4	0,0289
KIAA1024	3,4	0,0177	3,5	0,017
KIAA1211L	11,0	0,0019	12,5	0,0013
KIAA1522	4,5	0,0003	3,5	0,0014
KIAA1671	3,7	0,0038	6,6	0,0003
KLHL31	4,6	0,0032	6,4	0,0007
KLHL4	6,1	0,0004	12,1	2,27E-05
KLK1	7,4	0,0011	19,4	3,87E-05
KLK11	10,2	0,0002	26,1	5,95E-06
KLK5	10,9	0,0071	26,0	0,0008
KLK7	9,3	0,0131	37,4	0,0005
KLK8	6,1	0,0032	7,9	0,0012
KNDC1	3,8	0,0195	3,3	0,0307
KRT16	4,0	0,0401	4,1	0,0354
KRT18	8,8	0,0153	21,4	0,0018
KRT19	9,2	0,0078	24,9	0,0006
KRT5	8,0	0,0146	22,5	0,0011
KRT7	6,9	0,0196	20,7	0,0012
KRT8	8,1	0,0098	22,9	0,0006
KRT80	5,7	0,0039	5,9	0,0036
L1CAM	5,3	0,001	8,8	0,0001
L3MBTL4	2,9	0,0039	3,8	0,0007
LAMA3	5,0	0,0009	3,9	0,0032
LAYN	3,4	0,0114	5,1	0,0019
LCN2	4,4	0,0243	4,3	0,026
LEF1	5,4	0,0194	5,2	0,0213
LGALS2	6,6	0,0131	9,2	0,0052
LGI2	3,9	0,0136	3,1	0,0357
LGR6	11,0	0,0046	17,4	0,0014
LHCGR	4,7	0,0052	11,4	0,0002
LIME1	2,7	0,0336	3,1	0,0184
LIMS2	2,5	0,0243	3,9	0,0021
LINC00665	4,5	0,0005	3,4	0,0022
LINC00672	2,6	0,0109	3,0	0,0046
LINC00842	4,0	0,0445	6,7	0,0096
LINC01018	6,3	0,0027	8,7	0,0008
LINC01105	17,9	0,0003	28,1	8,17E-05
LINC01123	2,8	0,0082	2,6	0,0132
LINC01133	9,9	0,0003	19,9	3,21E-05
LINC01239	3,0	0,0235	7,0	0,0006
LIPC	4,5	0,0029	5,7	0,001
LMNB1	2,1	0,0011	2,6	0,0002
LMNTD2	7,0	0,0011	7,1	0,001
LOC100129291	3,4	0,0004	2,9	0,0012
LOC100507387	4,1	0,0357	9,4	0,0028
LOC101929431	2,3	0,0289	2,6	0,0148
LOC284023	2,6	0,0038	2,2	0,0115
LOC641367	2,9	0,0087	2,2	0,0362
LOC727751	3,6	0,018	17,3	5,61E-05

LOC91450	3,3	0,0129	3,8	0,0076
LOXHD1	8,4	0,0002	20,8	8,91E-06
LOXL1	4,8	0,0003	3,7	0,0012
LRP2	10,7	0,0057	20,0	0,0011
LRP2BP	3,0	0,0132	3,2	0,0087
LRRC1	5,7	0,0004	10,5	3,04E-05
LRRC17	5,5	0,0081	4,3	0,0199
LRRC23	2,1	6,10E-05	2,1	5,52E-05
LRRC36	4,2	0,0045	3,8	0,0073
LRRC38	7,8	8,52E-05	16,8	3,94E-06
LRRC8C	3,6	0,0088	4,6	0,0029
LRRC8D	2,3	0,0104	2,2	0,0145
LRRN4	10,0	0,0026	34,0	8,46E-05
LSR	6,8	0,0034	8,2	0,0018
LTBP1	5,5	0,012	7,6	0,0044
LTC4S	2,6	0,0034	3,6	0,0004
LUZP2	12,4	0,001	6,5	0,0077
LXN	3,0	0,0193	3,8	0,0066
LY6G6C	6,5	0,0119	11,8	0,0021
LY6H	20,3	0,0067	32,3	0,0027
LYPD6	8,1	0,0094	15,8	0,0015
MAL2	6,9	0,0046	15,4	0,0003
MAP3K11	2,4	0,0016	2,3	0,0029
MAP3K9	8,6	0,0018	10,6	0,0009
MAPK14	2,1	0,0359	2,3	0,0258
MAPK15	3,9	0,0015	12,8	5,44E-06
MARCH1	3,5	0,0034	10,9	1,53E-05
MARVELD2	4,0	0,003	4,4	0,0018
MAST4	2,8	0,0016	3,4	0,0005
MATN4	14,1	0,0033	19,9	0,0014
MBNL3	2,3	0,0019	2,9	0,0003
MCTP1	2,4	0,013	2,8	0,0047
MCTP2	8,1	0,0033	12,2	0,0009
MED9	2,2	0,0033	2,1	0,0044
MET	8,5	6,37E-05	17,6	3,76E-06
METTL25	3,6	0,0036	3,6	0,0036
MFAP5	4,5	0,0473	8,2	0,0094
MFSD6	2,7	0,048	4,3	0,0067
MGST2	2,9	0,0006	2,7	0,0012
MID1	2,3	0,0128	2,5	0,0084
MISP	3,1	0,0191	6,8	0,0007
MMP15	6,2	0,0081	5,9	0,0095
MMP24	7,5	0,0003	15,3	1,75E-05
MMP27	5,6	0,0229	7,0	0,012
MOCS3	2,1	0,0001	2,2	7,86E-05
MPP7	5,2	0,0003	10,9	1,12E-05
MPPED2	3,4	0,0012	4,2	0,0003
MPV17L2	2,4	0,0062	2,0	0,0226
MRPL17	2,4	0,0049	2,1	0,014
MRPL49	2,1	0,0144	2,3	0,0091
MSLN	9,5	0,0002	26,7	6,68E-06
MSMO1	3,0	2,83E-05	3,3	1,42E-05

MST1	7,4	0,0004	7,1	0,0004
MSTN	3,7	0,0407	3,5	0,0486
MT3	3,4	0,0086	3,8	0,0047
MTHFD1L	2,2	0,0091	2,7	0,0019
MTMR7	4,1	0,0116	7,2	0,0014
MTMR8	3,6	0,0015	3,9	0,001
MUC1	2,9	0,007	3,2	0,0044
MUC16	8,2	0,0014	26,7	3,23E-05
MUM1L1	7,0	0,0366	27,8	0,0017
MYD88	2,1	3,66E-05	2,1	3,66E-05
MYEF2	2,6	0,0048	2,9	0,0024
MYL4	3,6	0,0003	4,2	0,0001
MYO5B	13,2	6,96E-05	49,9	1,14E-06
MYO5C	7,7	0,0032	18,7	0,0002
MYRF	8,1	0,0045	14,5	0,0008
N4BP3	4,1	0,002	5,2	0,0007
NAP1L3	2,4	0,0444	2,9	0,0211
NCEH1	3,7	0,001	7,6	2,22E-05
NDFIP2	2,2	0,004	2,0	0,0067
NDRG4	4,0	0,0053	8,6	0,0002
NEBL	4,9	0,0231	5,3	0,0175
NEDD9	5,9	0,0011	4,2	0,005
NEK5	6,6	0,0017	4,9	0,0054
NFE2L3	3,6	0,0407	4,3	0,0248
NHSL1	3,3	0,0254	4,5	0,0072
NKAIN1	4,7	9,14E-06	3,3	9,37E-05
NME2	3,3	0,0107	5,2	0,0013
NOS1AP	6,4	0,0023	19,6	5,03E-05
NOVA2	11,7	0,0085	23,2	0,0017
NPHS1	10,9	0,0085	8,4	0,0162
NQO1	2,6	0,002	2,8	0,0011
NRG4	4,5	0,0077	26,1	1,63E-05
NSG1	12,5	0,0005	25,7	5,76E-05
NTM	7,4	9,46E-07	10,5	1,75E-07
NTN1	5,9	0,0099	10,7	0,0014
NTN4	3,5	0,0243	5,3	0,005
NTNG2	4,7	0,0262	7,3	0,0068
NTRK3	3,1	0,0453	4,5	0,0108
OCIAD2	5,5	0,0021	8,7	0,0003
OCLN	7,9	0,0011	19,0	5,59E-05
OLFML2A	6,9	0,0019	8,4	0,0009
OSBP2	4,9	0,0054	10,0	0,0004
OSBPL3	3,5	0,0106	3,6	0,0096
OVCA2	3,9	0,0025	4,3	0,0015
OXTR	2,9	0,0404	5,9	0,0024
P2RX2	6,0	0,0024	6,8	0,0015
PALD1	3,5	0,0128	2,6	0,0431
PAQR5	5,2	0,0165	7,3	0,0054
PAQR9	4,5	0,0003	8,5	1,30E-05
PARD6A	3,4	0,0181	3,4	0,0172
PARD6B	6,7	0,0029	13,8	0,0003
PATJ	2,7	0,0099	3,8	0,0015

PBOV1	6,5	0,0069	8,0	0,0035
PCDH20	6,0	0,0101	8,5	0,0034
PCK1	3,6	0,0091	2,7	0,0326
PCK2	4,1	0,0011	5,5	0,0002
PCLO	9,7	3,79E-05	15,8	5,51E-06
PCOLCE2	2,9	0,0001	2,4	0,0006
PCP4	13,6	0,0005	17,4	0,0003
PDE10A	4,2	0,0003	6,4	3,58E-05
PDE8B	6,0	0,0007	14,4	1,93E-05
PDGFB	3,6	0,0081	3,5	0,0089
PDGFC	6,9	0,0002	6,7	0,0002
PDIA2	3,4	0,0163	8,2	0,0005
PDLIM5	2,4	0,0014	2,6	0,0008
PDZK1IP1	9,5	0,0023	17,1	0,0004
PDZRN4	7,0	0,0011	12,4	0,0001
PGBD3	2,5	0,0016	2,9	0,0005
PHGDH	2,2	0,0186	2,1	0,0201
PI3	5,9	0,0387	12,9	0,0059
PICK1	2,2	0,0122	2,2	0,0094
PIK3CD	2,9	0,0096	2,6	0,0155
PIP5K1B	8,7	0,0019	14,2	0,0004
PKHD1L1	9,7	0,0002	29,5	5,22E-06
PKIA	3,2	0,0359	3,7	0,0207
PKP2	5,7	0,0307	6,0	0,0259
PLA2G16	2,1	0,0049	2,1	0,0051
PLA2G2A	5,1	0,0065	7,9	0,0013
PLA2G7	2,8	0,032	4,1	0,0063
PLCB1	3,6	5,52E-06	6,8	7,59E-08
PLCD1	2,2	0,0113	2,8	0,0021
PLEKHA7	3,2	0,0061	2,8	0,0123
PLEKHB1	7,1	0,0021	10,3	0,0006
PLEKHG4	3,4	0,007	3,4	0,0065
PLEKHG4B	3,8	0,0008	2,7	0,0067
PLLP	10,3	0,0024	27,9	0,0001
PLXNB1	2,3	0,0113	3,5	0,0006
PODXL	8,5	0,0008	29,1	1,33E-05
POF1B	5,6	0,0058	5,8	0,0052
POPDC2	3,7	0,001	4,4	0,0004
PPBP	3,2	0,0054	3,8	0,002
PPM1L	2,0	0,043	2,1	0,04
PPP1R26	2,0	0,0441	2,2	0,0262
PPP4R4	7,0	0,0023	3,8	0,0227
PREX1	2,7	0,0068	3,1	0,003
PRKG2	6,0	0,0011	11,9	7,30E-05
PROCR	3,5	0,0432	5,7	0,008
PROSER2	2,3	0,0066	4,0	0,0002
PRPH	6,2	0,0412	7,3	0,0287
PRR5L	8,8	0,0014	3,5	0,0347
PRR9	5,6	0,001	11,7	4,91E-05
PRSS23	4,8	0,0003	5,4	0,0001
PRSS8	4,6	0,0366	4,2	0,0496
PSG4	6,9	0,0043	12,4	0,0007

PSORS1C1	4,8	0,0121	7,3	0,0029
PTGIS	3,3	0,0012	5,2	8,58E-05
PTGS1	5,3	0,0003	5,3	0,0004
PTH2R	17,1	0,0078	42,5	0,0012
PTHLH	7,7	0,0001	12,3	1,54E-05
PTK2B	4,3	0,0043	4,7	0,003
PTPN14	2,1	0,0068	2,0	0,0083
PTPN3	4,9	0,0019	11,6	5,58E-05
PTPRH	2,6	0,012	4,9	0,0003
PTPRN2	4,7	0,0008	3,1	0,0071
PTPRZ1	5,0	0,0027	12,0	8,89E-05
PXDNL	14,2	0,006	17,6	0,0036
QPCTL	2,2	0,0093	2,1	0,0151
QRFPR	3,5	0,0036	3,2	0,0059
RAB11FIP1	2,9	0,0108	3,0	0,0081
RAB11FIP4	2,1	0,0319	2,7	0,0059
RAB27B	10,1	0,0002	20,5	1,25E-05
RAB32	2,1	0,0048	2,2	0,0029
RAB33A	3,0	0,0032	2,8	0,005
RAB37	16,4	9,52E-05	13,1	0,0002
RAET1E	9,8	0,0003	21,4	1,96E-05
RAET1G	2,3	0,0117	2,0	0,0263
RAP1GAP2	3,0	0,0401	4,6	0,0074
RASEF	3,2	0,0338	4,5	0,0095
RASGRP4	4,2	0,002	7,0	0,0002
RASSF7	10,8	0,0038	18,2	0,0009
RBKS	2,1	0,0016	2,3	0,0006
RBM47	4,7	0,0024	8,0	0,0003
REC8	5,5	0,0025	6,0	0,0017
REEP1	7,6	0,0019	15,7	0,0002
RELL2	3,0	0,0021	2,7	0,0037
RETREG1	8,6	3,72E-05	12,7	7,37E-06
RFFL	2,6	0,0129	2,9	0,0069
RGS4	7,3	0,0069	9,8	0,0029
RHBDL2	9,5	0,0006	12,9	0,0002
RHOC	2,1	0,0035	2,1	0,0031
RHPN2	2,9	0,0076	3,3	0,0032
RIC3	6,9	3,18E-05	22,5	2,28E-07
RIN1	2,7	0,0187	3,0	0,0101
RIPK4	11,2	0,0005	29,0	2,59E-05
RND2	7,7	9,38E-05	9,2	4,15E-05
ROBO4	3,8	0,0003	4,2	0,0001
RORC	8,9	0,0274	20,5	0,0046
RPRM	6,5	0,0006	12,9	3,92E-05
RSPH3	3,4	0,0008	3,2	0,0013
RSPO1	7,0	0,0027	10,3	0,0007
RTN1	5,1	9,02E-05	6,0	3,61E-05
RTN2	3,3	0,004	2,2	0,0367
RTN4RL1	4,1	0,0126	5,5	0,0037
RUNDC1	2,0	0,0307	2,2	0,0152
S100A1	3,6	0,0063	4,1	0,0033
S100A10	2,1	0,0003	2,5	6,44E-05

S100A14	2,1	0,005	2,1	0,0066
S100A16	2,9	0,0176	3,3	0,0103
S100A2	6,4	0,0298	8,9	0,0129
SAMD10	2,8	0,0029	2,8	0,0029
SAMD12	6,9	0,0045	15,5	0,0003
SAMD9	2,7	0,0299	4,2	0,0038
SBK2	6,9	0,0002	7,7	0,0001
SCEL	11,6	0,002	23,5	0,0003
SCG5	2,7	0,0226	2,5	0,0362
SCN2A	4,8	0,015	6,3	0,0062
SCN3B	5,2	0,0017	6,1	0,0009
SCN5A	4,9	0,0188	15,7	0,0005
SCNN1A	4,1	0,0027	4,8	0,0012
SDR42E1	6,1	0,0033	6,6	0,0025
SDSL	5,2	0,0003	6,3	0,0001
SEC11C	4,4	2,18E-05	6,8	1,57E-06
SEMA3B	2,6	0,0004	3,1	8,07E-05
SEMA3C	6,8	0,0002	10,6	2,59E-05
SEMA3F	10,4	0,0045	7,5	0,0112
SEMA4D	2,8	0,0125	3,5	0,0042
SERAC1	3,9	0,0007	5,3	0,0001
SERINC2	3,9	0,0111	4,1	0,0088
SERPINB8	2,8	0,0312	2,9	0,0268
SERPINE3	9,3	0,0003	23,4	1,06E-05
SERTAD4-AS1	2,6	0,045	3,7	0,0094
SERTM1	5,1	0,0059	15,5	0,0001
SGMS2	2,8	0,0106	3,9	0,002
SGO2	6,1	0,0053	9,0	0,0013
SGPP2	12,1	0,002	19,6	0,0005
SH2D4A	5,5	0,0006	5,1	0,0009
SH3BGRL3	2,4	0,0189	2,9	0,0058
SH3BP4	2,6	0,0009	2,9	0,0003
SH3GL2	9,2	0,0017	6,5	0,0055
SH3GLB2	2,3	5,94E-05	3,0	3,85E-06
SH3RF2	2,3	0,0109	5,3	6,69E-05
SH3TC2	2,7	0,0288	5,1	0,0014
SHE	2,2	0,0003	3,0	1,25E-05
SHISA6	15,9	0,0065	29,4	0,0017
SHROOM2	6,3	0,0039	13,4	0,0003
SHROOM3	3,6	0,0254	10,1	0,0006
SHTN1	2,9	0,0002	5,0	5,42E-06
SIAH3	5,0	0,0289	7,4	0,0091
SLAIN1	5,4	0,0009	14,4	1,60E-05
SLC22A23	3,9	0,0009	5,2	0,0002
SLC22A5	2,5	0,023	3,1	0,0075
SLC28A3	10,3	0,0057	44,4	0,0001
SLC35E4	3,3	0,0063	2,7	0,017
SLC39A8	3,6	0,0081	7,2	0,0004
SLC44A5	2,6	0,0364	5,0	0,0018
SLC4A4	9,7	1,56E-07	15,5	1,93E-08
SLC52A3	2,0	0,0043	2,1	0,0036
SLC7A4	3,5	0,0132	3,0	0,0243

SLC8A1-AS1	4,0	0,0309	5,2	0,013
SLC9A3R1	6,4	0,0003	10,0	4,21E-05
SLC9C2	6,3	0,0045	10,1	0,0009
SLFN11	2,1	0,0037	2,3	0,0017
SLITRK4	6,4	0,0099	7,0	0,0078
SLPI	13,1	0,0001	25,0	1,90E-05
SMPD3	11,7	0,0004	42,4	8,87E-06
SMPDL3B	7,0	0,0004	40,6	7,33E-07
SNCA	9,2	0,0031	41,5	4,80E-05
SNCAIP	3,1	0,001	4,4	8,67E-05
SNX24	2,8	0,0024	4,1	0,0002
SOX11	7,0	0,0015	4,7	0,0071
SPINT1	3,0	0,0097	4,0	0,0022
SPRR2C	5,9	0,0036	4,4	0,0109
SPRR2E	18,5	0,0011	13,4	0,0027
SPRR2F	23,7	0,0003	42,3	5,89E-05
SPTBN2	4,7	0,0016	5,5	0,0008
SSC4D	3,4	0,0105	3,5	0,0092
SSTR4	2,9	0,0214	4,3	0,0036
SSX2IP	4,3	0,007	5,4	0,0029
ST6GAL1	5,8	0,0009	9,0	0,0001
STAP2	4,7	0,0008	6,6	0,0002
STIM2	2,4	0,0063	3,8	0,0002
STK17A	2,3	0,0003	2,8	4,77E-05
STK26	4,0	0,0012	5,9	0,0002
STK32B	9,8	0,0005	17,4	7,24E-05
STK33	8,4	0,003	12,5	0,0009
STON2	4,8	0,0035	7,7	0,0005
STRBP	2,6	0,0182	2,3	0,0324
STX11	3,6	0,0068	4,7	0,002
STX1B	2,6	0,0328	3,5	0,0082
STXBP2	8,1	0,0011	16,6	9,64E-05
SULF1	2,7	0,0031	5,4	5,06E-05
SULT1A3	2,1	0,018	3,3	0,001
SULT1A4	2,1	0,018	3,3	0,001
SYN1	3,4	0,0378	3,2	0,0457
SYT17	3,7	0,0485	7,4	0,0054
SYT4	7,1	0,0016	23,9	2,75E-05
TACC2	5,5	0,0117	5,7	0,01
TBC1D15	4,1	0,0205	12,9	0,0004
TBX20	4,1	0,0225	7,5	0,0028
TCAIM	2,3	0,0031	2,3	0,0028
TCEAL2	10,2	0,0073	34,2	0,0004
TCEAL6	8,0	0,0068	9,1	0,0045
TDRD10	2,0	0,0196	2,2	0,0116
TEK	3,7	0,0031	7,0	0,0001
TENM1	8,1	0,0031	15,9	0,0004
TFAP2C	5,8	0,0332	31,0	0,0005
TGM1	10,0	0,0061	29,9	0,0004
TGM2	3,7	0,0029	9,5	3,66E-05
THBD	4,9	0,0076	7,6	0,0015
THSD7B	4,6	0,0031	7,5	0,0004

TLL1	4,4	0,0277	11,2	0,0016
TM4SF1	5,6	0,0048	10,9	0,0004
TM7SF2	5,1	0,0005	5,1	0,0006
TMC1	2,3	0,03	2,9	0,0078
TMC5	9,6	0,0029	16,9	0,0005
TMEM102	2,7	0,0465	5,7	0,0021
TMEM108	5,6	0,0038	5,4	0,0044
TMEM132A	2,3	0,039	3,7	0,0038
TMEM154	4,4	2,17E-07	5,1	7,85E-08
TMEM160	3,0	6,86E-05	2,4	0,0006
TMEM191C	2,8	0,0311	3,6	0,0096
TMEM233	6,4	0,0043	10,1	0,0009
TMEM255A	3,6	0,0001	9,1	4,29E-07
TMEM30B	3,1	0,0031	4,8	0,0003
TMEM35A	6,1	0,0059	5,5	0,0084
TMOD1	4,3	0,0001	5,9	1,78E-05
TMPRSS3	12,8	0,0006	26,1	7,87E-05
TMTC1	2,5	0,0095	2,9	0,0033
TNFRSF11B	6,7	0,0084	5,1	0,0192
TNFRSF21	3,2	8,45E-06	3,4	4,85E-06
TNFRSF8	5,1	0,0167	8,5	0,0033
TNNT1	8,4	0,0042	42,2	4,67E-05
TNNT2	8,3	0,0008	10,6	0,0003
TOM1L1	9,0	0,0072	38,3	0,0002
TPD52	6,5	0,0009	8,8	0,0003
TPD52L1	2,5	0,0139	3,2	0,0027
TPM1	2,4	8,62E-05	2,2	0,0002
TPMT	2,1	0,0022	2,3	0,0008
TPPP3	2,5	0,0089	3,4	0,0012
TRAF5	2,3	0,0017	2,5	0,0007
TRDN	4,5	0,0085	16,0	8,62E-05
TRIM14	5,9	0,0039	15,9	0,0001
TRIM16	3,6	7,44E-05	3,8	5,50E-05
TRIM29	6,1	0,0016	7,4	0,0008
TRIM58	3,6	0,0138	4,4	0,0055
TRMT2B	2,7	0,0179	2,4	0,0282
TRNP1	4,3	0,0078	3,9	0,0113
TSPAN18	4,0	0,0039	7,5	0,0002
TSPAN2	9,9	0,0027	13,4	0,0011
TSPAN33	6,9	0,0027	15,1	0,0002
TSPOAP1	9,2	0,0003	5,6	0,002
TST	4,9	0,0062	3,9	0,0145
TSTD1	6,3	0,0343	13,0	0,006
TTC9	8,0	4,95E-05	5,9	0,0002
TUBB4A	4,1	0,0061	7,3	0,0006
UBXN11	2,8	0,017	3,8	0,0033
UBXN2B	4,9	0,0009	6,6	0,0002
UPK1B	9,4	0,0002	35,6	2,08E-06
UPK3B	10,6	0,0072	25,6	0,0008
USP43	5,7	0,0004	5,9	0,0003
USP54	2,7	0,0031	4,5	0,0001
VAMP8	5,5	0,0003	8,5	3,37E-05

VAT1L	8,1	0,002	11,7	0,0006
VNN1	2,4	0,0141	2,4	0,0141
VPS18	2,5	0,0044	2,3	0,0071
VSIG2	10,0	2,65E-05	25,6	8,14E-07
VSIR	2,8	0,0002	2,9	0,0002
VTN	8,1	0,0024	16,2	0,0003
VWA2	7,6	0,0014	11,9	0,0003
VWA5B1	5,3	0,0277	5,6	0,0233
VWA7	5,4	0,001	11,1	4,92E-05
WDFY4	3,4	0,0117	6,5	0,0006
WDR54	3,2	0,0008	2,3	0,0083
WFDC2	6,6	0,0043	14,2	0,0004
WFDC3	2,8	0,0474	2,8	0,0472
WNK4	2,7	0,0414	6,0	0,0015
WNT10A	10,8	9,20E-07	16,9	1,45E-07
WNT10B	10,6	0,0095	16,6	0,0032
WNT2B	14,4	7,13E-06	22,5	1,46E-06
WSCD2	3,8	0,0449	7,3	0,0063
WWC1	10,0	0,0036	9,4	0,0043
YWHAH	2,4	0,0001	2,3	0,0002
ZBTB7C	3,2	0,0006	5,6	1,66E-05
ZCCHC12	7,9	0,0254	15,4	0,0056
ZFP42	4,3	0,0015	4,4	0,0013
ZNF14	2,3	0,0145	2,6	0,0063
ZNF154	2,4	0,0259	3,5	0,0038
ZNF185	4,0	0,0087	6,9	0,001
ZNF229	2,4	0,0495	6,8	0,0004
ZNF382	2,8	0,0376	3,5	0,0147
ZNF416	2,1	0,0367	3,3	0,003
ZNF420	2,5	0,0143	2,0	0,0487
ZNF461	2,2	0,0034	2,3	0,0029
ZNF473	2,4	0,0022	2,4	0,0028
ZNF502	2,2	0,0052	2,0	0,0098
ZNF551	2,2	0,0025	2,0	0,0067
ZNF567	2,2	0,0056	2,0	0,0102
ZNF582	2,1	0,028	2,1	0,0255
ZNF668	3,8	0,0072	7,6	0,0004
ZNF814	4,6	0,0032	3,7	0,0079
ZNF845	2,1	0,0045	2,2	0,0032

up in -/CD271+ fromVS

Gene name	FC vs -/-	raw P value	FC vs MSCA1+	raw P value
ADCY2	6,2	1,20E-05	2,4	0,0068
BCAN	4,1	0,0023	3,8	0,0063
CD74	2,5	0,001	2,1	0,0011
GPAT3	2,3	0,0266	2,4	0,0371
HPSE2	7,8	0,004	4,6	0,0204
JAM2	2,1	0,0301	2,1	0,0371
MS4A4A	2,7	0,0001	2,7	0,0008
MYOC	8,3	0,0001	3,3	0,0282
OMD	2,2	0,0166	2,2	0,0084

OR1S1	2,2	0,0012	2,2	0,0086
PCDH19	4,5	0,0048	3,3	0,0346
RELN	3,5	0,0027	2,5	0,0357
RNASE6	2,8	0,0189	4,3	0,0019
THBS4	4,8	0,0028	3,1	0,0446
**up in MSCA1+ from VS**				
Gene name	FC vs -/CD271+	raw P value	FC vs -/-	raw P value
ABCA4	3,5	0,0163	3,1	0,0251
ACR	2,4	0,0313	3,7	0,0025
ADAM12	4,2	0,0014	38,5	1,44E-07
ALPL	16,8	9,50E-05	67,8	1,09E-07
APOE	2,5	0,006	17,5	1,55E-05
BIRC5	3,3	0,0154	2,8	0,0124
CCDC3	2,2	0,0032	13,6	6,11E-07
CDKL4	2,1	0,0129	9,6	3,84E-05
CEMIP	9,6	0,0026	23,1	4,96E-05
CGREF1	3,5	0,0048	3,1	0,0135
CIDEC	4,0	0,0388	9,8	0,002
CILP2	2,6	0,0452	19,6	1,65E-06
CNN2	3,1	0,0066	5,9	0,0008
CNTNAP2	19,8	0,0111	27,9	0,0013
COL3A1	2,5	0,0314	3,3	0,0025
COLEC11	2,3	0,0373	11,2	1,12E-05
CPZ	2,6	0,007	4,7	0,0007
EDNRA	2,2	0,0301	3,9	0,0052
ENPP1	2,3	0,0066	5,8	1,69E-05
FAM169A	2,0	0,019	2,4	0,0201
FAM45BP	2,2	0,0367	2,4	0,0093
FAM47B	2,9	0,0095	2,9	0,0013
FHL2	2,7	0,0025	4,7	0,0002
GREM1	2,7	0,0381	50,8	5,46E-06
IFNA13	2,1	0,0244	3,6	0,0007
KCNIP2	3,3	0,0071	3,8	0,0034
KRTAP1-1	2,0	0,0391	2,1	0,0141
LINC00632	4,6	0,0026	2,4	0,0171
LINC00908	2,2	0,0254	2,2	0,0374
LINC01179	3,5	0,0328	6,2	0,0006
LINC01363	3,6	0,0013	3,4	0,001
LINC02021	2,0	0,0154	2,0	0,0336
LOC101928861	5,2	0,001	3,8	0,01
LOC283299	5,6	0,02	4,1	0,0224
LOC729683	3,5	0,0047	3,0	0,0023
LPL	3,5	0,0161	7,9	9,36E-05
MEST	2,1	0,0289	3,5	0,0006
MGC15885	3,2	0,02	7,1	8,29E-05
MMP21	2,6	0,042	7,8	0,0004
MOBP	3,5	0,0074	3,0	0,0123
MRGPRX1	2,7	0,0002	2,3	0,0003
MYOM1	2,5	0,0469	2,8	0,04
NANOS3	3,1	0,0412	3,3	0,0114
NFAM1	2,9	0,0467	3,7	0,0054

NPR3	3,4	0,0492	45,8	4,59E-05
OR4C6	2,7	0,0362	2,2	0,0462
OR4F15	2,6	0,0149	2,1	0,0236
OR4F5	4,2	0,023	3,3	0,0432
OR4M1	3,5	0,0189	3,4	0,0093
OR4P4	5,5	0,0081	5,1	0,0034
OR56A5	5,5	0,0062	3,1	0,0215
OR5J2	7,4	0,0037	4,0	0,0095
OR6C75	2,3	0,0399	2,3	0,0154
PDIA3P1	7,5	0,0367	6,1	0,0487
RAC3	2,7	0,0031	7,6	2,88E-05
REXO1L2P	2,5	0,0478	2,3	0,0154
RHD	2,2	0,0305	2,7	0,0044
SCUBE3	2,4	0,0448	2,5	0,0148
SDC1	3,4	0,003	5,0	0,0007
SGMS1-AS1	4,7	0,0098	3,7	0,0405
SLC38A4	2,8	0,0183	13,3	0,0002
SPATA31C1	3,6	0,0341	3,1	0,0222
SPIB	3,0	0,0241	2,3	0,0457
SSTR5-AS1	2,7	0,0176	2,3	0,0476
TLE6	4,5	0,0446	4,3	0,0329
TMEM178A	4,6	0,0416	6,1	0,0095
TPX2	4,5	0,015	3,6	0,0241
WFS1	2,0	0,0464	3,3	0,0011
ZNF733P	2,7	0,0071	2,4	0,005
