# Supplementary material for: Lobular architecture of human adipose tissue defines the niche and fate of progenitor cells
Source: Nat Commun. 2019 Jun 11;10:2549. doi: 10.1038/s41467-019-09992-3 (PMC6560121; doi:10.1038/s41467-019-09992-3)
Supplement: Supplementary file 2 — Reporting Summary [file 41467_2019_9992_MOESM2_ESM.pdf]

nature research

Corresponding author(s): Jean Galitzky

Last updated by author(s): Mar 6, 2019

Reporting Summary

Nature Research wishes to improve the reproducibility of the work that we publish. This form provides structure for consistency and transparency in reporting. For further information on Nature Research policies, see Authors & Referees and the Editorial Policy Checklist.

Statistics

For all statistical analyses, confirm that the following items are present in the figure legend, table legend, main text, or Methods section.

n/a	Confirmed	
☐	☒	The exact sample size (*n*) for each experimental group/condition, given as a discrete number and unit of measurement
☐	☒	A statement on whether measurements were taken from distinct samples or whether the same sample was measured repeatedly
☐	☒	The statistical test(s) used AND whether they are one- or two-sided<br>*Only common tests should be described solely by name; describe more complex techniques in the Methods section.*
☒	☐	A description of all covariates tested
☒	☐	A description of any assumptions or corrections, such as tests of normality and adjustment for multiple comparisons
☐	☒	A full description of the statistical parameters including central tendency (e.g. means) or other basic estimates (e.g. regression coefficient) AND variation (e.g. standard deviation) or associated estimates of uncertainty (e.g. confidence intervals)
☐	☒	For null hypothesis testing, the test statistic (e.g. *F*, *t*, *r*) with confidence intervals, effect sizes, degrees of freedom and *P* value noted<br>*Give P values as exact values whenever suitable.*
☒	☐	For Bayesian analysis, information on the choice of priors and Markov chain Monte Carlo settings
☐	☒	For hierarchical and complex designs, identification of the appropriate level for tests and full reporting of outcomes
☒	☐	Estimates of effect sizes (e.g. Cohen's *d*, Pearson's *r*), indicating how they were calculated

Our web collection on statistics for biologists contains articles on many of the points above.

Software and code

Policy information about availability of computer code

Data collection	no software was used to generate data
Data analysis	Flow cytometry analyses were performed using DIVA software V6.<br>Statistical analyses were performed using GraphPad Prism (V6).<br>RNA-Seq analyses were performed using Array studio V10.0 and R V3.4.4 (2018-03-15) with mixOmics package V6.3.2.<br>Pathway enrichement analyses were performed using Gene Analytics (Gene Cards Suite) and Gene Ontology Resource.<br>Image analyses were performed using NIS-Elements V4.10 (adipocyte size), ImageJ (Bodipy quantification) and Imaris V8.2 with filament tracer module (Extracellular matrix analyses).

For manuscripts utilizing custom algorithms or software that are central to the research but not yet described in published literature, software must be made available to editors/reviewers. We strongly encourage code deposition in a community repository (e.g. GitHub). See the Nature Research guidelines for submitting code & software for further information.

Data

Policy information about availability of data

All manuscripts must include a data availability statement. This statement should provide the following information, where applicable:

- Accession codes, unique identifiers, or web links for publicly available datasets
- A list of figures that have associated raw data
- A description of any restrictions on data availability

All RNAseq datasets generated and used in this study are available from the NCBI Gene Expression Omnibus (GEO) portal, accession number GSE127222

Field-specific reporting

Please select the one below that is the best fit for your research. If you are not sure, read the appropriate sections before making your selection.

☒ Life sciences ☐ Behavioural & social sciences ☐ Ecological, evolutionary & environmental sciences

For a reference copy of the document with all sections, see nature.com/documents/nr-reporting-summary-flat.pdf

Life sciences study design

All studies must disclose on these points even when the disclosure is negative.

Sample size	Sample size was limited to the number of participants from the cohort and was chosen taken into account human inter-individual heterogeneity. No statistical methods were used to predetermine the total number of samples needed for this study.
Data exclusions	One sample out of 146 from SENADIP cohort has been excluded based on abnormal liver parameters.
Replication	All experiments were performed in n independent replicates. The n number is specified in the text and or the figure legends
Randomization	This study was not randomized.
Blinding	Blinding was not performed for the acquisition and analysis of data.

Reporting for specific materials, systems and methods

We require information from authors about some types of materials, experimental systems and methods used in many studies. Here, indicate whether each material, system or method listed is relevant to your study. If you are not sure if a list item applies to your research, read the appropriate section before selecting a response.

Materials & experimental systems

n/a	Involved in the study	
☐	☒	Antibodies
☒	☐	Eukaryotic cell lines
☒	☐	Palaeontology
☒	☐	Animals and other organisms
☐	☒	Human research participants
☐	☒	Clinical data

Methods

n/a	Involved in the study	
☒	☐	ChIP-seq
☐	☒	Flow cytometry
☒	☐	MRI-based neuroimaging

Antibodies

Antibodies used	Flow cytometry antibodies: V450-CD31 clone WM59 1:20 (BD Biosciences), FITC-CD34 clone AC136 1:20, PE-MSCA1 clone W8B2 1:20, APC-CD271 clone ME20.4-1.H4 1:20 and PE-Vio770-CD14 clone TÜK4 1:100 (Miltenyi Biotec), V500-CD45 clone HI30 1:20, PerCP-CD34 clone 8G12 1:20, FITC-CD9 clone M-L13 1:5, APC-Cy7 CD36 clone 5-271 1:100 (BioLegend).<br>Western blot antibodies: polyclonal rabbit anti-human CD271 (SantaCruz Biotec NGFR p75 (H-92) sc-5634, 1:500), polyclonal rabbit anti-human GLI1 (ThermoFischer PA5-17303, 1:1000), monoclonal mouse anti-human αSMA (DAKO, clone1A4, 1:1000), monoclonal rabbit anti-human GAPDH (Cell Signaling, clone 14C10, 1:1000).<br>Immunofluorescence antibodies: rabbit polyclonal anti-Type I Collagen (Novus NB600-408, 1:100); rabbit polyclonal anti-Type III Collagen (SantaCruz Biotec (H-300) sc28888, 1:100); mouse monoclonal anti-Elastin (Millipore clone 10B8, 1:200); rabbit polyclonal anti-CD271 (Cusa BIO CSB-PA003448, 1:100), rabbit monoclonal anti-CD34 (Epitomics clone EP373Y, 1:100), mouse polyclonal anti-pan Cytokeratin (Abcam C-11 ab7753 from tissue culture supernatant, 1:25) and rabbit monoclonal anti-WT1 (Abcam clone CAN-R9(IHC)-56-2, 1:50)
Validation	Antibody specificities were validated by the vendors, dilutions were tested and adapted to our samples.

Human research participants

Policy information about studies involving human research participants

Population characteristics	All the SENADIP population characteristics are listed in Supplementary Table 1
Recruitment	Obese Patients were women candidate to bariatric surgery. Non-obese women had surgery for gynecologic purposes. All patients were recruited at Louis Mourier Hospital, Colombes, France
Ethics oversight	The protocol was approved by Ministère de la Recherche, direction générale de la recherche et de l'innovation, cellule

Ethics oversight	bioéthique (DC2008-452).<br>Protocol is registered at ClinicalTrials.gov (SENADIP: NCT01525472) and was approved by Comité de Protection des Personnes I.D.F.VII.

Note that full information on the approval of the study protocol must also be provided in the manuscript.

Clinical data

Policy information about clinical studies

All manuscripts should comply with the ICMJE guidelines for publication of clinical research and a completed CONSORT checklist must be included with all submissions.

Clinical trial registration	SENADIP: NCT01525472
Study protocol	Protocol is registered at ClinicalTrials.gov (SENADIP: NCT01525472) and was approved by Comité de Protection des Personnes I.D.F.VII.
Data collection	All patients information and biological parameters are collected based on clinical requirements for surgery
Outcomes	There is no pre-defined outcome for this protocol

Flow Cytometry

Plots

Confirm that:

☒ The axis labels state the marker and fluorochrome used (e.g. CD4-FITC).

☒ The axis scales are clearly visible. Include numbers along axes only for bottom left plot of group (a 'group' is an analysis of identical markers).

☒ All plots are contour plots with outliers or pseudocolor plots.

☒ A numerical value for number of cells or percentage (with statistics) is provided.

Methodology

Sample preparation	Stromal vascular cells where isolated from human subcutaneous and visceral adipose tissues after enzymatic digestion. Cells were incubated with fluorescent-labeled antibodies for cell surface markers or appropriate isotype control. Fluorescence minus one controls were performed and used to control the gating strategy.
Instrument	BD FACS CantoTM II and BD Influx cell sorter
Software	BD Diva Pro V6 (Canto II) and Sortware (Influx)
Cell population abundance	The average abundance of sorted progenitor subsets per 1 000 000 input stromal vascular cells was: subcutaneous AT -/- subset 90 000 cells, -/CD271+ subset 63 000 cells, MSCA1+ subset 73 000 cells, visceral AT -/- subset 77 000 cells, -/CD271+ 123 000 cells, MSCA1+ 23 000 cells.<br>Purity of sorted progenitor subsets was checked by flow cytometry. The average purity of -/-, -/CD271+ and MSCA1+ subset respectively was: 96.3%, 92.9% and 97.1% in the whole progenitor population.
Gating strategy	Gating strategy is described in Figure 2, Supplementary Figures 1, 4 and 5.

☒ Tick this box to confirm that a figure exemplifying the gating strategy is provided in the Supplementary Information.
